# Epilepsy in dentatorubral–pallidoluysian atrophy: A systematic review and meta‐analysis

**DOI:** 10.1111/epi.18700

**Published:** 2025-10-28

**Authors:** Toru Horinouchi, Haruka Ishibashi, Yukako Nakagami, Yoko Kobayashi Takahashi, Takato Akiba, Masaharu Miyauchi, Naohiro Yamamoto, Ryoichi Inoue, Satoshi Kodama, Takafumi Kubota, Naoto Kuroda

**Affiliations:** ^1^ YES‐Japan, the national chapter of the International League Against Epilepsy Young Epilepsy Section (ILAE‐YES) Tokyo Japan; ^2^ Department of Psychiatry and Neurology Hokkaido University Graduate School of Medicine Sapporo Japan; ^3^ Department of Neurology National Hospital Organization Higashihiroshima Medical Center Hiroshima Japan; ^4^ Student Support Center Kyoto University Kyoto Japan; ^5^ Department of Child Neurology National Center of Neurology and Psychiatry Tokyo Japan; ^6^ Pediatrics and Adolescent Medicine Juntendo University Graduate School of Medicine Tokyo Japan; ^7^ Department of Neurosurgery Izumi City General Hospital Osaka Japan; ^8^ Department of Pediatrics Nara Prefecture General Medical Center Nara Japan; ^9^ Department of Neurology Ohio State University Wexner Medical Center Columbus Ohio USA; ^10^ Department of Neurology University of Iowa Hospitals and Clinics Iowa City Iowa USA; ^11^ Department of Neurology Tohoku University Graduate School of Medicine Sendai Japan; ^12^ Department of Epileptology Tohoku University Graduate School of Medicine Sendai Japan; ^13^ Department of Pediatrics Wayne State University Detroit Michigan USA

**Keywords:** antiseizure medication, dentatorubral–pallidoluysian atrophy, progressive myoclonic epilepsy, systematic review, trinucleotide repeat disorders

## Abstract

**Objective:**

Dentatorubral–pallidoluysian atrophy (DRPLA) is a rare autosomal dominant neurodegenerative disease caused by a CAG repeat expansion in the *ATN1* gene. The juvenile onset type often presents with epilepsy, including progressive myoclonic epilepsy (PME). However, evidence on epilepsy in DRPLA remains limited. This systematic review and meta‐analysis aimed to summarize clinical characteristics of DRPLA‐related epilepsy.

**Methods:**

We systematically searched MEDLINE (PubMed), CENTRAL, Embase, Ichushi, and ClinicalTrials.gov for studies on DRPLA‐related epilepsy, following PRISMA (Preferred Reporting Items for Systematic Reviews and Meta‐Analyses) guidelines. The review protocol was registered with the Open Science Framework. Any study design reporting at least one case of DRPLA‐related epilepsy was eligible, including case reports, case series, cohort studies, and clinical trials. Eligible studies underwent screening and full‐text assessment, followed by inclusion in descriptive and meta‐analytic syntheses. Meta‐analyses included only studies reporting ≥5 DRPLA patients.

**Results:**

A total of 181 studies encompassing 1191 patients met the eligibility criteria. DRPLA patients with epilepsy had a younger onset age (16.9 [95% confidence interval (CI) = 13.76–20.76] vs. 45.5 years [95% CI = 42.77–48.47]) and more CAG repeats (66.7 [95% CI = 63.63–69.84] vs. 59.2 [95% CI = 55.67–62.92]) than those without epilepsy. DRPLA patients with epilepsy showed a higher likelihood of paternal versus maternal inheritance (odds ratio = 2.47 [95% CI = .97–6.27]). Focal seizures were frequently observed (40.0%–76.5%) alongside myoclonic and generalized tonic–clonic seizures. Electroencephalographic findings included slow bursts (38.0%), photoparoxysmal responses (36.6%), and interictal epileptiform discharges (77.5%). Giant somatosensory‐evoked potentials, typically seen in PME, were observed in only two patients and absent in 27. Among antiseizure medications, perampanel and levetiracetam were more frequently reported as effective than sodium channel blockers.

**Significance:**

This review synthesizes fragmented evidence on DRPLA‐related epilepsy and highlights key clinical and electrophysiological patterns. Despite limitations from small‐scale studies, these findings support more informed clinical care and underscore the need for larger cohort studies.

## Graphical abstract

Summary of key clinical and electrophysiological characteristics of DRPLA‐related epilepsy from a systematic review and meta‐analysis of 1,191 patients. DRPLA patients with epilepsy showed earlier disease onset, longer CAG repeat expansion, and a tendency toward paternal inheritance. EEG findings frequently included photoparoxysmal responses.

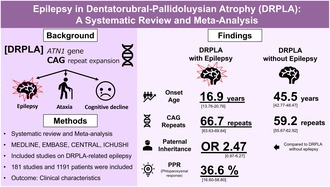

**Key points**

- DRPLA patients with epilepsy have earlier onset and longer CAG repeats than those without epilepsy.
- Focal, myoclonic, and generalized tonic–clonic seizures are common in DRPLA‐related epilepsy.
- A trend toward paternal rather than maternal inheritance was observed in DRPLA with epilepsy.
- Giant somatosensory‐evoked potentials are rare despite the progressive myoclonic epilepsy phenotype.
- Perampanel and levetiracetam are more frequently reported as effective than sodium channel blockers.

## 1 INTRODUCTION

Dentatorubral–pallidoluysian atrophy (DRPLA) is a rare autosomal dominant neurodegenerative disorder caused by a CAG trinucleotide repeat expansion in the *ATN1* gene on chromosome 12p13.31, with an estimated prevalence of .2–.7 per 100 000 individuals.^1^, ^2^, ^3^, ^4^ DRPLA can be broadly categorized into two major clinical phenotypes based on the age at onset: juvenile onset and adult onset.^5^ Among these, the juvenile onset form is often associated with drug‐resistant epilepsy, including progressive myoclonic epilepsy (PME), which has substantial clinical implications.

Despite the clinical significance of epilepsy in DRPLA, existing evidence remains fragmented and poorly characterized. Most reports are confined to small case series or individual case reports, with limited consistency in the description of seizure semiology, electroencephalographic (EEG) findings, and treatment outcomes. The rarity of DRPLA poses a major challenge to prospective, large‐scale investigations, leaving key questions unanswered regarding the epilepsy phenotype, its genetic correlates, and treatment responsiveness.^6^, ^7^, ^8^

To address this gap, we conducted a systematic review and meta‐analysis of the literature on DRPLA‐related epilepsy. This approach enables a comprehensive synthesis of the available data and is particularly well suited for rare disorders, in which large‐scale studies are challenging to conduct. We aimed to summarize the clinicogenomic background, seizure characteristics, electrophysiological findings, and antiseizure medication (ASM) responses in patients with DRPLA‐related epilepsy, with the goal of informing both clinical care and future research.

## 2 MATERIALS AND METHODS

### 2.1 Search strategy

We conducted a systematic review and meta‐analysis in accordance with the Preferred Reporting Items for Systematic Reviews and Meta‐Analyses (PRISMA) guidelines.^9^ The review protocol was registered on the Open Science Framework (https://osf.io/gjdvr/). We systematically searched the following databases from inception to January 26, 2023: MEDLINE (via PubMed), CENTRAL (via the Cochrane Library), Embase, and Ichushi (a major Japanese medical literature database), given the high prevalence of DRPLA reported in Japan.^10^, ^11^, ^12^ To minimize publication bias, we also searched ClinicalTrials.gov for unpublished, ongoing, terminated, or completed studies. In addition, we screened the reference lists of all relevant articles to identify further eligible studies. To ensure the inclusion of the most up‐to‐date studies, the search was updated on January 17, 2025, using the same search strategies and databases. Full search terms for each database are provided in Methods S1.

### 2.2 Eligibility criteria of included studies

We included all published studies reporting original data on human patients with DRPLA‐related epilepsy. The diagnosis of DRPLA was based on each study's own criteria. All study designs were eligible, including case reports, case series, cohort studies, and clinical trials. No restrictions were placed on geographic origin. Studies published in either English or Japanese were considered, given the high prevalence of DRPLA reported in Japan.^10^, ^11^, ^12^

### 2.3 Data extraction, outcome measurement, and assessment of risk of bias

Two reviewers independently screened titles and abstracts in the first screening and assessed the full texts of eligible articles in the second screening. When discrepancies arose between the two reviewers during the screening process, they were first addressed through discussion. If consensus could not be reached, the disagreement was resolved through a meeting involving all authors. The following variables were extracted: first author, publication year, country, language, study type, number of patients, patient characteristics (age at onset, sex, diagnostic method, CAG repeat length, and inheritance pattern), DRPLA‐related clinical features (ataxia, choreoathetosis, extrapyramidal signs, psychiatric symptoms, cognitive impairment), seizure characteristics (defined as including both seizure type and clinical photosensitivity, as indicated by the occurrence of photosensitive seizures), EEG findings, somatosensory evoked potential (SEP) findings, ASM use, and clinical outcomes in patients with DRPLA (hospitalization in DRPLA patients with and without epilepsy, survival time from the clinical onset in DRPLA patients with and without epilepsy, and epilepsy‐related complications such as sudden unexpected death in epilepsy [SUDEP] and drug‐resistant epilepsy/uncontrollable epilepsy in DRPLA patients with epilepsy). Regarding the seizure type, we categorized reported seizures into the following types based on the International League Against Epilepsy (ILAE) 2017 seizure classification: tonic–clonic seizure, myoclonic seizure, tonic seizure, atonic seizure, absence seizure, clonic seizure, focal seizure, and status epilepticus (SE). To summarize and synthesize the data for each seizure characteristic, we calculated both study‐level and patient‐level proportions. The *study‐level proportion* was defined as the proportion of studies reporting each seizure characteristic among all studies reporting seizures in patients with DRPLA‐related epilepsy. The *patient‐level proportion* referred to the proportion of patients with each seizure characteristic, calculated within individual studies. To reduce publication bias from case reports or small case series, patient‐level proportions were derived only from studies that met both of the following criteria: (1) included ≥5 patients with DRPLA‐related epilepsy and (2) provided data on the presence of the seizure characteristic in question. During the course of this project, the ILAE updated the seizure classification in 2025.^13^ Accordingly, we also conducted a systematic review of seizure types based on the ILAE 2025 classification. Regarding the EEG findings, we evaluated three EEG patterns: slow wave bursts, photoparoxysmal responses, and interictal epileptiform discharges (IEDs). Regarding the SEP findings, we evaluated all SEP findings described in the text, regardless of the site of stimulation, recording site, or recording conditions. Regarding ASM use, we collected all studies mentioning the use of the specific ASMs for patients with DRPLA‐related epilepsy, regardless of their dosage. To summarize and synthesize the findings for each ASM, we categorized the effectiveness of each ASM as “effective,” “ineffective,” “adverse effect,” and “seizure worsening,” based on each author's definition. We did not assess the seizure reduction rate of each ASM. Detailed definitions of the extracted variables are provided in Methods S2–S5.

The risk of bias for each study was assessed by two independent reviewers using the appropriate Joanna Briggs Institute critical appraisal checklist (https://jbi.global/critical‐appraisal‐tools), selected according to study design (e.g., case reports, case series, case–control studies, cohort studies, or analytical cross‐sectional studies). Any disagreements regarding bias assessment were resolved by discussion. For classification purposes, we defined case reports as studies including ≤4 patients in total, and case series as those including ≥5 patients, irrespective of whether the patients had DRPLA or another condition.^14^

### 2.4 Statistical analysis

We conducted a systematic review with descriptive analysis for the following outcomes: seizure characteristics in DRPLA‐related epilepsy (tonic–clonic, myoclonic, tonic, atonic, absence, clonic, focal seizures, SE, and photosensitive seizures); SEP findings in patients with epilepsy; outcomes of ASM treatment and non‐ASM treatment (effectiveness, ineffectiveness, adverse effects, and seizure worsening); and clinical outcomes in patients with DRPLA, including hospitalization in DRPLA patients with and without epilepsy, survival time from the clinical onset in DRPLA patients with and without epilepsy, and epilepsy‐related complications such as SUDEP and drug‐resistant epilepsy/uncontrollable epilepsy. In addition, we conducted a systematic review followed by meta‐analysis of 20 outcomes, including (1) demographic and genetic characteristics (sex ratio, age at onset, CAG repeat length) in patients with and without epilepsy; (2) DRPLA‐related clinical features (ataxia, choreoathetosis, extrapyramidal signs, psychiatric symptoms, cognitive impairment) in patients with and without epilepsy; (3) the odds ratio of developing epilepsy according to paternal versus maternal inheritance; and (4) EEG findings (slow bursts, photoparoxysmal responses, and interictal epileptiform discharges) in patients with epilepsy. Duplicate cases were excluded when identifiable, namely, when cases were explicitly reported in other published studies.

For meta‐analysis, we used random‐effects models with the DerSimonian–Laird estimator to account for between‐study heterogeneity. Pooled proportions were calculated using the variance‐stabilizing Freeman–Tukey double arcsine transformation. Confidence intervals (CIs) for individual studies were computed using the Wilson score method with continuity correction. Heterogeneity was assessed using the *I* ^2^ statistic and Cochran *Q* test. *I* ^2^ values of <25%, 25%–49%, 50%–74%, and ≥75% were considered to represent very low, low, moderate, and high heterogeneity, respectively.^15^ A *p*‐value of <.10 by Cochran *Q*‐test was considered indicative of significant heterogeneity.^16^, ^17^ Publication bias was assessed using funnel plots and Egger test for funnel plot asymmetry, with *p* < .10 indicating significant bias.^17^, ^18^ Meta‐analyses with fewer than 10 studies were not evaluated for publication bias. All analyses were conducted in R (R Development Core Team, 2019), using the “meta” and “metafor” packages for proportions and “metafor” for odds and sex ratios. To minimize publication bias due to small sample sizes, we included only studies reporting each outcome for ≥5 patients in the meta‐analysis.^14^ To assess robustness, we performed sensitivity analyses including studies with thresholds of ≥3, ≥4, ≥6, and ≥10 patients. We additionally performed meta‐analyses restricted to studies published in English, using thresholds of ≥3, ≥4, ≥5, ≥6, and ≥10 patients.

Regarding survival time from the onset of DRPLA, we constructed Kaplan–Meier curves and performed a log‐rank test to compare survival between patients with and without epilepsy. For these analyses, only cases with both the age at onset of DRPLA and the age at last observation (either survival or death) available were included.

## 3 RESULTS

### 3.1 Summary of included studies

**FIGURE 1:**
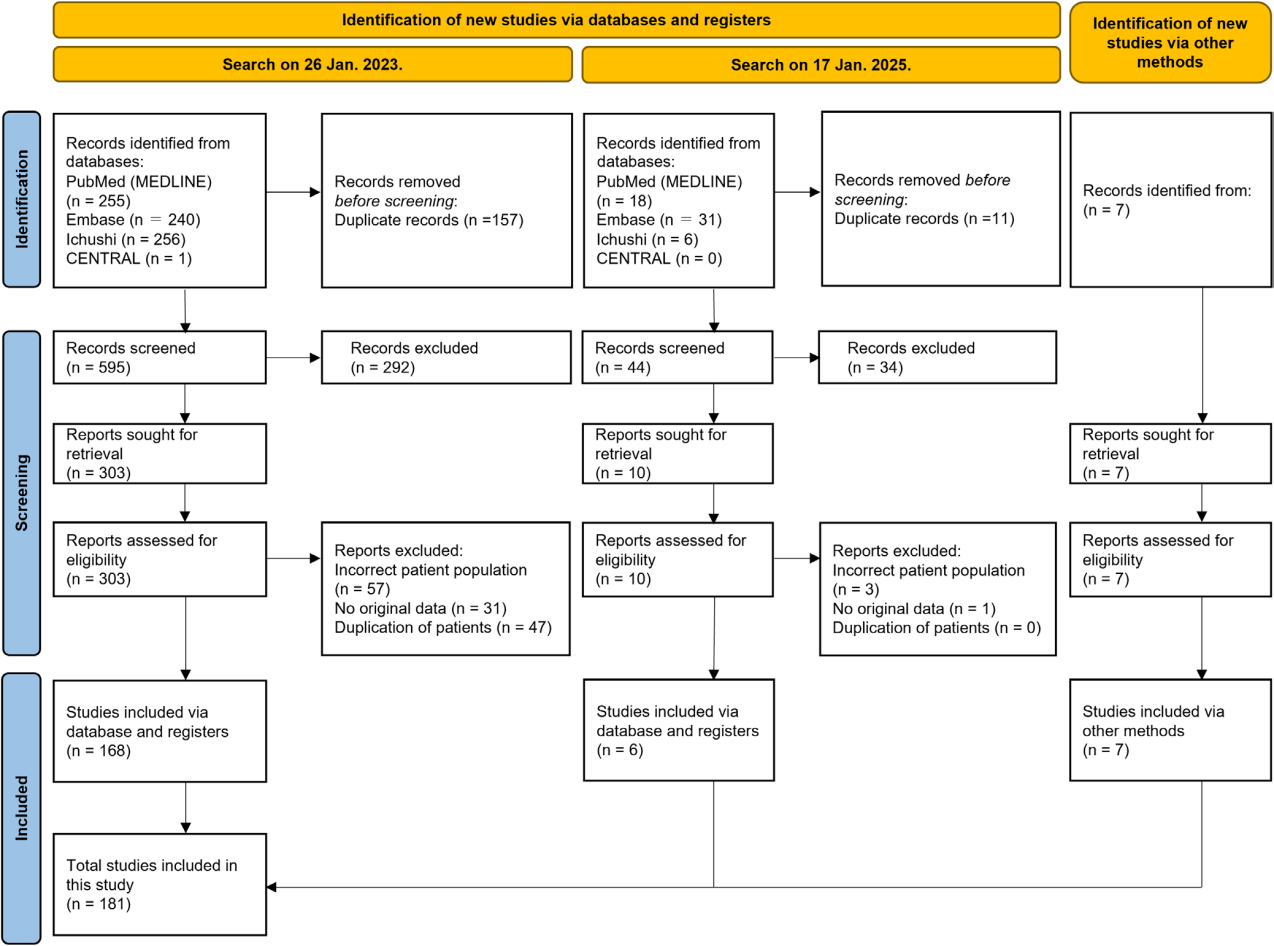
PRISMA (Preferred Reporting Items for Systematic Reviews and Meta‐Analyses) flowchart of the study. A database search was conducted twice; 752 studies were identified on January 26, 2023, and 55 additional studies were identified on January 17, 2025, to update the literature search. Seven more studies were added through other sources. After screening and full‐text assessment, 181 studies (100 in English and 81 in Japanese) involving 1191 patients met the eligibility criteria for this systematic review.

The PRISMA flow diagram is presented in Figure 1. After screening and full‐text review, 181 studies (100 in English and 81 in Japanese), encompassing a total of 1191 patients, met the eligibility criteria for this systematic review. These included three cohort studies, 14 case–control studies, six cross‐sectional studies, 48 case series, and 110 case reports. An overview of the included studies and their main characteristics is provided in Table S1. The mean risk‐of‐bias scores (±SD) were 5.33 ± 3.06 (out of 11) for cohort studies, 4.07 ± 1.73 (out of 10) for case–control studies, 3.33 ± 2.16 (out of eight) for cross‐sectional studies, 3.41 ± 1.61 (out of 10) for case series, and 4.68 ± 2.05 (out of eight) for case reports. A summary of the key findings from this systematic review and meta‐analysis is presented in Table 1.

**TABLE 1:** Overview of the main findings from this systematic review and meta‐analysis.

Characteristic	DRPLA with epilepsy	DRPLA without epilepsy
Clinicogenomic background characteristics		
Sex ratio, male/female^a^	1.04 (95% CI = .79–1.36)	1.17 (95% CI = .81–1.68)
Onset age of DRPLA^a^	16.90 years (95% CI = 13.76–20.76)	45.53 years (95% CI: 42.77–48.47)
Number of CAG repeats^a^	66.66 repeats (95% CI = 63.63–69.84)	59.18 repeats (95% CI: 55.67–62.92)
Ataxia	94.51% (95% CI = 87.85–99.03)	91.73% (95% CI: 80.65–99.00)
Choreoathetosis	62.88% (95% CI = 48.48–76.37)	54.87% (95% CI: 38.31–71.01)
Extrapyramidal signs	20.37% (95% CI = .81–50.93)	10.26% (95% CI: .00–32.33)
Psychiatric symptoms	50.78% (95% CI = 25.90–75.49)	46.15% (95% CI: 27.93–64.81)
Cognitive impairment	95.34% (95% CI = 87.71–99.78)	67.11% (95% CI: 51.61–81.24)
Paternal inheritance^a^	Odds ratio = 2.47 (95% CI = .97–6.27)	
Seizure characteristics^b^		
Tonic–clonic seizure	60.4%; 40.0%–100%	NA
Myoclonic seizure	36.0%; 16.7%–100%	NA
Tonic seizure	15.8%; 9.1%–20.0%	NA
Atonic seizure	12.2%; 9.1%–77.8%	NA
Absence seizure	12.2%; 9.1%–35.3%	NA
Clonic seizure	10.1%; 20.0%	NA
Focal seizure	15.8%; 40.0%–76.5%	NA
Status epilepticus	9.4%; 9.1%–20.0%	NA
Photosensitive seizure	5.0%; 20.0%	NA
Electrophysiology		
Slow bursts on EEG^a^	37.98% (95% CI = 1.16–85.14)	NA
Photoparoxysmal responses on EEG^a^	36.56% (95% CI = 16.60–58.80)	NA
Interictal epileptiform discharges on EEG^a^	77.49% (95% CI = 62.15–90.29)	NA
Prolonged CCT in SEP	5 studies with 7 patients	NA
Small N20 amplitude in SEP	1 study with 3 patients	NA
Prolonged N20 latency in SEP	1 study with 3 patients	NA
C‐reflex in SEP	1 study with 2 patients	NA
Giant SEP	1 study with 2 patients	NA
No giant SEP	6 studies with 27 patients	NA
ASM treatment		
Relatively effective	LEV and PER	NA
Relatively ineffective	Sodium channel blockers	NA
Clinical outcomes		
Survival time from the clinical onset	19 years (95% CI = 17.2–20.8)	15 years (95% CI = 13.9–16.1)

Abbreviations: ASM, antiseizure medication; CCT, central conduction time; CI, confidence interval; DRPLA, dentatorubral–pallidoluysian atrophy; EEG, electroencephalogram; LEV, levetiracetam; NA, not applicable; PER, perampanel; SEP, somatosensory evoked potential.

^a^ Meta‐analysis was conducted.

^b^ Values are shown as study‐level pooled proportion; patient‐level proportion.

### 3.2 Clinicogenomic background characteristics: Meta‐analysis

A meta‐analysis of 30 studies found no significant association between sex and the presence of epilepsy in patients with DRPLA (Figure S1). The male‐to‐female ratio was 1.04 (95% CI = .79–1.36) in patients with epilepsy and 1.17 (95% CI = .81–1.68) in those without epilepsy. Heterogeneity was very low in each group (*I* ^2^ = 5.6%, *p* = .38 in patients with epilepsy; *I* ^2^ < .01%, *p* = .92 in those without epilepsy). No clear evidence of publication bias was observed in the epilepsy group (Egger test: *p* = .44). In the nonepilepsy group, however, a potential publication bias was suggested (Egger test: *p* = .04).

A meta‐analysis of 30 studies revealed a significant difference in age at onset between DRPLA patients with and without epilepsy (Figure S2). The pooled mean age was 16.90 years (95% CI = 13.76–20.76) for patients with epilepsy and 45.53 years (95% CI = 42.77–48.47) for those without. This difference was considered statistically significant, as the 95% CIs did not overlap between the two groups. Heterogeneity was high in the epilepsy group (*I* ^2^ = 90.6%, *p* < .01) and moderate in the nonepilepsy group (*I* ^2^ = 64.0%, *p* < .01). Publication bias was suggested only in the nonepilepsy group (Egger test: *p* = .51 in the epilepsy group and *p* < .01 in the nonepilepsy group).

A meta‐analysis of 12 studies showed a significant difference in the number of CAG repeats (Figure S3). Patients with epilepsy had 66.66 repeats (95% CI = 63.63–69.84), compared to 59.18 repeats (95% CI = 55.67–62.92) in those without epilepsy. This difference was considered statistically significant, as the 95% CIs did not overlap between the two groups. Heterogeneity was high in both groups (*I* ^2^ > 90%, *p* < .01). Publication bias was not assessed due to the small number of included studies in each group (<10).

A meta‐analysis of 27, 24, 12, and 17 studies found no significant difference in the prevalence of ataxia, choreoathetosis, extrapyramidal signs, and psychiatric symptoms between DRPLA patients with and without epilepsy (Figures S4–S7). The prevalence of ataxia was 94.51% (95% CI = 87.85–99.03, *I* ^2^ = 40.3%, *p* = .03) in DRPLA patients with epilepsy and 91.73% in patients without epilepsy (95% CI = 80.65–99.00, *I* ^2^ = 47.5%, *p* = .02), overlapping the 95% CIs between the two groups. No clear evidence of publication bias was observed in both groups (Egger test: *p* = .12 in the epilepsy group and *p* = .21 in the nonepilepsy group). The prevalence of choreoathetosis was 62.88% (95% CI = 48.48–76.37, *I* ^2^ = 64.6%, *p* < .01) in DRPLA patients with epilepsy and 54.87% in patients without epilepsy (95% CI = 38.31–71.01, *I* ^2^ = 54.7%, p < .01), overlapping the 95% CIs between the two groups. No clear evidence of publication bias was observed in both groups (Egger test: *p* = .25 in the epilepsy group and *p* = .51 in the nonepilepsy group). The prevalence of extrapyramidal signs was 20.37% (95% CI = .81–50.93, *I* ^2^ = 87.8%, *p* < .01) in DRPLA patients with epilepsy and 10.26% in patients without epilepsy (95% CI = .00–32.33, *I* ^2^ = 62.5%, *p* < .01), overlapping the 95% CIs between the two groups. Publication bias was not assessed due to the small number of included studies in each group (<10). The prevalence of psychiatric symptoms was 50.78% (95% CI = 25.90–75.49, *I* ^2^ = 84.4%, *p* < .01) in DRPLA patients with epilepsy and 46.15% in patients without epilepsy (95% CI = 27.93–64.81, *I* ^2^ = 49.4%, *p* = .03), overlapping the 95% CIs between the two groups. Publication bias was suggested only in the nonepilepsy group (Egger test: *p* = .99 in the epilepsy group and *p* < .01 in the nonepilepsy group).

A meta‐analysis of 25 studies revealed a significant difference in the prevalence of cognitive impairment between DRPLA patients with and without epilepsy (Figure S8). The prevalence of cognitive impairment was 95.34% (95% CI = 87.71–99.78, *I* ^2^ = 53.1%, *p* < .01) in DRPLA patients with epilepsy and 67.11% in patients without epilepsy (95% CI = 51.61–81.24, *I* ^2^ = 49.5%, *p* = .02), without overlapping the 95% CIs between the two groups. No clear evidence of publication bias was observed in both groups (Egger test: *p* = .11 in the epilepsy group and *p* = .74 in the nonepilepsy group).

A meta‐analysis of nine studies suggested that paternal, compared to maternal, inheritance was associated with a higher likelihood of developing epilepsy among patients with DRPLA (odds ratio = 2.47, 95% CI = .97–6.27; Figure S9), although the CI crossed 1. Heterogeneity was low (*I* ^2^ = 35.5%, *p* = .14). Publication bias was not assessed due to the small number of studies (<10).

### 3.3 Seizure characteristics: Systematic review

A total of 139 studies reported seizures in patients with DRPLA‐related epilepsy. Among these, the number of studies describing seizures in one, two, three, four, and five or more patients were 77, 28, 17, 6, and 11, respectively. However, 26 studies provided only general descriptions of seizures without specifying seizure characteristics and were thus excluded from analyses of seizure characteristics.

**FIGURE 2:**
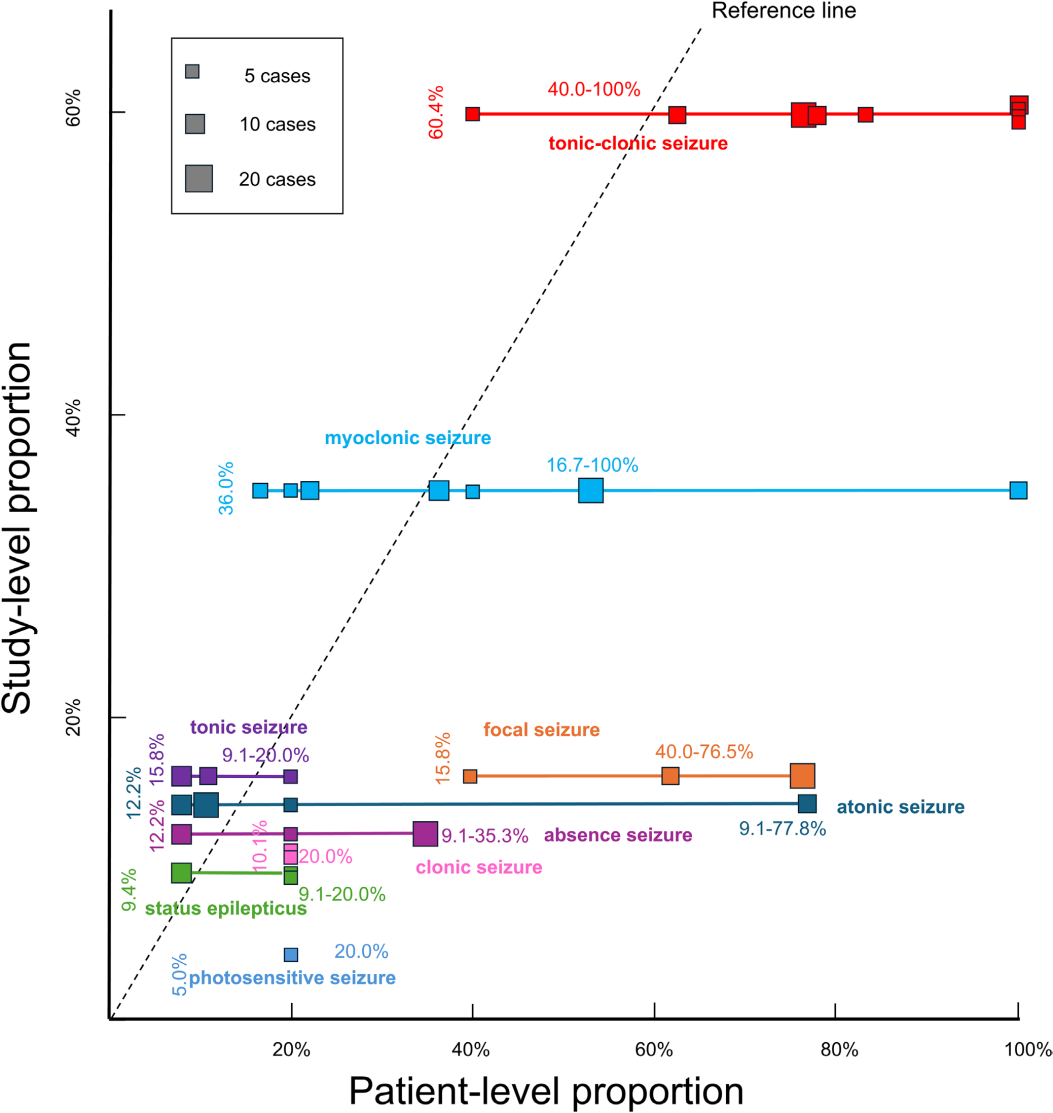
Relationship between the study‐level proportions and the patient‐level proportions of each seizure characteristic. This figure illustrates the study‐level and patient‐level proportions of each seizure characteristic. The *study‐level proportion* (plotted on the y‐axis and indicated by the vertical number in each color) is defined as the proportion of studies that reported each specific seizure characteristic among the 139 included in this systematic review. The *patient‐level proportion* (plotted on the x‐axis and indicated by the horizontal number in each color) is defined as the proportion of patients with each specific seizure characteristic within individual studies. The patient‐level proportion was calculated only from studies that (1) included five or more patients with DRPLA‐related epilepsy and (2) provided data on the presence of the seizure characteristic in question. Each square represents a single study, and the size of the square reflects the total number of reported patients in that study, which serves as the denominator for the patient‐level proportion. Each color corresponds to a specific seizure characteristic. A reference line (shown as a black dotted line) indicates where the study‐level proportion equals the patient‐level proportion.

Figure 2 illustrates the study‐level and patient‐level proportions for each seizure characteristic. Notably, focal, clonic, and photosensitive seizures exhibited marked discrepancies between the two metrics, with patient‐level proportions substantially higher than study‐level proportions. Additional details were available for SE; three cases were classified as convulsive SE, two as myoclonic SE, one as absence SE, one as partial SE, and eight as unspecified SE. The seizure types of photosensitive seizure included tonic, clonic, myoclonic, and generalized convulsive seizures induced by photic stimulation.^19^, ^20^, ^21^, ^22^ In addition, Figure S10 presents a systematic review restricted to studies published in English, and Figure S11 presents a systematic review based on the ILAE 2025 seizure classification, as sensitivity analyses.

### 3.4 Electrophysiology: Meta‐analysis of EEG findings and systematic review of SEP findings

A total of four, five, and eight studies were included in the meta‐analysis for slow bursts, photoparoxysmal responses, and IEDs, respectively. A meta‐analysis of EEG findings in patients with DRPLA‐related epilepsy showed that the pooled proportions of slow bursts, photoparoxysmal responses, and IEDs were 37.98% (95% CI = 1.16–85.14, *I* ^2^ = 89.7%, *p* < .01), 36.56% (95% CI = 16.60–58.80, *I* ^2^ = 57.9%, *p* = .05), and 77.49% (95% CI = 62.15–90.29, *I* ^2^ = 35.8%, *p* = .14), respectively (Figure S12). To gain a deeper understanding of IEDs in DRPLA patients with epilepsy, we examined whether they were focal or generalized, assessed the presence of generalized polyspikes or slow spike‐and‐wave patterns, and, in cases of focal IEDs, evaluated potential regional specificity. Of the 232 cases with clearly documented EEG findings, IEDs were identified in 166. Among these, 87 cases exhibited generalized IEDs and 65 cases exhibited focal IEDs. More specifically, focal IEDs were observed in the frontal region in 14 cases (including findings at boundaries such as the frontotemporal area), in the temporal region in 10 cases (including boundaries such as the frontotemporal area), in the parietal region in 14 cases (including boundaries such as the parieto‐occipital area), in the occipital region in 15 cases (including boundaries such as the parieto‐occipital area), and in the central region in eight cases (including boundaries such as the centrotemporal area). In addition, generalized polyspikes were observed in seven cases, and slow spike‐and‐wave patterns were observed in seven cases.

A systematic review of SEP findings included 21 studies, reporting on 81 patients with DRPLA, of whom 49 had epilepsy. No study directly compared SEP findings between patients with and without epilepsy. Among patients with DRPLA‐related epilepsy, the following abnormalities were reported: prolonged central conduction time (defined as the latency between cervical spinal cord and primary somatosensory cortex responses^23^) in seven patients across five studies; reduced N20 amplitude (representing primary somatosensory cortex response^24^) in three patients from one study; prolonged N20 latency in three patients from one study; and the presence of C‐reflex (an electrically evoked myoclonic response following a giant SEP, reflecting cortical excitability^25^) in two patients from one study. Giant SEPs (considered indicative of cortical hyperexcitability^26^) were reported in two patients from one study, whereas six studies including 27 patients with epilepsy reported the absence of giant SEPs. As a sensitivity analysis, Results S1 presents the results of a systematic review of SEP findings, restricted to studies published in English.

### 3.5 Treatment outcomes for epilepsy in DRPLA: Systematic review

**FIGURE 3:**
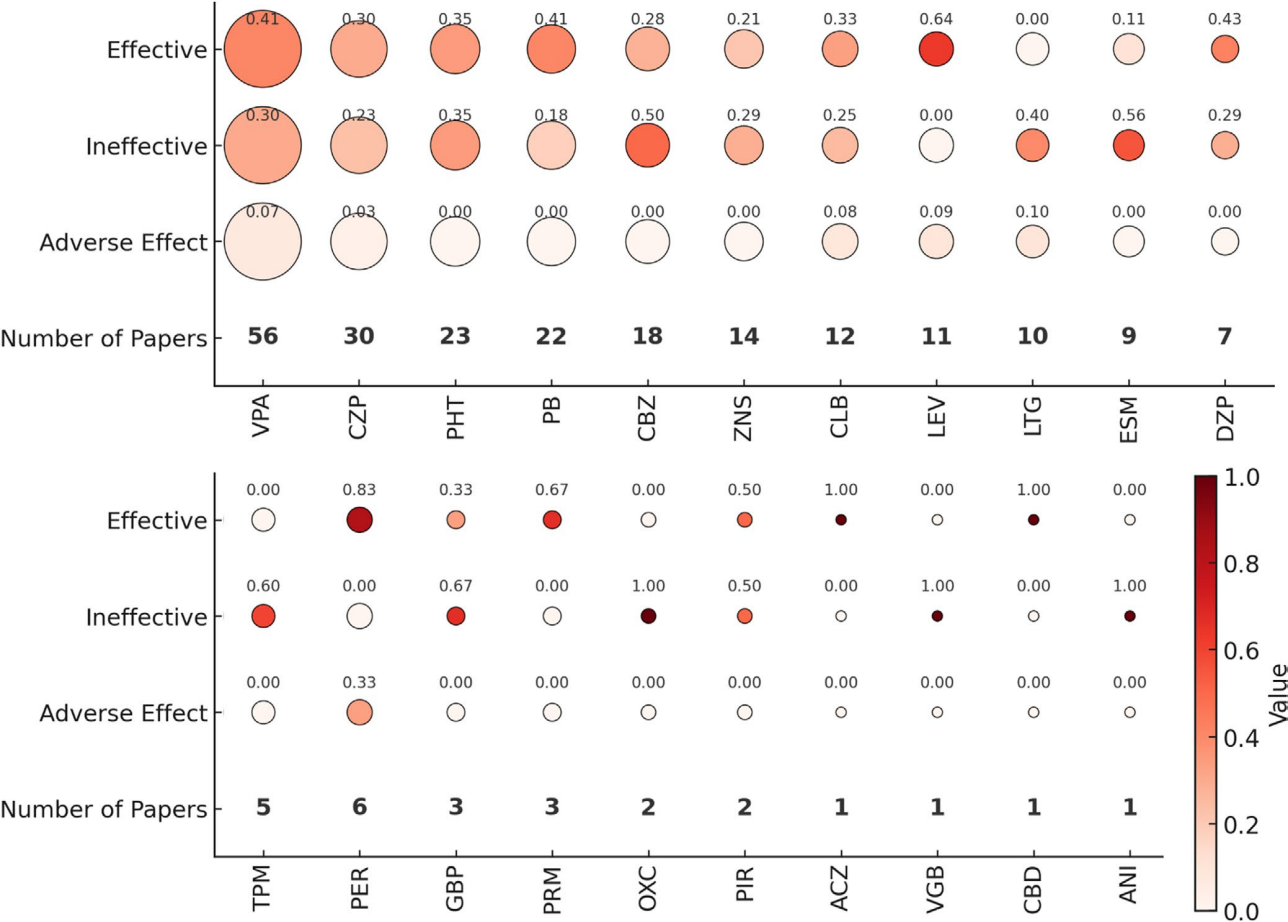
Relationship between the number of studies reporting the use of specific antiseizure medications (ASMs) and the number of studies reporting them as effective, ineffective, or associated with adverse effects. The size of each circle represents the number of studies reporting the use of that ASM. The numbers shown above each circle indicate the proportion of studies reporting the ASM as effective, ineffective, or associated with adverse effects. The color of each circle also reflects these proportions. ACZ, acetazolamide; ANI, aniracetam; CBD, cannabidiol; CBZ, carbamazepine; CLB, clobazam; CZP, clonazepam; DZP, diazepam; ESM, ethosuximide; GBP, gabapentin; LEV, levetiracetam; LTG, lamotrigine; OXC, oxcarbazepine; PB, phenobarbital; PER, perampanel; PHT, phenytoin; PIR, piracetam; PRM, primidone; TPM, topiramate; VGB, vigabatrin; VPA, valproic acid; ZNS, zonisamide.

Among the 72 studies reporting the use of ASMs in patients with DRPLA‐related epilepsy, the most frequently reported treatments were valproic acid (56 studies), followed by clonazepam (30), phenytoin (23), phenobarbital (22), carbamazepine (18), zonisamide (14), and clobazam (12). Figure 3 illustrates the relationship between the number of studies reporting the use of each ASM and the number of studies reporting effectiveness, ineffectiveness, or adverse effects. Among ASMs reported in ≥5 studies, only levetiracetam (64%, 7/11 studies) and perampanel (83%, 5/6 studies) were described as effective in ≥60% of the studies. Adverse effects were reported in four studies for valproic acid, including elevated blood ammonia, liver damage accompanied by drowsiness, renal tubular damage, and elevated cerebrospinal fluid γ‐aminobutyric acid levels. For perampanel, two studies described adverse effects such as sleepiness; and dizziness, weight gain, hallucinations, palpitations, and sleepiness. In addition, adverse effects were each reported in one study for clonazepam (dysphagia), clobazam (movement abnormalities and sleeplessness), levetiracetam (sleepiness), and lamotrigine (rash). Worsening of seizures was reported in two studies for valproic acid (3.6%, 2/56 articles) and in one study for carbamazepine (5.6%, 1/18 articles). As a sensitivity analysis, Figure S13 presents a systematic review restricted to studies published in English.

Additionally, we conducted a systematic review of non‐ASM treatments for DRPLA‐related epilepsy. We found one report each on ketogenic diet, vagus nerve stimulation (VNS), and thyrotropin‐releasing hormone (TRH) therapy. In one case, a ketogenic diet was implemented, and it was reported that myoclonic seizures, as well as subsequent generalized tonic and tonic–clonic seizures, were alleviated following its initiation. Two patients received VNS; in one case, the effect was not clearly described, whereas in the other, seizures were reported to be somewhat controlled in combination with ASMs. In one case, TRH therapy was administered, after which generalized tonic–clonic seizures were reported to have disappeared.

### 3.6 Clinical outcomes in patients with DRPLA

Regarding hospitalization, 111 among 695 (16.0%) DRPLA patients with epilepsy and 15 among 358 (4.2%) DRPLA patients without epilepsy were reported to be hospitalized, including for the purposes of diagnostic evaluation, therapeutic interventions for DRPLA, and management of complications or comorbid conditions.

**FIGURE 4:**
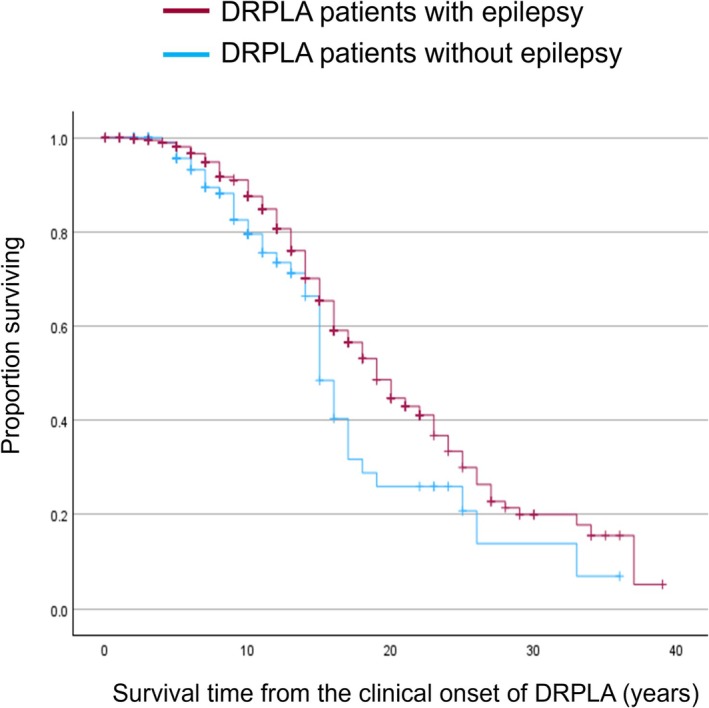
Kaplan–Meier survival curves in dentatorubral–pallidoluysian atrophy (DRPLA) patients with and without epilepsy. Survival times from the clinical onset of DRPLA were compared between patients with epilepsy (*n* = 386) and those without epilepsy (*n* = 110).

Regarding survival time from the onset of DRPLA, 386 patients with epilepsy and 110 patients without epilepsy were included in the analysis. The median survival time from onset was 19 years (95% CI = 17.2–20.8) in patients with epilepsy and 15 years (95% CI = 13.9–16.1) in those without epilepsy. Figure 4 illustrates the Kaplan–Meier curves. The log‐rank test demonstrated significantly longer survival in patients with epilepsy compared to those without epilepsy (*χ* ^2^ = 5.37, *p* = .020). Notably, three cases with DRPLA‐related epilepsy were reported as SUDEP. As a sensitivity analysis, Results S2 presents the results of the survival time analysis, restricted to studies published in English.

Regarding drug‐resistant epilepsy and uncontrollable seizures, information on epilepsy or seizure control was unavailable for 72.7% (505/695) of patients with DRPLA‐related epilepsy. Among the 190 patients with available descriptions, 33.7% (64/190) had drug‐resistant epilepsy and 74.2% (141/190) had uncontrollable seizures.

### 3.7 Sensitivity meta‐analyses

The results of the sensitivity meta‐analyses, which included studies reporting each outcome for (1) ≥3 patients, (2) ≥4 patients, (3) ≥6 patients, and (4) ≥10 patients, instead of the primary threshold of ≥5 patients, are presented in Table S2. In addition, Table S3 presents a systematic review and meta‐analysis restricted to studies published in English.

## 4 DISCUSSION

### 4.1 Summary

This comprehensive systematic review and meta‐analysis summarizes the clinicogenomic background characteristics, seizure characteristics, electrophysiological findings, and ASM use in patients with DRPLA‐related epilepsy. A total of 1191 patients with DRPLA were identified across 181 studies. Due to the rarity of DRPLA, most of the included studies were small in scale, with 158 studies (87.2%) consisting of case reports or case series. Notably, 81 studies (44.8%) were written in Japanese. This high proportion can be explained by the inclusion of Ichushi, the Japanese medical literature database, and the historical context in which the disease was first proposed and its causative gene first identified in Japan.^1^, ^2^, ^27^

### 4.2 Clinicogenomic background characteristics of patients with DRPLA‐related epilepsy

The meta‐analysis revealed that patients with DRPLA‐related epilepsy had a younger age at onset and a higher number of CAG repeats compared to those without epilepsy. No significant difference was found in the sex ratio between the two groups. Although the associations between paternal inheritance and epilepsy did not reach statistical significance, a tendency toward paternal transmission was observed. These findings are consistent with previous studies reporting that (1) paternal inheritance is associated with a greater number of CAG repeats than maternal inheritance,^1^, ^2^ (2) a higher number of CAG repeats correlates with earlier onset of DRPLA,^1^, ^2^ and (3) DRPLA‐related epilepsy occurs more frequently in juvenile onset than in adult onset disease.^5^ Regarding clinical manifestations, no significant differences were observed between patients with and without epilepsy in terms of ataxia, choreoathetosis, extrapyramidal signs, or psychiatric symptoms, whereas cognitive impairment was significantly more frequent in those with epilepsy. In general, these manifestations are known to occur in both juvenile and adult onset DRPLA.^5^ The observed difference in cognitive impairment may be explained by the predominance of more severe phenotypes in juvenile onset cases, in which such symptoms are more pronounced and therefore more likely to be recognized at an earlier stage compared to adult onset cases.

### 4.3 Seizure characteristics of patients with DRPLA‐related epilepsy

The systematic review confirmed a broader spectrum of seizure characteristics in DRPLA‐related epilepsy beyond the commonly recognized myoclonic seizures and generalized tonic–clonic seizures. As shown in Figure 2, focal, clonic, and photosensitive seizures were underrepresented in the overall number of studies but constituted a substantial proportion within the subset of studies that explicitly assessed them. One possible explanation is that these seizure characteristics in DRPLA may be underrecognized or underreported. Regarding focal seizures, they have been reported to occur more frequently in patients with earlier epilepsy onset.^6^ Among six reported patients, four had seizures arising from the occipital lobe (including one with bilateral occipital foci), and two had seizures arising from the frontal lobe (including one with bilateral frontal foci). No seizures originating from the temporal or parietal lobes were reported.^6^ However, the underlying mechanisms of focal seizures remain unclear.

Regarding SE, our review identified not only convulsive SE but also myoclonic and absence SE. This diversity suggests that DRPLA‐related epilepsy encompasses a wide spectrum of SE manifestations. Clinicians should be aware of atypical forms such as myoclonic or absence SE, which may otherwise be underrecognized in the diagnostic and treatment process.

### 4.4 Electrophysiological findings in patients with DRPLA‐related epilepsy

This study conducted meta‐analyses of EEG findings, including slow bursts, photoparoxysmal responses, and IEDs. Notably, the estimated prevalence of photoparoxysmal responses in patients with DRPLA‐related epilepsy was 36.56% (95% CI = 16.60–58.80), which appears higher than that observed in idiopathic generalized epilepsy. Specifically, previous studies have reported photoparoxysmal responses in 10.3%–30.5% of patients with juvenile myoclonic epilepsy,^28^, ^29^ 13.0% in generalized tonic–clonic seizures alone, 18.0% in childhood absence epilepsy, and 7.5% in juvenile absence epilepsy.^29^

SEPs are a key neurophysiological tool for assessing pathology in PMEs. In this review, 21 of the 181 included studies (11.6%) reported SEP findings. Among the 49 patients with DRPLA‐related epilepsy who underwent SEP testing, 27 showed no giant SEPs, whereas only two exhibited giant SEPs. These findings align with previous reports suggesting that giant SEPs are typically absent in DRPLA,^30^ in contrast to other PME disorders, in which giant SEPs are frequently observed (85%, 23/27 patients^26^). One possible explanation is that giant SEPs and the C‐reflex reflect cortical hyperexcitability and myoclonus, whereas DRPLA is characterized by degeneration of subcortical structures, as its name implies. The absence of these findings may therefore indicate a different pathophysiological substrate in DRPLA compared to other PMEs. Interestingly, the two patients who exhibited giant SEPs also had SE. This raises the possibility that the presence of giant SEPs in DRPLA may be related to the severity of epileptic manifestations, rather than being a core feature of the disease itself. However, the underlying mechanisms remain unclear.

### 4.5 Management of seizures in patients with DRPLA‐related epilepsy

A meta‐analysis could not be performed for ASM treatment due to the predominance of case reports and small case series, which introduced substantial publication bias. These limitations made it difficult to generate reliable point estimates and 95% CIs for treatment effectiveness, ineffectiveness, and adverse effects. Therefore, instead of a quantitative meta‐analysis, we examined the proportion of studies reporting each treatment outcome, using the number of studies that reported the use of each drug as the denominator and the number of studies reporting effectiveness, ineffectiveness, or adverse effects as numerators (Figure 3).

Interpretation of these findings requires caution, as treatment responses were based on subjective assessments by the original authors and were not adjusted for the number of patients per study. Nonetheless, a general trend was observed; newer generation ASMs such as levetiracetam and perampanel were more frequently reported as effective. Although the precise mechanisms remain unclear, it has been empirically recognized that sodium channel blockers, such as carbamazepine or oxcarbazepine, can exacerbate myoclonus in other types of PME.^31^ A similar trend was noted in this review. In line with treatment approaches for other PMEs, sodium channel blockers may warrant lower priority in the pharmacologic management of DRPLA‐related epilepsy. Despite previous reports suggesting that phenytoin and carbamazepine may exacerbate myoclonic epilepsy,^32^, ^33^, ^34^ our systematic review did not identify a high frequency of such aggravation. Two possible explanations can be considered. First, many of the included studies described treatment histories such as “seizures were not controlled despite the use of multiple antiepileptic drugs including phenytoin and carbamazepine,” making it difficult to disentangle the effect of a single drug from that of polytherapy. Second, aggravation may have been recognized in the clinical course but not explicitly reported. In addition, regarding phenytoin, one report has suggested a potential antiseizure effect in the late stages of PME, which may also account for the findings in our review.^35^ Regarding non‐ASM interventions for seizure control, there was one report each on VNS, dietary therapy, and TRH therapy. Given that each intervention was reported in only a single study, further studies are required to establish their therapeutic value.

### 4.6 Clinical outcomes in patients with DRPLA‐related epilepsy

With respect to hospitalization, 16% of DRPLA patients with epilepsy were reported to have been hospitalized, compared to 4.2% of those without epilepsy, suggesting a somewhat higher frequency of hospitalization among patients with epilepsy. However, as we did not perform a meta‐analysis but rather calculated pooled proportions, these estimates may not accurately reflect the true population rates. A similar methodological limitation applies to the survival analysis; information on (1) age at onset of DRPLA, (2) age at last follow‐up, (3) vital status at last follow‐up, and (4) presence or absence of epilepsy was aggregated across available cases, and Kaplan–Meier analysis with log‐rank testing was performed without accounting for between‐study heterogeneity. Therefore, the resulting survival estimates may not fully represent the underlying population. The analysis indicated a median survival of 19 years in the epilepsy group and 15 years in the nonepilepsy group, with significantly shorter survival observed in patients without epilepsy. When these results are interpreted in conjunction with the meta‐analysis of onset age, it becomes apparent that patients with epilepsy may have a median survival of less than 40 years, whereas those without epilepsy may reach approximately 60 years, suggesting a potential difference of nearly 2 decades in age at death between the groups. Regarding seizure control, only approximately one third of cases with DRPLA‐related epilepsy met strict criteria for drug‐resistant epilepsy, yet approximately three quarters were reported to have uncontrollable seizures, underscoring the difficulty of achieving seizure control in this population. Additionally, although rare (3/386, .8%), cases of SUDEP were also documented.

### 4.7 Methodological considerations

This study has several methodological limitations. First, there was a high degree of heterogeneity across the included studies, limiting the strength of both the meta‐analysis and the systematic review. This heterogeneity likely stems from differences in diagnostic practices since the initial description of DRPLA and its genetic basis.^1^, ^2^, ^27^ Diagnostic approaches have varied across studies, relying on clinical presentation, family history, imaging, or pathology, rather than standardized criteria.^27^ Moreover, the classification of seizures has evolved over time, with multiple revisions by the ILAE since 1982.^36^, ^37^, ^38^, ^39^, ^40^, ^41^ The latest updates in 2017 and 2025 led to inconsistencies in terminology and unclassifiable seizure types in some studies (Methods S3). Second, the meta‐analysis may be affected by publication bias. To reduce this risk, only case series and cohort studies reporting five or more cases were included. Although this threshold is commonly used, there is no consensus on its optimal value. To assess potential selection bias, we performed sensitivity analyses using thresholds of ≥3, ≥4, ≥6, and ≥10 patients. The consistency of results across these thresholds supports the robustness of our findings. Third, we attempted to exclude duplicate cases to the greatest extent possible, removing those explicitly identified as duplicates within the publications. Nevertheless, certain reports described highly similar clinical courses that strongly suggested overlap, yet duplication was not explicitly acknowledged by the authors. Consequently, a limited number of duplicate cases may still have been included in our analysis. Fourth, search bias may have occurred. By focusing on studies specifically addressing epilepsy in DRPLA and comparing patients with and without epilepsy, we may have excluded studies that addressed DRPLA more broadly. Fifth, our search was limited to English and Japanese publications. As a result, potentially relevant studies published in other languages (e.g., German, French, Spanish, Chinese) may have been missed. Sixth, our definition of each outcome depended on each study author's definition or interpretation, especially in seizure characteristics, electrophysiological findings, and the effectiveness of ASMs. This bias might be a plausible explanation for the variability of the patient‐level proportions in the seizure characteristics findings. Especially, myoclonic seizures are often misinterpreted as involuntary myoclonus. In addition, some studies might have classified drop attacks as atonic seizures, as atonic seizures are difficult to diagnose without ictal EEG confirmation. Finally, the rarity of DRPLA meant that many included studies were small case series or individual reports. Although this is a limitation, it is an accepted approach when reviewing rare diseases or narrowly defined clinical populations.^42^, ^43^, ^44^, ^45^ Nonetheless, findings should be interpreted cautiously, particularly regarding their generalizability and clinical implications. Future large‐scale studies could substantially alter the conclusions of this review.

Despite these limitations, this study offers meaningful value by systematically synthesizing the current evidence on DRPLA‐related epilepsy. It addresses the limitations inherent to rare disease research and highlights the need for larger, multicenter cohort studies to refine our understanding and guide clinical care.

## 5 CONCLUSIONS

This systematic review and meta‐analysis summarizes the clinicogenomic background characteristics, clinical manifestations, seizure characteristics, electrophysiological findings, treatment outcome of seizures, and clinical outcomes in patients with DRPLA‐related epilepsy. Patients with epilepsy had an earlier disease onset and a greater number of CAG repeats than those without epilepsy and showed a tendency toward paternal inheritance. In terms of clinical manifestations, cognitive impairment was more prevalent in patients with epilepsy than in those without. In terms of seizure characteristics, focal, clonic, and photosensitive seizures were underrepresented in the overall number of studies but constituted a substantial proportion within the subset of studies that explicitly assessed them. Electrophysiological findings indicated that giant SEPs, which are typically observed in other PMEs, were largely absent in DRPLA, suggesting a different neurophysiological substrate. Regarding the treatment outcome of seizures, newer generation drugs such as levetiracetam and perampanel were more commonly reported as effective, whereas sodium channel blockers were less favorable, consistent with treatment patterns in other PMEs. In terms of seizure control, drug‐resistant epilepsy and uncontrollable seizures were frequent features of DRPLA‐related epilepsy. By comprehensively synthesizing fragmented reports on this rare condition, this study offers an important resource for understanding the clinicogenomic and electrophysiological profile of DRPLA‐related epilepsy. It also highlights the urgent need for prospective, multicenter cohort studies to establish evidence‐based diagnostic and therapeutic strategies for this underrecognized population.

## AUTHOR CONTRIBUTIONS

*Study design:* Takafumi Kubota and Naoto Kuroda. *Data acquisition, screening:* Toru Horinouchi and Haruka Ishibashi. *Full‐text assessment for eligibility:* Toru Horinouchi, Haruka Ishibashi, Yukako Nakagami, Yoko Kobayashi Takahashi, Takato Akiba, Masaharu Miyauchi, Naohiro Yamamoto, Ryoichi Inoue, Satoshi Kodama, Takafumi Kubota, and Naoto Kuroda. *Risk‐of‐bias assessment:* Toru Horinouchi, Haruka Ishibashi, Yukako Nakagami, Yoko Kobayashi Takahashi, Takato Akiba, Masaharu Miyauchi, Naohiro Yamamoto, Ryoichi Inoue, Satoshi Kodama, Takafumi Kubota, and Naoto Kuroda. *Data analysis and synthesis:* Toru Horinouchi, Haruka Ishibashi, Takafumi Kubota, and Naoto Kuroda. *Writing original draft:* Toru Horinouchi, Haruka Ishibashi, Yukako Nakagami, Satoshi Kodama, Takafumi Kubota, and Naoto Kuroda. *Writing review and editing:* Naoto Kuroda. *Supervision:* Satoshi Kodama, Takafumi Kubota, and Naoto Kuroda.

## FUNDING INFORMATION

The authors received no funding for this systematic review and meta‐analysis.

## CONFLICT OF INTEREST STATEMENT

None of the authors has any conflict of interest to disclose. We confirm that we have read the Journal's position on issues involved in ethical publication and affirm that this report is consistent with those guidelines.

## ETHICS STATEMENT

This study did not require ethical approval, as it is a systematic review and meta‐analysis of previously published data. No original data were collected, and no identifiable patient information was used.

## PATIENT CONSENT STATEMENT

Patient consent was not required, as this study is based entirely on published literature and does not involve any individual patient data.

## PROTOCOL REGISTRATION

The protocol of this systematic review and meta‐analysis was registered on the Open Science Framework (https://osf.io/gjdvr/).

## Supporting information


Data S1.

## Data Availability

This study is a systematic review and meta‐analysis based on previously published data. The original datasets analyzed are publicly available in the individual studies cited in the reference list.
