# Supplementary material for: Epilepsy in dentatorubral–pallidoluysian atrophy: A systematic review and meta‐analysis
Source: Epilepsia. 2025 Oct 28;67(2):696–711. doi: 10.1111/epi.18700 (PMC12927676; doi:10.1111/epi.18700)
Supplement: Supplementary file 1 — Data S1. [file EPI-67-696-s001.zip › epi18700-sup-0002-TableS1-S1@DRPLA_Supplementary2_nk12_R_nk3_hi1.docx]

**Supplementary Document 2**

**in**

**Epilepsy in Dentatorubral-Pallidoluysian Atrophy: A Systematic Review and Meta-Analysis**

**It includes**

**Tables S1.**

**The full list of references for all studies included in this systematic review is provided in Supplementary Document 3.**

**Table S1: Summary of study characteristics and findings included in the systematic review and meta-analysis.**

| Author Year | Country | Lan. * | Study† | N of patients　‡ | DRPLA onset § | Sex summary ‖ | CAG repeat ¶ | Inheritance # | Diag. ** | Other reports †† | RoB　‡‡ |
| --- | --- | --- | --- | --- | --- | --- | --- | --- | --- | --- | --- |
| Hasegawa 2010 **S1** | Japan | E | COH | 183; 98; 85 | 31.5; NA; NA | 82; 101; NA; NA; NA; NA | 64.8; NA; NA | NA; NA; NA; NA; NA; NA | G | (-); (-); (-); (-) | 6 |
| Ikiuchi 1995 **S2** | Japan | E | CAS | 65; 23; 42 | 27.2; 15.2; 44.4 | 1; 6; 1; 3; 0; 3 | 65.3; 67.8; 61.8 | 22; 10; 16; 3; 6; 7 | G | (-); (-); (-); (-) | 2 |
| Hirayama 1994 **S3** | Japan | E | CRO | 56; 24; 32 | 31.8; NA; NA | 30; 38; NA; NA; NA; NA | NA; NA; NA | NA; NA; NA; NA; NA; NA | C | (-); (-); (-); (-) | 3 |
| Grimaldi 2019 **S4** | Italia | E | CRO | 51; 24; 10 | 31.4; 24.9; 52.3 | 20; 14; 15; 9; 5; 5 | 61.6; 62.1; 60.2 | 17; 20; 12; 9; 4; 1 | G | (-); (-); (-); (-) | 5 |
| Naito 1982 **S5** | Japan | E | CAS | 44; 21; 23 | 34.8; 22.6; 54.0 | 19; 25; 8; 13; 11; 12 | NA; NA; NA | 32; 8; 17; 4; 15; 4 | P | (+); (+); (-); (-) | 2 |
| Komure 1995 **S6** | Japan | E | CRO | 38; 18; 20 | NA; 7.9; 43.6 | NA; NA; NA; NA; NA; NA | 62.8; NA; NA | 27; 9; 17; 1; 10; 8 | G | (-); (-); (-); (-) | 6 |
| Vale 2010 **S7** | Portugal | E | CAS | 25; 12; 13 | 29.6; NA; NA | NA; NA; NA; NA; NA; NA | NA; NA; NA | NA; NA; NA; NA; NA; NA | G | (-); (-); (-); (-) | 6 |
| Onuma 2000 **S8** | Japan | J | CRO | 25; NA; NA | NA; NA; NA | NA; NA; NA; NA; NA; NA | NA; NA; NA | NA; NA; NA; NA; NA; NA | C | (-); (-); (+); (-) | 0 |
| Sano 1994 **S9** | Japan | E | CAS | 23; 12; 11 | 32.7; 17.7; 44.1 | 9; 14; 4; 8; 5; 6 | NA; NA; NA | 12; 4; 11; 1; 1; 3 | P | (-); (-); (-); (-) | 3 |
| Koide 1994 **S10** | Japan | E | CAC | 22; 10; 12 | 30.2; 13.0; 47.3 | NA; NA; NA; NA; NA; NA | 63.8; 66.1; 61.4 | NA; NA; 10; 0; NA; NA | G | (-); (-); (-); (-) | 4 |
| Baba 1987 **S11** | Japan | J | CAS | 22; 16; 6 | 26.1; 18.3; 47.2 | 8; 14; 5; 11; 3; 3 | NA; NA; NA | NA; NA; NA; NA; NA; NA | P, C | (-); (+); (-); (-) | 5 |
| Yamaguchi 2020 **S12** | Japan | E | CAS | 20; NA; NA | NA; NA; NA | NA; NA; NA; NA; NA; NA | NA; NA; NA | NA; NA; NA; NA; NA; NA | C | (-); (-); (-); (-) | 2 |
| Warner 1995 **S13** | UK | E | CAS | 19; 14; 5 | 30.1; 22.6; 49.6 | 11; 8; 8; 6; 3; 2 | 64.0; NA; NA | 8; 8; 8; 5; 0; 3 | G | (+); (+); (-); (+) | 3 |
| Contesse 2024 **S14** | USA and UK | E | CAS | 18; 11; 7 | 15.0; NA; NA | 12; 6; NA; NA; NA; NA | NA; NA; NA | NA; NA; NA; NA; NA; NA | NA | (+); (-); (-); (-) | 4 |
| Egawa 2008 **S15** | Japan | E | CAS | 17; 17; 0 | 7.1; 7.1; NA | 9; 8; 9; 8; 0; 0 | 69.9; 69.9; NA | NA; NA; NA; NA; NA; NA | G | (+); (+); (-); (+) | 4 |
| Iwabuchi 1993 **S16** | Japan | J | CRO | 17; 13; 4 | 29.2; 22.8; 50.0 | 4; 13; 3; 10; 1; 3 | NA; NA; NA | NA; NA; NA; NA; NA; NA | P | (+); (+); (-); (-) | 3 |
| Nielsen 1996 **S17** | Denmark | E | CAS | 15; 6; 9 | 36.7; 28.0; 42.1 | 6; 9; 1; 5; 5; 4 | 61.4; 64.7; 59.8 | 4; 11; 2; 4; 2; 7 | G | (+); (+); (-); (+) | 4 |
| Wardle 2008 **S18** | UK | E | CAS | 15; 10; 5 | 35.8; 37.3; 32.7 | 11; 4; 7; 3; 4; 1 | 58.8; 57.8; 58.7 | 4; 5; 3; 3; 1; 2 | G | (+); (-); (-); (-) | 4 |
| Hayashi 2007 **S19** | Japan | E | CAC | 14; 9; 5 | NA; NA; NA | 9; 5; 6; 3; 3; 2 | 67.3; 69.3; 65.3 | 2; 2; 2; 1; 0; 1 | P | (+); (-); (-); (-) | 6 |
| Iwabuchi 1987B **S20** | Japan | J | CAS | 13; 9; 4 | 28.8; 21.7; 45.0 | 6; 7; 5; 4; 1; 3 | NA; NA; NA | NA; NA; NA; NA; NA; NA | P, C | (+); (+); (-); (-) | 2 |
| Kasai 1999 **S21** | Japan | E | CAC | 12; 11; 1 | 20.0; 17.0; 53.0 | 7; 5; 6; 5; 1; 0 | 65.7; 66.7; 56.0 | NA; NA; NA; NA; NA; NA | G | (-); (-); (+); (-) | 6 |
| Tomoda 1991 **S22** | Japan | E | CAR | 12; 4; 8 | 23.3; 7.5; 31.3 | 9; 3; 3; 1; 6; 2 | NA; NA; NA | 8; 0; 4; 0; 4; 0 | C | (+); (+); (+); (+) | 6 |
| Norremolle 1995 **S23** | Denmark | E | CAS | 12; 7; 5 | 30.7; 26.0; 40.0 | 6; 6; 2; 5; 4; 1 | 64.2; 66.0; 63.0 | 5; 6; 4; 3; 1; 3 | G | (+); (+); (-); (-) | 4 |
| Takeda 1996 **S24** | Japan | E | CAS | 12; 10; 2 | 27.7; 20.9; 61.5 | 4; 8; 4; 6; 0; 2 | NA; NA; NA | NA; NA; NA; NA; NA; NA | P | (-); (-); (-); (-) | 4 |
| Muñoz 1999 **S25** | Spain | E | CAS | 12; 4; 8 | 27.7; 12.8; 37.7 | 0; 2; 0; 1; 0; 1 | 60.0; NA; 60.0 | NA; NA; NA; NA; NA; NA | P | (+); (-); (-); (-) | 2 |
| Takiguchi 1992 **S26** | Japan | J | CAS | 11; 3; 8 | 35.0; 20.7; 45.8 | 6; 5; 3; 0; 3; 5 | NA; NA; NA | 4; 2; 2; 1; 2; 1 | C | (+); (+); (-); (+) | 2 |
| Naito 1972 **S27** | Japan | J | CAS | 11; 5; 6 | 31.8; 19.6; 44.0 | 4; 7; 2; 3; 2; 4 | NA; NA; NA | 8; 3; 3; 2; 5; 1 | C | (+); (+); (-); (+) | 4 |
| Touge 2000 **S28** | Japan | E | CAC | 10; 5; 5 | 31.9; 21.2; 42.6 | NA; NA; NA; NA; NA; NA | 62.6; 63.4; 61.8 | NA; NA; NA; NA; NA; NA | G | (-); (+); (+); (-) | 7 |
| Miyata 2008 **S29** | Japan | E | CAC | 10; 6; 4 | NA; NA; NA | 7; 3; 5; 1; 2; 2 | 66.4; 68.0; 65.3 | 2; 2; 2; 1; 0; 1 | G, P | (+); (-); (-); (-) | 6 |
| Becher 1997 **S30** | US, UK | E | CAS | 10; 5; 2 | 30.1; 19.4; 45.5 | 6; 4; 3; 2; 1; 1 | 64.3; 70.3; 57.0 | 4; 3; 3; 0; 1; 1 | G | (+); (+); (-); (+) | 2 |
| Tokiguchi 1987 **S31** | Japan | J | CAS | 10; 4; 6 | 21.4; 14.0; 27.4 | 4; 6; 1; 3; 3; 3 | NA; NA; NA | 5; 4; 3; 1; 2; 3 | C | (-); (-); (-); (-) | 3 |
| Kasai 1998 **S32** | Japan | J | CRO | 10; 10; 0 | 17.2; 17.2; NA | 5; 5; 5; 5; 0; 0 | 67.2; 67.2; NA | NA; NA; NA; NA; NA; NA | G | (-); (-); (+); (-) | 4 |
| Maruyama 2012 **S33** | Japan | E | COH | 9; 9; 0 | 3.9; 3.9; NA | 6; 3; 6; 3; 0; 0 | 78.2; 78.2; NA | 7; 1; 7; 1; 0; 0 | G | (-); (-); (-); (-) | 8 |
| Mizoi 1994 **S34** | Japan | J | CAC | 8; 3; 5 | 31.9; 27.3; 42.6 | 5; 3; 3; 0; 3; 2 | NA; NA; NA | 1; 0; 1; 0; 0; 0 | P, C | (-); (+); (-); (-) | 3 |
| Pinto 2021 **S35** | Brazil | E | CAS | 8; 2; 6 | 42.8; 11.5; 53.2 | 5; 3; 2; 0; 3; 3 | 53.0; 70.0; 50.2 | 3; 4; 2; 0; 1; 4 | G | (+); (-); (-); (-) | 6 |
| Tsuchiya 1995 **S36** | Japan | E | CAS | 8; 7; 1 | 24.5; 21.9; 43.0 | 5; 3; 5; 2; 0; 1 | NA; NA; NA | 2; 1; 2; 1; 0; 0 | P | (-); (-); (-); (-) | 6 |
| Oda 2015 **S37** | Japan | J | CAS | 8; 8; 0 | 6.7; 6.7; NA | NA; NA; NA; NA; NA; NA | 70.8; 70.8; NA | 7; 0; 7; 0; NA; NA | G, C | (+); (-); (-); (+) | 4 |
| Tsuchiya 1998 **S38** | Japan | E | CAC | 7; 6; 1 | 29.0; 26.7; 43.0 | 4; 3; 4; 2; 0; 1 | NA; NA; NA | 1; 0; 1; 0; 0; 0 | P | (-); (-); (-); (-) | 1 |
| Hayashi 2012 **S39** | Japan | E | CAC | 7; 7; 0 | NA; NA; NA | 5; 2; 5; 2; NA; NA | NA; NA; NA | NA; NA; NA; NA; NA; NA | G | (-); (-); (-); (-) | 3 |
| Licht 2002 **S40** | North America | E | CAS | 7; 4; 3 | 12.4; 8.8; 23.0 | 5; 2; 3; 1; 2; 1 | 73.5; 73.5; NA | 1; 3; 1; 3; 0; 0 | G | (+); (+); (-); (+) | 1 |
| Zhang 2020 **S41** | China | E | CAS | 7; 7; 0 | 9.6; 9.6; NA | 6; 1; 4; 0; 2; 1 | 70.0; 71.5; 68.5 | NA; NA; NA; NA; NA; NA | G | (+); (-); (-); (-) | 3 |
| Aoki 1994 **S42** | Japan | E | CAS | 6; 3; 3 | 29.3; 11.3; 47.3 | 2; 2; 0; 1; 2; 1 | 64.5; 66.5; 62.5 | 1; 3; 0; 3; 1; 0 | G | (+); (-); (-); (-) | 2 |
| Uyama 1995 **S43** | Japan | E | CAS | 6; 2; 4 | 36.8; 6.8; 51.8 | 1; 5; 0; 2; 1; 3 | 67.8; 75.5; 64.0 | 1; 3; 1; 1; 0; 2 | G | (+); (-); (-); (-) | 3 |
| Kishimoto 1995 **S44** | Japan | E | CAS | 6; 4; 2 | 20.7; 11.3; 38.0 | 3; 3; 3; 1; 4; 0 | NA; NA; NA | 4; 2; 4; 0; 0; 2 | G | (+); (+); (-); (-) | 0 |
| Asai 1997B **S45** | Japan | J | CAC | 5; 5; 0 | 16.4; 16.4; NA | 2; 3; 2; 3; 0; 0 | 67.8; 67.8; NA | 0; 4; 0; 4; 0; 0 | G | (+); (+); (-); (-) | 4 |
| Saito 1998 **S46** | Japan | E | CAS | 5; 2; 3 | 17.0; 5.0; 35.0 | 4; 1; 2; 1; 2; 0 | NA; NA; NA | 5; 0; 3; 0; 2; 0 | G | (+); (+); (-); (+) | 3 |
| Yam 2004 **S47** | China | E | CAS | 5; 4; 1 | 11.8; 4.3; 42.0 | 3; 2; 2; 2; 1; 0 | 69.2; 70.0; 66.0 | 2; 0; 2; 0; 0; 0 | G | (+); (+); (+); (-) | 5 |
| Nakano 2012 **S48** | Japan | E | CAS | 5; 5; 0 | 13.6; 13.6; NA | NA; NA; NA; NA; NA; NA | NA; NA; NA | NA; NA; NA; NA; NA; NA | G | (+); (+); (-); (-) | 3 |
| Kim 2018 **S49** | Korea | E | CAS | 5; 3; 2 | 23.2; 10.0; 43.0 | 1; 4; 0; 3; 1; 1 | 60.0; 61.7; 57.5 | 5; 0; 3; 0; 2; 0 | G | (-); (-); (-); (-) | 5 |
| Takahashi 1978 **S50** | Japan | E | CAS | 5; 4; 1 | 17.4; 8.3; 54.0 | 2; 3; 1; 3; 1; 0 | NA; NA; NA | 2; 3; 2; 2; 0; 1 | P | (+); (+); (-); (-) | 5 |
| Honzawa 2012 **S51** | Japan | J | CAS | 5; 5; 0 | 6.0; 6.0; NA | 4; 1; 4; 1; 0; 0 | NA; NA; NA | 1; 0; 1; 0; 0; 0 | G | (+); (-); (-); (-) | 6 |
| Toyoshima 2020 **S52** | Japan | J | CAS | 5; 4; 1 | NA; NA; NA | 3; 2; 2; 2; 1; 0 | NA; NA; NA | NA; NA; NA; NA; NA; NA | C | (-); (-); (-); (+) | 1 |
| Takahashi 1999 **S53** | Japan | E | CAC | 4; 4; 0 | NA; NA; NA | NA; NA; NA; NA; NA; NA | NA; NA; NA | NA; NA; NA; NA; NA; NA | C | (-); (+); (-); (-) | 2 |
| Sone 2016 **S54** | Japan | E | CAC | 4; 4; 0 | 15.5; 15.5; NA | 1; 3; 1; 3; NA; NA | 71.8; 71.8; NA | NA; NA; NA; NA; NA; NA | G | (+); (+); (-); (-) | 4 |
| Warner 1994 **S55** | UK | E | CAS | 4; 4; 0 | 23.5; 23.5; NA | 1; 3; 1; 3; 0; 0 | NA; NA; NA | 0; 4; 0; 4; NA; NA | P, C | (+); (+); (-); (+) | 1 |
| Potter 1995 **S56** | USA | E | CAS | 4; 4; 0 | 29.8; 29.8; NA | 3; 1; 3; 1; NA; NA | 63.5; 63.5; NA | 2; 2; 2; 2; NA; NA | G | (+); (-); (-); (-) | 3 |
| Nakayama 1997 **S57** | Japan | E | CAS | 4; 4; 0 | 8.0; 8.0; NA | 0; 4; 0; 4; 0; 0 | 69.7; 69.7; NA | NA; NA; NA; NA; NA; NA | C | (-); (+); (-); (-) | 3 |
| Iwabuchi 1987A **S58** | Japan | J | CAS | 4; 3; 1 | 17.5; 9.3; 42.0 | 3; 1; 2; 1; 1; 0 | NA; NA; NA | 3; 0; 3; 0; 0; 0 | P | (+); (+); (-); (+) | 3 |
| Tamagaki 1996 **S59** | Japan | J | CAS | 4; 4; 0 | 25.0; 25.0; NA | 2; 2; 2; 2; NA; NA | 68.0; 68.0; NA | 0; 4; 0; 4; NA; NA | G | (+); (+); (-); (-) | 4 |
| Otsuka 1996 **S60** | Japan | J | CAS | 4; 3; 1 | 14.8; 7.0; 38.0 | 3; 1; 2; 1; 1; 0 | 73.0; 75.7; 65.0 | 4; 0; 3; 0; 1; 0 | G | (+); (-); (-); (-) | 4 |
| Saito 1997 **S61** | Japan | J | CAS | 4; 4; 0 | 17.8; 17.8; NA | 2; 2; 2; 2; 0; 0 | 64.0; 64.0; NA | 2; 2; 2; 2; 0; 0 | G | (+); (+); (-); (+) | 6 |
| Sato 1999 **S62** | Japan | J | CAS | 4; 4; 0 | 44.7; 44.7; NA | 2; 2; 2; 2; 0; 0 | 66.5; 66.5; NA | 1; 2; 1; 2; 0; 0 | G | (+); (+); (-); (+) | 3 |
| Imamura 2007 **S63** | Japan | J | CAS | 4; 3; 1 | 28.3; 14.0; 71.0 | 4; 0; 3; 0; 1; 0 | 69.0; 69.0; NA | 3; 0; 3; 0; 0; 0 | G | (+); (+); (-); (+) | 3 |
| Koyanagi 1977 **S64** | Japan | J | CAS | 4; 4; 0 | 17.3; 17.3; NA | 2; 2; 2; 2; 0; 0 | NA; NA; NA | 2; 0; 2; 0; 0; 0 | P | (+); (+); (-); (-) | 4 |
| Segawa 1991 **S65** | Japan | J | CAC | 3; 2; 1 | 36.7; 32.5; 45.0 | NA; NA; NA; NA; NA; NA | NA; NA; NA | 1; 0; 1; 0; 0; 0 | C | (-); (-); (+); (-) | 3 |
| Asai 1997A **S66** | Japan | J | CAC | 3; 3; 0 | 14.0; 14.0; NA | 2; 1; 2; 1; 0; 0 | 70.7; 70.7; NA | 0; 3; 0; 3; 0; 0 | G | (+); (+); (-); (-) | 4 |
| Takahashi 1988 **S67** | Japan | E | CAR | 3; 1; 2 | 38.3; 8.0; 53.5 | 2; 1; 1; 0; 1; 1 | NA; NA; NA | 3; 0; 1; 0; 2; 0 | C | (+); (+); (-); (+) | 0 |
| Mizukami 1995 **S68** | Japan | E | CAR | 3; 2; 1 | 33.0; 25.0; 49.0 | 2; 1; 1; 1; 1; 0 | 63.0; 63.0; 63.0 | NA; NA; NA; NA; NA; NA | G | (+); (+); (-); (+) | 5 |
| Ber 2003 **S69** | France | E | CAR | 3; 3; 0 | 21.3; 21.3; NA | 1; 2; 1; 2; 0; 0 | 63.3; 63.3; NA | 1; 0; 1; 0; 0; 0 | G | (+); (-); (-); (-) | 3 |
| Vinton 2005 **S70** | Austraria | E | CAR | 3; 1; 2 | 34.7; 5.0; 49.5 | 3; 0; 1; 0; 2; 0 | 58.3; 66.0; 54.5 | 2; 0; 1; 0; 1; 0 | G | (+); (+); (-); (+) | 7 |
| Wu 2010 **S71** | Australia | E | CAR | 3; 2; 1 | 19.3; 4.5; 49.0 | 2; 1; 1; 1; 1; 0 | 64.7; 68.0; 58.0 | 2; 0; 2; 0; 0; 0 | G | (-); (+); (-); (-) | 2 |
| Miguel 2014 **S72** | Portugal | E | CAR | 3; 2; 1 | 33.3; 18.0; 64.0 | 1; 2; 0; 2; 1; 0 | 63.0; 63.0; NA | 1; 0; 1; 0; 0; 0 | G | (+); (+); (-); (-) | 1 |
| Kuts 2019 **S73** | USA | E | CAR | 3; 3; 0 | 15.0; 15.0; NA | 2; 1; 2; 1; 0; 0 | 67.0; 67.0; NA | 2; 0; 2; 0; 0; 0 | G | (+); (-); (-); (+) | 3 |
| Amprosi 2021 **S74** | Austria | E | CAR | 3; 3; 0 | 28.0; 28.0; NA | 1; 2; 1; 2; 0; 0 | 63.0; 63.0; NA | 1; 1; 1; 1; 0; 0 | G | (+); (-); (-); (-) | 5 |
| Arai 1995 **S75** | Japan | E | CAR | 3; 2; 1 | 38.0; 31.0; 52.0 | 3; 0; 2; 0; 1; 0 | NA; NA; NA | NA; NA; NA; NA; NA; NA | P | (-); (-); (-); (-) | 5 |
| Miyazaki 1996 **S76** | Japan | E | CAR | 3; 3; 0 | 6.3; 6.3; NA | 2; 1; 2; 1; 0; 0 | 75.7; 75.7; NA | 2; 1; 2; 1; 0; 0 | G | (-); (+); (+); (-) | 3 |
| Takahashi 1997 **S77** | Japan | E | CAR | 3; 3; 0 | 4.8; 4.8; NA | 1; 2; 1; 2; 0; 0 | 72.3; 72.3; NA | 1; 0; 1; 0; 0; 0 | G | (+); (+); (-); (-) | 5 |
| Harisankar 2025 **S78** | India | E | CAR | 3; 3; 0 | 27.0; 27.0; NA | 1; 2; 1; 2; 0; 0 | 60.0; 60.0; NA | 3; 0; 3; 0; NA; NA | G | (+); (+); (-); (+) | 5 |
| Tokuda 1990 **S79** | Japan | J | CAR | 3; 2; 1 | 37.7; 25.5; 62.0 | 0; 3; 0; 2; 0; 1 | NA; NA; NA | 0; 2; 0; 2; 0; 0 | C | (+); (+); (-); (+) | 6 |
| Kawakatsu 1991 **S80** | Japan | J | CAR | 3; 3; 0 | 42.0; 42.0; NA | 2; 1; 2; 1; 0; 0 | NA; NA; NA | 3; 0; 3; 0; 0; 0 | P | (+); (+); (-); (-) | 4 |
| Miyanaga 1995 **S81** | Japan | J | CAR | 3; 2; 1 | 40.0; 36.5; 47.0 | 1; 2; 1; 1; 0; 1 | NA; NA; NA | NA; NA; NA; NA; NA; NA | G | (-); (+); (-); (-) | 5 |
| Nakamura 1995 **S82** | Japan | J | CAR | 3; 2; 1 | 30.3; 22.5; 46.0 | 2; 1; 2; 0; 0; 1 | 63.7; 66.0; 59.0 | 0; 2; 0; 2; 0; 0 | G | (+); (+); (-); (-) | 5 |
| Iyoda 1997 **S83** | Japan | J | CAR | 3; 3; 0 | 6.3; 6.3; NA | 2; 1; 2; 1; 0; 0 | 76.7; 76.7; NA | 3; 0; 3; 0; 0; 0 | G | (+); (+); (-); (+) | 7 |
| Fujii 1997 **S84** | Japan | J | CAR | 3; 2; 1 | 20.3; 12.0; 37.0 | 1; 2; 1; 1; 0; 1 | 63.0; 64.0; 61.0 | 0; 2; 0; 2; 0; 0 | G | (+); (-); (-); (+) | 6 |
| Takahara 1997 **S85** | Japan | J | CAR | 3; 1; 2 | 33.7; 17.0; 42.0 | 1; 2; 0; 1; 1; 1 | 62.5; 62.0; 63.0 | 1; 2; 0; 1; 1; 1 | G | (-); (+); (-); (-) | 4 |
| Murata 1997 **S86** | Japan | J | CAR | 3; 1; 2 | 37.0; 19.0; 46.0 | 0; 3; 0; 1; 0; 2 | 60.0; 61.0; 59.5 | 0; 2; 0; 1; 0; 1 | G | (+); (+); (-); (-) | 4 |
| Ishino 1973 **S87** | Japan | J | CAR | 3; 3; 0 | 2.5; 2.5; NA | 0; 3; 0; 3; 0; 0 | NA; NA; NA | NA; NA; NA; NA; NA; NA | P | (+); (+); (-); (+) | 7 |
| Sun 2023 **S88** | China | E | CAS | 3; 2; 1 | 5.5; 5.5; NA | 0; 3; 0; 2; 0; 1 | 62.7; 69.0; 50.0 | 2; NA; 2; 0; NA; NA | G | (-); (-); (-); (-) | 3 |
| Miyashita 1992 **S89** | Japan | J | CAS | 3; 1; 2 | 38.3; 14.0; 50.5 | 2; 1; 1; 0; 0; 2 | NA; NA; NA | 1; 1; 1; 0; 0; 1 | P | (+); (+); (-); (-) | 3 |
| Miiyazaki 1995 **S90** | Japan | E | CAR | 2; 2; 0 | 28.5; 28.5; NA | 2; 0; 2; 0; 0; 0 | 76.0; 76.0; NA | 1; 0; 1; 0; 0; 0 | G | (+); (-); (-); (-) | 1 |
| Hattori 1997 **S91** | Japan | E | CAR | 2; 2; 0 | 2.8; 2.8; NA | 2; 0; 2; 0; 0; 0 | 73.0; 73.0; NA | 2; 0; 2; 0; 0; 0 | G | (+); (+); (-); (+) | 7 |
| Lee 2001 **S92** | China | E | CAR | 2; 2; 0 | 44.0; 44.0; NA | 2; 0; 2; 0; 0; 0 | 60.5; 60.5; NA | 1; 0; 1; 0; 0; 0 | G | (+); (-); (-); (-) | 5 |
| Casseron 2004 **S93** | France | E | CAR | 2; 2; 0 | 18.0; 18.0; NA | 0; 2; 0; 2; 0; 0 | 64.5; 64.5; NA | 2; 0; 2; 0; 0; 0 | G | (+); (+); (-); (-) | 2 |
| Morita 2007 **S94** | Japan | E | CAR | 2; 2; 0 | 12.0; 12.0; NA | 1; 1; 1; 1; 0; 0 | NA; NA; NA | 1; 1; 1; 1; 0; 0 | G | (-); (-); (-); (+) | 6 |
| Sunami 2011 **S95** | Japan | E | CAR | 2; 1; 1 | 37.5; 19.0; 56.0 | 0; 2; 0; 1; 0; 1 | 67.5; 70.0; 65.0 | 1; 0; 1; 0; 0; 0 | G | (+); (-); (-); (-) | 1 |
| Isobe 2016 **S96** | Japan | E | CAR | 2; 2; 0 | 5.0; 5.0; NA | 1; 1; 1; 1; 0; 0 | 74.0; 74.0; NA | NA; NA; NA; NA; NA; NA | G | (+); (+); (-); (+) | 6 |
| Zook-Lewis 2019 **S97** | USA | E | CAR | 2; 2; 0 | NA; NA; NA | NA; NA; NA; NA; NA; NA | NA; NA; NA | NA; NA; NA; NA; NA; NA | C | (+); (-); (-); (+) | 4 |
| Alshimemeri 2020 **S98** | Canada | E | CAR | 2; 2; 0 | 35.0; 35.0; NA | 1; 1; 1; 1; 0; 0 | NA; NA; NA | NA; NA; NA; NA; NA; NA | P | (+); (-); (-); (-) | 2 |
| Iwabuchi 1985 **S99** | Japan | J | CAR | 2; 1; 1 | 45.5; 33.0; 58.0 | 0; 2; 0; 1; 0; 1 | NA; NA; NA | 1; 1; 1; 0; 0; 1 | P | (+); (+); (-); (+) | 6 |
| Akashi 1987 **S100** | Japan | J | CAR | 2; 2; 0 | 21.5; 21.5; NA | 2; 0; 2; 0; 0; 0 | NA; NA; NA | 1; 1; 1; 1; 0; 0 | C | (+); (+); (-); (-) | 5 |
| Sakurai 1990 **S101** | Japan | J | CAR | 2; 1; 1 | 33.5; 16.0; 51.0 | 1; 1; 0; 1; 1; 0 | NA; NA; NA | NA; NA; NA; NA; NA; NA | C | (+); (+); (+); (-) | 2 |
| Sasaki 1995 **S102** | Japan | J | CAR | 2; 2; 0 | 4.0; 4.0; NA | 0; 2; 0; 2; NA; NA | NA; NA; NA | NA; NA; NA; NA; NA; NA | G | (+); (+); (+); (-) | 4 |
| Yamashita 1995 **S103** | Japan | J | CAR | 2; 2; 0 | 3.0; 3.0; NA | 0; 2; 0; 2; 0; 0 | 70.0; 70.0; NA | NA; NA; NA; NA; NA; NA | G | (+); (+); (+); (+) | 5 |
| Sasagawa 1997 **S104** | Japan | J | CAR | 2; 2; 0 | 13.0; 13.0; NA | 0; 2; 0; 2; 0; 0 | 67.0; 67.0; NA | 1; 0; 1; 0; 0; 0 | G | (+); (+); (+); (-) | 3 |
| Nishio 1998 **S105** | Japan | J | CAR | 2; 1; 0 | 28.5; 17.0; 40.0 | 2; 0; 1; 0; 1; 0 | 58.5; 61.0; 56.0 | 1; 0; 1; 0; 0; 0 | G | (+); (+); (-); (-) | 4 |
| Sugie 1998 **S106** | Japan | J | CAR | 2; 2; 0 | 27.5; 27.5; NA | 1; 1; 1; 1; 0; 0 | 60.0; 60.0; NA | 0; 2; 0; 2; 0; 0 | G | (+); (+); (-); (-) | 5 |
| Shimode 2000 **S107** | Japan | J | CAR | 2; 1; 1 | 26.5; 14.0; 39.0 | 0; 2; 0; 2; 0; 0 | 63.0; 66.0; 60.0 | 1; 1; 0; 1; 1; 0 | G | (+); (+); (-); (-) | 4 |
| Miyazaki 2000 **S108** | Japan | J | CAR | 2; 2; 0 | 25.5; 25.5; NA | 1; 1; 1; 1; 0; 0 | 67.0; 67.0; NA | 2; 0; 2; 0; 0; 0 | G | (+); (+); (+); (+) | 4 |
| Hashi 2007 **S109** | Japan | J | CAR | 2; 2; 0 | 0.6; 0.6; NA | 0; 2; 0; 2; 0; 0 | 82.5; 82.5; NA | 2; 0; 2; 0; 0; 0 | G | (+); (+); (-); (+) | 8 |
| Naito 1977 **S110** | Japan | J | CAR | 2; 2; 0 | 23.0; 23.0; NA | 1; 1; 1; 1; 0; 0 | NA; NA; NA | 0; 1; 0; 1; 0; 0 | P | (+); (+); (-); (-) | 3 |
| Miyahara 2009 **S111** | Japan | E | CAS | 2; 2; 0 | 5.5; 5.5; NA | 2; 0; 2; 0; 0; 0 | NA; NA; NA | NA; NA; NA; NA; NA; NA | C | (+); (+); (+); (+) | 3 |
| Oi 2021 **S112** | Japan | E | CAS | 2; 2; 0 | NA; NA; NA | 2; 0; 2; 0; 0; 0 | NA; NA; NA | NA; NA; NA; NA; NA; NA | G | (-); (-); (+); (+) | 7 |
| Qu 2022 **S113** | China | E | COH | 2; 2; 0 | NA; NA; NA | NA; NA; NA; NA; NA; NA | NA; NA; NA | NA; NA; NA; NA; NA; NA | G | (+); (-); (-); (+) | 2 |
| Suzuki 1985 **S114** | Japan | E | CAR | 1; 1; 0 | 8.0; 8.0; NA | 1; 0; 1; 0; 0; 0 | NA; NA; NA | NA; NA; NA; NA; NA; NA | P | (+); (-); (-); (-) | 5 |
| Pfeiffer 1990 **S115** | USA | E | CAR | 1; 1; 0 | 18.0; 18.0; NA | 1; 0; 1; 0; 0; 0 | NA; NA; NA | NA; NA; NA; NA; NA; NA | P | (+); (+); (-); (-) | 4 |
| Imamura 1994B **S116** | Japan | E | CAR | 1; 1; 0 | 2.0; 2.0; NA | 1; 0; 1; 0; 0; 0 | 76.0; 76.0; NA | 1; 0; 1; 0; NA; NA | G | (+); (+); (-); (+) | 6 |
| Imamura 1994C **S117** | Japan | E | CAR | 1; 1; 0 | 2.0; 2.0; NA | 1; 0; 1; 0; 0; 0 | NA; NA; NA | 1; 0; 1; 0; 0; 0 | C | (+); (+); (+); (+) | 3 |
| Natsue 1996 **S118** | Japan | E | CAR | 1; 1; 0 | 27.0; 27.0; NA | 0; 1; 0; 1; 0; 0 | 64.0; 64.0; NA | 1; 0; 1; 0; 0; 0 | G | (+); (+); (-); (-) | 5 |
| Villani 1998 **S119** | Italy | E | CAR | 1; 1; 0 | 6.0; 6.0; NA | 0; 1; 0; 1; 0; 0 | 67.0; 67.0; NA | NA; NA; NA; NA; NA; NA | G | (+); (+); (+); (+) | 6 |
| Cox 2000 **S120** | UK | E | CAR | 1; 1; 0 | 5.0; 5.0; NA | 1; 0; 1; 0; 0; 0 | 70.0; 70.0; NA | 1; 0; 1; 0; 0; 0 | G | (+); (+); (-); (+) | 7 |
| Takano 2003 **S121** | Japan | E | CAR | 1; 1; 0 | 2.6; 2.6; NA | 0; 1; 0; 1; 0; 0 | 61.0; 61.0; NA | NA; NA; NA; NA; NA; NA | G | (+); (+); (-); (-) | 4 |
| Watarai 2003 **S122** | Japan | E | CAR | 1; 1; 0 | 19.0; 19.0; NA | 0; 1; 0; 1; 0; 0 | 65.0; 65.0; NA | NA; NA; NA; NA; NA; NA | G | (+); (+); (-); (-) | 7 |
| Jung 2004 **S123** | Korea | E | CAR | 1; 1; 0 | 20.0; 20.0; NA | 1; 0; 1; 0; 0; 0 | 71.0; 71.0; NA | 1; 0; 1; 0; NA; NA | G | (+); (-); (-); (-) | 4 |
| Brunetti-Pierri 2006 **S124** | US | E | CAR | 1; 1; 0 | 0.2; 0.2; NA | 1; 0; 1; 0; 0; 0 | 66.0; 66.0; NA | 1; 0; 1; 0; 0; 0 | G | (+); (+); (-); (+) | 7 |
| Kobayashi 2006 **S125** | Japan | E | CAR | 1; 1; 0 | 5.0; 5.0; NA | 1; 0; 1; 0; 0; 0 | 72.0; 72.0; NA | 1; 0; 1; 0; NA; NA | G | (+); (+); (-); (+) | 5 |
| Takemura 2006 **S126** | Japan | E | CAR | 1; 1; 0 | 15.0; 15.0; NA | 0; 1; 0; 1; 0; 0 | 69.0; 69.0; NA | NA; NA; NA; NA; NA; NA | G | (+); (+); (-); (-) | 6 |
| Yiş 2009 **S12**7 | Turkey | E | CAR | 1; 1; 0 | 9.0; 9.0; NA | 0; 1; 0; 1; 0; 0 | 73.0; 73.0; NA | 1; 0; 1; 0; 0; 0 | G | (+); (+); (-); (-) | 4 |
| Sudo 2010 **S128** | Japan | E | CAR | 1; 1; 0 | 6.0; 6.0; NA | 0; 1; 0; 1; 0; 0 | NA; NA; NA | 1; 0; 1; 0; 0; 0 | G | (+); (+); (-); (+) | 5 |
| Simpson 2012 **S129** | New Zealand | E | CAR | 1; 1; 0 | 23.0; 23.0; NA | 1; 0; 1; 0; 0; 0 | 61.0; 61.0; NA | 1; 0; 1; 0; 0; 0 | G | (+); (-); (-); (-) | 4 |
| Saito 2013 **S130** | Japanese | E | CAR | 1; 1; 0 | 12.0; 12.0; NA | 0; 1; 0; 1; 0; 1 | 62.0; 62.0; NA | NA; NA; NA; NA; NA; NA | G | (+); (-); (-); (-) | 2 |
| Zadori 2014 **S131** | Hungary | E | CAR | 1; 1; 0 | NA; NA; NA | 0; 1; 0; 1; 0; 0 | NA; NA; NA | NA; NA; NA; NA; NA; NA | G | (-); (+); (-); (-) | 2 |
| Nam 2015 **S132** | Korea | E | CAR | 1; 1; 0 | 28.0; 28.0; NA | 1; 0; 1; 0; 0; 0 | 59.0; 59.0; NA | NA; NA; NA; NA; NA; NA | G | (-); (-); (-); (-) | 5 |
| Souza 2016 **S133** | Brazil | E | CAR | 1; 1; 0 | 17.0; 17.0; NA | 1; 0; 1; 0; 0; 0 | 70.0; 70.0; NA | 1; 0; 1; 0; 0; 0 | G | (+); (-); (-); (-) | 3 |
| Shiraishi 2017 **S134** | Japan | E | CAR | 1; 1; 0 | 1.0; 1.0; NA | 1; 0; 1; 0; 0; 0 | 70.0; 70.0; NA | 1; 0; 1; 0; 0; 0 | G | (+); (+); (-); (+) | 8 |
| Narita 2017 **S135** | Japan | E | CAR | 1; 1; 0 | 37.0; 37.0; NA | 1; 0; 1; 0; 0; 0 | 59.0; 59.0; NA | 1; 0; 1; 0; 0; 0 | G | (-); (-); (-); (+) | 8 |
| Nandanwar 2018 **S136** | USA | E | CAR | 1; 1; 0 | 27.0; 27.0; NA | 0; 1; 0; 1; 0; 0 | 64.0; 64.0; NA | NA; NA; NA; NA; NA; NA | G | (+); (+); (-); (+) | 7 |
| Robinson 2019 **S137** | UK | E | CAR | 1; 1; 0 | NA; NA; NA | 1; 0; 1; 0; 0; 0 | NA; NA; NA | NA; NA; NA; NA; NA; NA | G | (-); (-); (-); (-) | 4 |
| Sadat 2022 **S138** | USA | E | CAR | 1; 1; 0 | 3.0; 3.0; NA | 0; 1; 0; 1; 0; 0 | 65.0; 65.0; NA | 1; 0; 1; 0; 0; 0 | G | (-); (-); (-); (-) | 3 |
| Queirós 2022 **S139** | Portugal | E | CAR | 1; 1; 0 | 3.0; 3.0; NA | 1; 0; 1; 0; 0; 0 | NA; NA; NA | 1; 0; 1; 0; 0; 0 | C | (-); (-); (-); (-) | 1 |
| Mizukami 1992 **S140** | Japan | E | CAR | 1; 1; 0 | 20.0; 20.0; NA | 0; 1; 0; 1; 0; 0 | NA; NA; NA | 1; 0; 1; 0; 0; 0 | P | (+); (-); (-); (+) | 4 |
| Kobayashi 2012 **S141** | Japan | E | CAR | 1; 1; 0 | 6.0; 6.0; NA | 1; 0; 1; 0; 0; 0 | 68.0; 68.0; NA | 1; 0; 1; 0; 0; 0 | G | (+); (-); (-); (+) | 8 |
| Kim 2023 **S142** | South Korea | E | CAR | 1; 1; 0 | NA; NA; NA | NA; NA; NA; NA; 0; 0 | 62.0; 62.0; NA | NA; NA; NA; NA; NA; NA | G | (+); (-); (-); (+) | 6 |
| Singh 2024 **S143** | India | E | CAR | 1; 1; 0 | 17.0; 17.0; NA | 0; 1; 0; 1; 0; 0 | 60.0; 60.0; NA | NA; NA; NA; NA; NA; NA | G | (+); (+); (-); (-) | 3 |
| Kharat 2023 **S144** | India | E | CAR | 1; 1; 0 | 22.0; 22.0; NA | 1; 0; 1; 0; 0; 0 | 63.0; 63.0; NA | 1; 0; 1; 0; 0; 0 | G | (+); (+); (-); (-) | 2 |
| Sakata 1993 **S145** | Japan | J | CAR | 1; 1; 0 | 36.0; 36.0; NA | 0; 1; 0; 1; 0; 0 | NA; NA; NA | NA; NA; NA; NA; NA; NA | P | (+); (+); (-); (+) | 7 |
| Hirasawa 2002 **S146** | Japan | J | CAR | 1; 1; 0 | 35.0; 35.0; NA | 0; 1; 0; 1; 0; 0 | NA; NA; NA | 1; 0; 1; 0; 0; 0 | P | (+); (-); (-); (-) | 4 |
| Matsuo 2012 **S147** | Japan | J | CAR | 1; 1; 0 | 31.0; 31.0; NA | 0; 1; 0; 1; 0; 0 | NA; NA; NA | NA; NA; NA; NA; NA; NA | C | (-); (-); (-); (-) | 2 |
| Katsube 1987 **S148** | Japan | J | CAR | 1; 1; 0 | 27.0; 27.0; NA | 1; 0; 1; 0; 0; 0 | NA; NA; NA | 0; 1; 0; 1; 0; 0 | C | (+); (+); (-); (+) | 7 |
| Yoshida 1988 **S149** | Japan | J | CAR | 1; 1; 0 | 38.0; 38.0; NA | 0; 1; 0; 1; 0; 0 | NA; NA; NA | NA; NA; NA; NA; NA; NA | C | (+); (+); (-); (+) | 7 |
| Naito 1991 **S150** | Japan | J | CAR | 1; 1; 0 | 23.0; 23.0; NA | 0; 1; 0; 1; 0; 0 | NA; NA; NA | 0; 1; 0; 1; 0; 0 | P | (+); (-); (-); (-) | 2 |
| Washimi 1992 **S151** | Japan | J | CAC | 1; 1; 0 | 27.0; 27.0; NA | 0; 1; 0; 1; 0; 0 | NA; NA; NA | NA; NA; NA; NA; NA; NA | C | (-); (-); (-); (-) | 3 |
| Arai 1992 **S152** | Japan | J | CAR | 1; 1; 0 | 30.0; 30.0; NA | 1; 0; 1; 0; 0; 0 | NA; NA; NA | 1; 0; 1; 0; 0; 0 | C | (+); (-); (-); (-) | 4 |
| Takagi 1992 **S153** | Japan | J | CAR | 1; 1; 0 | 44.0; 44.0; NA | 1; 0; 1; 0; 0; 0 | NA; NA; NA | NA; NA; NA; NA; NA; NA | P | (+); (+); (-); (-) | 7 |
| Enokida 1993 **S154** | Japan | J | CAR | 1; 1; 0 | 19.0; 19.0; NA | 0; 1; 0; 1; 0; 0 | NA; NA; NA | 0; 1; 0; 1; 0; 0 | C | (+); (+); (-); (-) | 3 |
| Urushitani 1993 **S155** | Japan | J | CAR | 1; 1; 0 | 14.0; 14.0; NA | 0; 1; 0; 1; 0; 0 | NA; NA; NA | NA; NA; NA; NA; NA; NA | C | (+); (+); (-); (+) | 7 |
| Higashi 1994 **S156** | Japan | J | CAR | 1; 1; 0 | 6.8; 6.8; NA | 1; 0; 1; 0; 0; 0 | NA; NA; NA | NA; NA; NA; NA; NA; NA | G | (+); (+); (-); (-) | 2 |
| Yoshimoto 1995 **S157** | Japan | J | CAR | 1; 1; 0 | 43.0; 43.0; NA | 0; 1; 0; 1; 0; 0 | 64.0; 64.0; NA | NA; NA; NA; NA; NA; NA | P | (+); (-); (+); (-) | 4 |
| Sato 1996 **S158** | Japan | J | CAR | 1; 1; 0 | 17.0; 17.0; NA | 1; 0; 1; 0; 0; 0 | 57.0; 57.0; NA | NA; NA; NA; NA; NA; NA | G | (+); (-); (-); (-) | 4 |
| Asai 1998 **S159** | Japan | J | CAR | 1; 1; 0 | 73.0; 73.0; NA | 1; 0; 1; 0; 0; 0 | 66.0; 66.0; NA | 0; 0; 0; 0; NA; NA | G | (-); (-); (-); (-) | 3 |
| Wada 1998 **S160** | Japan | J | CAR | 1; 1; 0 | 2.0; 2.0; NA | 0; 1; 0; 1; 0; 0 | 73.0; 73.0; NA | 1; 0; 1; 0; 0; 0 | G | (+); (+); (+); (+) | 8 |
| Aoyagi 1999 **S161** | Japan | J | CAR | 1; 1; 0 | 2.0; 2.0; NA | 0; 1; 0; 1; 0; 0 | 78.0; 78.0; NA | 1; 0; 1; 0; 0; 0 | G | (+); (+); (-); (+) | 7 |
| Irino 1999 **S162** | Japan | J | CAR | 1; 1; 0 | 6.0; 6.0; NA | 0; 1; 0; 1; 0; 0 | 74.0; 74.0; NA | 1; 0; 1; 0; 0; 0 | G | (+); (+); (-); (+) | 6 |
| Shikama 2000 **S163** | Japan | J | CAR | 1; 1; 0 | 41.0; 41.0; NA | 0; 1; 0; 1; 0; 0 | NA; NA; NA | NA; NA; NA; NA; NA; NA | G | (+); (+); (-); (-) | 1 |
| Takayama 2002 **S164** | Japan | J | CAR | 1; 1; 0 | 3.0; 3.0; NA | 1; 0; 1; 0; 0; 0 | NA; NA; NA | NA; NA; NA; NA; NA; NA | C | (+); (-); (-); (+) | 3 |
| Yanagidate 2002 **S165** | Japan | J | CAR | 1; 1; 0 | 12.0; 12.0; NA | 1; 0; 1; 0; 0; 0 | NA; NA; NA | NA; NA; NA; NA; NA; NA | C | (+); (-); (-); (+) | 5 |
| Miura 2002 **S166** | Japan | J | CAR | 1; 1; 0 | 7.0; 7.0; NA | 1; 0; 1; 0; 0; 0 | NA; NA; NA | 1; 0; 1; 0; 0; 0 | G | (+); (+); (-); (+) | 4 |
| Kanayama 2004 **S167** | Japan | J | CAR | 1; 1; 0 | 0.6; 0.6; NA | 0; 1; 0; 1; 0; 0 | 74.0; 74.0; NA | NA; NA; NA; NA; NA; NA | G | (+); (-); (+); (+) | 7 |
| Hirose 2004 **S168** | Japan | J | CAR | 1; 1; 0 | 6.0; 6.0; NA | 0; 1; 0; 1; 0; 0 | NA; NA; NA | NA; NA; NA; NA; NA; NA | G | (+); (+); (+); (+) | 7 |
| Okada 2006 **S169** | Japan | J | CAR | 1; 1; 0 | 4.0; 4.0; NA | 1; 0; 1; 0; 0; 0 | NA; NA; NA | NA; NA; NA; NA; NA; NA | C | (-); (-); (-); (-) | 4 |
| Takeuchi 2006 **S170** | Japan | J | CAR | 1; 1; 0 | 3.0; 3.0; NA | 1; 0; 1; 0; 0; 0 | 75.0; 75.0; NA | 1; 0; 1; 0; 0; 0 | G | (+); (-); (-); (+) | 8 |
| Nakayama 2007 **S171** | Japan | J | CAR | 1; 1; 0 | 6.0; 6.0; NA | 0; 1; 0; 1; 0; 0 | 71.0; 71.0; NA | NA; NA; NA; NA; NA; NA | G | (+); (-); (-); (+) | 7 |
| Tominaga 2009 **S172** | Japan | J | CAR | 1; 1; 0 | 2.0; 2.0; NA | 0; 1; 0; 1; 0; 0 | 73.0; 73.0; NA | NA; NA; NA; NA; NA; NA | G | (+); (-); (-); (+) | 8 |
| Kurihara 2009 **S173** | Japan | J | CAR | 1; 1; 0 | 15.0; 15.0; NA | 0; 1; 0; 1; 0; 0 | NA; NA; NA | 0; 1; 0; 1; 0; 0 | C | (+); (-); (-); (+) | 7 |
| Matuura 2009 **S174** | Japan | J | CAR | 1; 1; 0 | 5.0; 5.0; NA | 0; 1; 0; 1; 0; 0 | NA; NA; NA | NA; NA; NA; NA; NA; NA | G | (-); (-); (-); (+) | 1 |
| Kizuki 2012 **S175** | Japan | J | CAR | 1; 1; 0 | 18.0; 18.0; NA | 1; 0; 1; 0; 0; 0 | NA; NA; NA | NA; NA; NA; NA; NA; NA | G | (+); (-); (-); (+) | 7 |
| Hamada 2014 **S176** | Japan | J | CAR | 1; 1; 0 | 7.0; 7.0; NA | 1; 0; 1; 0; 0; 0 | 69.0; 69.0; NA | 1; 0; 1; 0; 0; 0 | G | (+); (+); (-); (+) | 8 |
| Iba 2015 **S177** | Japan | J | CAR | 1; 1; 0 | 10.0; 10.0; NA | 0; 1; 0; 1; 0; 0 | 70.0; 70.0; NA | 0; 1; 0; 1; 0; 0 | G | (+); (+); (-); (+) | 7 |
| Uenishi 2019 **S178** | Japan | J | CAR | 1; 1; 0 | 19.0; 19.0; NA | 1; 0; 1; 0; 0; 0 | NA; NA; NA | 0; 1; 0; 1; 0; 0 | G | (+); (+); (-); (+) | 8 |
| Fukuda 2021 **S179** | Japan | J | CAR | 1; 1; 0 | 15.0; 15.0; NA | 0; 1; 0; 1; 0; 0 | NA; NA; NA | NA; NA; NA; NA; NA; NA | C | (+); (-); (-); (+) | 2 |
| Takai 2021 **S180** | Japan | J | CAR | 1; 1; 0 | NA; NA; NA | 1; 0; 1; 0; 0; 0 | NA; NA; NA | NA; NA; NA; NA; NA; NA | C | (-); (-); (-); (+) | 3 |
| Kimura 1974 **S181** | Japan | J | CAR | 1; 1; 0 | 9.0; 9.0; NA | 0; 1; 0; 1; 0; 0 | NA; NA; NA | 1; 0; 1; 0; 0; 0 | P | (+); (+); (-); (-) | 5 |

Reference of included studies is shown in **Supplementary Document 3** separately.

*: Language of the article. E: English. J: Japanese.

†: Study design. CAC: Case-control. CAR: Case report. CAS: Case series. COH: Cohort. CRO: Cross-sectional.

‡: Each cell shows (number of DRPLA patients); (number of DRPLA patients with epilepsy); (number of DRPLA patients without epilepsy).

§: Each cell shows (mean age at DRPLA onset in all patients); (mean age at DRPLA onset in patients with epilepsy); (mean age at DRPLA onset in patients without epilepsy).

‖: Each cell shows (number of male DRPLA patients); (number of female DRPLA patients); (number of male DRPLA patients with epilepsy); (number of female DRPLA patients with epilepsy); (number of male DRPLA patients without epilepsy); (number of female DRPLA patients without epilepsy).

¶: Each cell shows (mean number of CAG repeats in DRPLA patients); (mean number of CAG repeats in DRPLA patients with epilepsy); (mean number of CAG repeats in DRPLA patients without epilepsy).

#: Each cell shows (number of DRPLA patients with paternal inheritance); (number of DRPLA patients with maternal inheritance); (number of DRPLA patients with epilepsy and paternal inheritance); (number of DRPLA patients with epilepsy and maternal inheritance); (number of DRPLA patients without epilepsy with paternal inheritance); (number of DRPLA patients without epilepsy with maternal inheritance).

**: Diagnostic method. C: Clinical. G: Genetic. P: Pathological.

††: Each cell shows (the presence of a report of seizure characteristics); (the presence of a report of EEG findings); (the presence of a report of SEP findings); and (the presence of a report of antiseizure medication use).

‡‡: Risk of bias score.
