# Supplementary material for: Epilepsy in dentatorubral–pallidoluysian atrophy: A systematic review and meta‐analysis
Source: Epilepsia. 2025 Oct 28;67(2):696–711. doi: 10.1111/epi.18700 (PMC12927676; doi:10.1111/epi.18700)
Supplement: Supplementary file 1 — Data S1. [file EPI-67-696-s001.zip › epi18700-sup-0003-Supinfo1@DRPLA_Supplementary3_nk6_hi3_R_nk2.docx]

**Supplementary Document 3**

**in**

**Epilepsy in Dentatorubral-Pallidoluysian Atrophy: A Systematic Review and Meta-Analysis**

**It includes**

**Full list of references for all studies included in this systematic review**

1. Hasegawa A, Ikeuchi T, Koike R, Matsubara N, Tsuchiya M, Nozaki H, et al. Long-term disability and　prognosis in dentatorubral-pallidoluysian atrophy: A correlation with CAG repeat length. Mov Disord. 2010;25:1694–700. doi: 10.1002/mds.23167.
2. Ikeuchi T, Koide R, Tanaka H, Onodera O, Igarashi S, Takahashi H, et al. Dentatorubral-pallidoluysian atrophy: clinical features are closely related to unstable expansions of trinucleotide (CAG) repeat. Ann Neurol 1995;37:769–75. doi: 10.1002/ana.410370610.
3. Hirayama K, Takayanagi T, Nakamura R, Yanagisawa N, Hattori T, Kita K, et al. Spinocerebellar degenerations in Japan: a nationwide epidemiological and clinical study. Acta Neurol Scand Suppl. 1994;153:1–22. doi: 10.1111/j.1600-0404.1994.tb05401.x.
4. Amprosi M, Nachbauer W, Indelicato E, Eigentler A, Puttinger G, Gusenleitner J, et al. Dentatorubral-pallidoluysian atrophy outside of Asia: A case report of the first Austrian family harbouring this rare mutation. Mov Disord 2019;34(Suppl 1):S98.
5. Naito H, Oyanagi S. Familial myoclonus epilepsy and choreoathetosis: hereditary dentatorubral-pallidoluysian atrophy. Neurology. 1982;32:798–807. doi: 10.1212/wnl.32.8.798.
6. Komure O, Sano A, Nishino N, Yamauchi N, Ueno S, Kondoh K, et al. DNA analysis in hereditary dentatorubral-pallidoluysian atrophy: correlation between CAG repeat length and phenotypic variation and the molecular basis of anticipation. Neurology. 1995;45:143–9. doi: 10.1212/wnl.45.1.143.
7. Vale J, Bugalho P, Silveira I, Sequeiros J, Guimarães J, Coutinho P. Autosomal dominant cerebellar ataxia: frequency analysis and clinical characterization of 45 families from Portugal. Eur J Neurol. 2010;17:124–8. doi: 10.1111/j.1468-1331.2009.02757.x.
8. Onuma T, Katou M, Sekimoto M, Kato T, Adachi N, Kasai K. [Development of Diagnostic and Treatment Methods for Epilepsy Using New Technology: Clinical Study of Hereditary Epilepsy, Electrophysiological Examination of BAFME, Especially in Comparison with DRPLA] Shingijutsu wo mochiita tenkan tou no shindan hou to chiryouhou no kaihatsu idensei tenkan no rinshou BAFME no denki seirigakuteki kentou, toku ni DRPLA to no hikaku (in Japanese). Kouseishou seishin shinkeishikkan kenkyuu itakuhi ni yoru kenkyuu houkokusho (Annual report of the research on nervous and mental disorders) 2000;2.
9. Sano A, Yamauchi N, Kakimoto Y, Komure O, Kawai J, Hazama F, et al. Anticipation in hereditary dentatorubral-pallidoluysian atrophy. Hum Genet. 1994;93:699–702. doi: 10.1007/BF00201575.
10. Koide R, Ikeuchi T, Onodera O, Tanaka H, Igarashi S, Endo K, et al. Unstable expansion of CAG repeat in hereditary dentatorubral-pallidoluysian atrophy (DRPLA). Nat Genet. 1994;6:9–13. doi: 10.1038/ng0194-9.
11. Baba K. [EEG of Dentatorubral-Pallidoluysian Atrophy (DRPLA)] Shijoukaku sekikaku tansoukyuu Rui tai ishukushou (Dentatorubropallidoluysian atrophy:DRPLA) no nouha (in Japanese). Clinical Psychiatry 1987;29(5):523–31.
12. Yamaguchi N, Chikada A, Ishiura H, Mitsui J, Tsuji S, Toda T. Development of a clinical rating scale for DRPLA: insight from a retrospective study. Clin Neurol. 2020;60:S483. doi: 10.5692/clinicalneurol.60-supplement-S371.
13. Warner TT, Williams LD, Walker RW, Flinter F, Robb SA, Bundey SE, et al. A clinical and molecular genetic study of dentatorubropallidoluysian atrophy in four European families. Ann Neurol. 1995;37:452–9. doi: 10.1002/ana.410370407.
14. Contesse MG, Woods RJ, Leffler M, Prades S, Greenfield J, Compton A, et al. Understanding dentatorubral-pallidoluysian atrophy (DRPLA) symptoms and impacts on daily life: a qualitative interview study with patients and caregivers. Ther Adv Rare Dis. 2024;5:1–14. doi : 10.1177/26330040241252447.
15. Egawa K, Takahashi Y, Kubota Y, Kubota H, Inoue Y, Fujiwara T, et al. Electroclinical features of epilepsy in patients with juvenile type dentatorubral-pallidoluysian atrophy. Epilepsia. 2008;49:2041–9. doi: 10.1111/j.1528-1167.2008.01701.x.
16. Iwabuchi K, Yagishita S, Amano N. [Clinical Pathological Study of Autosomal Dominant Hereditary Dentatorubral-Pallidoluysian Atrophy (DRPLA): Proposal of Naito-Koyanagi Disease] Jousenshokutai yuusei idensei shijoukaku sekikaku tansoukyuu Rui tai ishukushou (DRPLA) no rinshou byourigakuteki kenkyuu Naitou Koyanagi byou no teishou (in Japanese). Shinkeikenkyuu no shimpo (Advances in Neurological Sciences). 1993;37(4):678–92.
17. Nielsen JE, Sørensen SA, Hasholt L, Nørremølle A. Dentatorubral-pallidoluysian atrophy. Clinical features of a five-generation Danish family. Mov Disord. 1996;11:533–41. doi: 10.1002/mds.870110508.
18. Wardle M, Majounie E, Williams NM, Rosser AE, Morris HR, Robertson NP. Dentatorubral pallidoluysian atrophy in South Wales. J Neurol Neurosurg Psychiatry. 2008;79:804–7. doi: 10.1136/jnnp.2007.128074.
19. Hayashi M, Kumada S, Shioda K, Fukatsu R. [Neuropathological Analysis of the Brainstem and Cerebral Cortex Lesions on Epileptogenesis in Hereditary Dentatorubral-Pallidoluysian Atrophy] Idensei shijoukaku sekikaku tansoukyuu Rui tai ishukushou ni okeru tenkan hassei ni kansuru noukan oyobi dainouhishitsu no shinkeibyourigakuteki kaiseki (in Japanese). Brain & Development. 2007;29(8):473–81. doi: 10.1016/j.braindev.2006.12.008.
20. Iwabuchi K. [Clinical Pathological Study of Dentatorubral-Pallidoluysian Atrophy] Shijoukaku sekikaku tansoukyuu Rui tai ishukushou no rinshou byourigakuteki kenkyuu (in Japanese). Yokohama Igaku (Yokohama Medical Journal). 1987;38(3):291–01.
21. Kasai K, Onuma T, Kato M, Kato T, Takeya J, Sekimoto M, et al. Differences in evoked potential characteristics between DRPLA patients and patients with progressive myoclonic epilepsy: preliminary findings indicating usefulness for differential diagnosis. Epilepsy Res. 1999;37:3–11.doi:10.1016/s0920-1211(99)00028-5.
22. Tomoda A, Ikezawa M, Ohtani Y. [Progressive Myoclonic Epilepsy: Dentato-Rubro-Pallido-Luysian Atrophy (DRPLA) in Childhood] Shinkousei kandaisei tenkan: shouniki ni okeru Dentato-Rubro-Pallido-Luysian Atrophy (DRPLA) (in Japanese). Brain & Development 1991;13(4):266–9.
23. Nørremølle A, Nielsen JE, Sørensen SA, Hasholt L. Elongated CAG repeats of the B37 gene in a Danish family with dentato-rubro-pallido-luysian atrophy. Hum Genet. 1995;95:313–8. doi:10.1007/BF00225200.
24. Takeda S, Takahashi H. Neuropathology of dentatorubropallidoluysian atrophy. Neuropathology. 1996;16:48-55. doi: 10.1111/j.1440-1789.1996.tb00155.x.
25. Muñoz E, Milà M, Sánchez A, Latorre P, Ariza A, Codina M, et al. Dentatorubropallidoluysian atrophy in a Spanish family: a clinical, radiological, pathological, and genetic study. J Neurol Neurosurg Psychiatry. 1999;67:811–4. doi: 10.1136/jnnp.67.6.811.
26. Takiguchi Y, Katayama S, Miyamoto M. [A Family of 11 Cases Suspected of Dentatorubropallidoluysian Atrophy (DRPLA)] Dentatorubropallidoluysian atrophy (DRPLA) to omowareru 1 kakei 11 shourei (in Japanese). Dokkyo Igakukai Zasshi (Dokkyou Medical Journal) 1992;7(2):283–9.
27. Naito K, Izawa K, Kurosaki T, Kaji S, Sawa M. [Two Families of Dominantly Inherited Progressive Myoclonic Epilepsy] Yuusei idengata shinkousei miokurounusu tenkan no 2 kakei (in Japanese). Seishin shinke igaku zasshi (Psychiatria et Neurologia Japonica). 1972;74(12):871–97.
28. Touge T, Ichihara N, Ishibashi T, Ikeguchi M, Sasaki I, Takeuchi H. Afferent and efferent excitabilities of the transcortical loop in patients with dentatorubral-pallidoluysian atrophy. J Neurol Sci. 2000;176:28–36. doi: 10.1016/s0022-510x(00)00294-x.
29. Miyata R, Hayashi M, Tanuma N, Shioda K, Fukatsu R, Mizutani S. Oxidative stress in neurodegeneration in dentatorubral-pallidoluysian atrophy. J Neurol Sci. 2008;264:133–9. doi:10.1016/j.jns.2007.08.025.
30. Becher MW, Rubinsztein DC, Leggo J, Wagster M V, Stine OC, Ranen NG, et al. Dentatorubral and pallidoluysian atrophy (DRPLA). Clinical and neuropathological findings in genetically confirmed North American and European pedigrees. Mov Disord. 1997;12:519–30. doi: 10.1002/mds.870120408.
31. Tokiguchi S, Naito K, Nagai H. [CT Findings of Hereditary Dentatorubral-Pallidoluysian Atrophy (DRPLA)] Idensei shijoukaku sekikaku tansoukyuu Rui tai ishukushou (DRPLA) no CT shoken (in Japanese). CT kenkyuu (Progress in Computerized Tomography). 1987;9(6):665–72.
32. Kasai K, Onuma T, Katou M, Kato T, Taketani J, Sekimoto M, et al. [Findings of Somatosensory and Auditory Brainstem Evoked Potentials in DRPLA Patients: Comparison with Other Progressive Myoclonic Epilepsies] Shijoukaku sekikaku tansoukyuu Rui tai ishukushou (DRPLA) kanja ni okeru taiseikankaku choukaku noukan yuuhatsu deni no shoken hoka no shinkousei miokurounusu tenkan to no hikaku (in Japanese). Tenkan kenkyuu (Journal of the Japan Epilepsy Society). 1998;16(3):184–92.
33. Maruyama S, Saito Y, Nakagawa E, Saito T, Komaki H, Sugai K, et al. Importance of CAG repeat length in childhood-onset dentatorubral-pallidoluysian atrophy. J Neurol. 2012;259:2329–34. doi: 10.1007/s00415-012-6493-7.
34. Mizoi R, Segawa F, Kamada K. [Examination of Cerebral White Matter Lesions in Dentatorubral-Pallidoluysian Atrophy: MRI Perfusion Study] Shijoukaku sekikaku tansoukyuu Rui tai ishukushou no dainou hakushitsu byouhen no kentou toku ni MRI perfusion study ni yoru kensaku (in Japanese). Nou to shinkei (Brain and Nerve). 1994;46(2):145–51.
35. Pinto WBV de R, Salomão RPA, Bergamasco NC, da Cunha Ribas G, da Graça FF, Lopes-Cendes I, et al. DRPLA: An unusual disease or an underestimated cause of ataxia in Brazil? Parkinsonism Relat Disord. 2021;92:67–71. doi: 10.1016/j.parkreldis.2021.10.004.
36. Tsuchiya K, Oyanagi S, Ikeda K. [Clinical Pathological Study of Eight Autopsy Cases of Dentatorubral-Pallidoluysian Atrophy: Clinical Pathological Correlation between Pyramidal Signs and Pyramidal Lesions] Shijoukaku sekikaku tansoukyuu Rui tai ishuku 8 boukenrei no rinshou byourigakuteki kenkyuu toku ni suitairo choukou to suitairo shougai to no aida no rinshou byourigakuteki soukan ni tsuite (in Japanese). NEUROPATHOLOGY. 1995;15(3):145–53.
37. Oda N, Kumode M, Kiuchi M, Nagae A, Taneshima A, Fujita Y, et al. [Long-term Clinical Course of Eight Cases of Childhood-Onset Juvenile Dentatorubral-Pallidoluysian Atrophy to Adulthood] Shouniki hasshou no jakunengata shijoukaku sekikaku tansoukyuu Rui tai ishukushou 8 shourei no seijinki made no chouki rinshou keika ni tsuite (in Japanese). Nihon juushou shinshin shougai gakkaishi (Journal of Severe Motor and Intellectual Disabilities). 2015;40(3):381–6.
38. Tsuchiya K, Oyanagi S, Arima K, Ikeda K, Akashi T, Ando S, et al. Dentatorubropallidoluysian atrophy: clinicopathological study of dementia and involvement of the nucleus basalis of Meynert in seven autopsy cases. Acta Neuropathol. 1998;96:502–8. doi: 10.1007/s004010050925.
39. Hayashi M, Nakajima K, Miyata R, Tanuma N, Kodama T. Lesions of acetylcholine neurons in refractory epilepsy. ISRN Neurol. 2012;2012:404263. doi: 10.5402/2012/404263.
40. Licht DJ, Lynch DR. Juvenile dentatorubral-pallidoluysian atrophy: new clinical features. Pediatr Neurol. 2002;26:51–4. doi: 10.1016/s0887-8994(01)00346-0.
41. Zhang J, Yang Y, Niu X, Chen J, Sun W, Ding C, et al. Clinical phenotype features and genetic etiologies of 38 children with progressive myoclonic epilepsy. Acta Epileptol. 2020;2. doi:10.1186/s42494-020-00023-z.
42. Aoki M, Abe K, Kameya T, Watanabe M, Itoyama Y. Maternal anticipation of DRPLA. Hum Mol Genet. 1994;3:1197–8. doi: 10.1093/hmg/3.7.1197.
43. Uyama E, Kondo I, Uchino M, Fukushima T, Murayama N, Kuwano A, et al. Dentatorubral-pallidoluysian atrophy (DRPLA): clinical, genetic, and neuroradiologic studies in a family. J Neurol Sci. 1995;130:146–53. doi: 10.1016/0022-510x(95)00019-x.
44. Kishimoto T, Higashiura N, Hiraoka Y. [Dentatorubral-Pallidoluysian Atrophy (DRPLA) And Midlatency Auditory Evoked Responses]. Nara igaku zasshi (Journal of Nara Medical Association). 1995;46(3):251–8.
45. Asai S, Kinoshita T, Murata A. [Study on EEG Spatial Structure in Dentatorubral-Pallidoluysian Atrophy: Using FFT-Dipole-Approximation Method] Shijoukaku sekikaku tansoukyuu Rui tai ishukushou ni okeru nouha kuukan kouzou no kentou FFT-Dipole-Approximation hou wo mochiite (in Japanese). Therapeutic Research. 1997;18(6):2085–90.
46. Saitoh S, Momoi MY, Yamagata T, Miyao M, Suwa K. Clinical and electroencephalographic findings in juvenile type DRPLA. Pediatr Neurol. 1998;18:265–8. doi: 10.1016/s0887-8994(97)00175-6.
47. Yam WKL, Wu NSP, Lo IFM, Ko CH, Yeung WL, Lam STS. Dentatorubral-pallidoluysian atrophy in two Chinese families in Hong Kong. Xianggang Yi Xue Za Zhi (Hong Kong Med J). 2004;10:53 6.
48. Nakano H, Kinoshita M, Sawada H. Clinical features of epilepsy in dentatorubral-pallidoluysian atrophy patients. Epilepsia. 2012;53:44-5. doi: 10.1111/j.1528-1167.2012.03677.x.
49. Kim H, Yun JY, Choi K-G, Koo H, Han HJ. Sleep Related Problems as a Nonmotor Symptom of Dentatorubropallidoluysian Atrophy. J Korean Med Sci. 2018;33:e130. doi: 10.3346/jkms.2018.33.e130.
50. Takahata N, Ito K, Yoshimura Y, Nishihori K, Suzuki H. Familial chorea and myoclonus epilepsy. Neurology. 1978;28:913–9. doi: 10.1212/wnl.28.9.913.
51. Honzawa S, Sugai K, Akaike H, Nakayama A, Fujikawa Y, Komaki H, et al. [Examination of 19 Cases of Neurodegenerative Metabolic Diseases with Learning Difficulties and Behavioral Abnormalities in School Age] Gakudouki ni gakushuu konnan ya koudou ijou de shohatsu shita shinkei hensei taisha shikkan 19 rei no kentou (in Japanese). Nou to hattatsu (Official Journal of the Japanese Society of Child Neurology). 2012;44(4):295–9.
52. Toyoshima I, Obara K, Abe E, Hatakeyama T, Takeda Y, Kobayashi M. [Experience with Perampanel: Focusing on Blood Concentration] Peranpaneru no shiyou keiken kecchuu noudo wo chuushin toshite (in Japanese). Akita byouin igaku zasshi (Journal of Akita National Hospital). 2020;8(3):23–8.
53. Takahashi Y, Fujiwara T, Yagi K, Seino M. Wavelength dependence of photoparoxysmal responses in photosensitive patients with epilepsy. Epilepsia. 1999;40 Suppl 4:23-7. doi: 10.1111/j.1528-1157.1999.tb00902.x.
54. Sone D, Sato N, Yokoyama K, Sumida K, Kanai M, Imabayashi E, et al. Striatal glucose hypometabolism in preadolescent-onset dentatorubral-pallidoluysian atrophy. J Neurol Sci. 2016;360:121–4. doi: 10.1016/j.jns.2015.12.002.
55. Warner TT, Lennox GG, Janota I, Harding AE. Autosomal-dominant dentatorubropallidoluysian atrophy in the United Kingdom. Mov Disord. 1994;9:289–96. doi: 10.1002/mds.870090302.
56. Potter NT, Meyer MA, Zimmerman AW, Eisenstadt ML, Anderson IJ. Molecular and clinical findings in a family with dentatorubral-pallidoluysian atrophy. Ann Neurol. 1995;37:273–7. doi: 10.1002/ana.410370220.
57. Nakayama T, Funatsuka M, Oguni H. [EEG Findings in Juvenile-Onset Dentatorubral-Pallidoluysian Atrophy] Jakunen hasshou shijoukaku sekikaku tansoukyuu Rui tai ishukushou no nouha shoken (in Japanese). Rinshou nouha (Clinical Electroencephalography). 1997;39(9):624–8.
58. Iwabuchi K, Amano N, Yagishita S. [A Family Case of Dentatorubral-Pallidoluysian Atrophy: Issues of the Ataxo-Choreoathetosis Type] Shijoukaku sekikaku tansoukyuu Rui tai ishukushou no 1 kazokurei ataxo-choreoathetosis gata no mondaite ni tsuite (in Japanese). Rinshou shinkeigaku (Clinical Neurology). 1987;27(8):1002–12.
59. Tamagaki C, Murata A, Sakata T. [A Family Case of Dentatorubral-Pallidoluysian Atrophy with Maternal Inheritance Showing Anticipation] Bokei iden de hyougen sokushin genshou wo teishita shijoukaku sekikaku tansoukyuu Rui tai ishukushou no 1 kakei (in Japanese). Osaka tenkan kenkyuukai zasshi (The Osaka journal of epilepsy research). 1996;7(1):29–34.
60. Otsuka H, Harada M, Hieda M. [Study Using Magnetic Resonance Imaging in Dentatorubral-Pallidoluysian Atrophy: MRI, Proton MR Spectroscopy, and Genetic Analysis in Relation to Age of Onset and Clinical Severity] Shijoukaku sekikaku tansoukyuu Rui tai ishukushou no jiki kyoumei genshou wo mochiita kentou MRI, proton MR Spectroscopy, idenshi kaiseki wo mochiita hasshou nenrei oyobi rinshou juushoudo to no kanrensei ni tsuite (in Japanese). Nou to shinkei (Brain and Nerve). 1996;48(9):818–23. doi; 10.11477/mf.1406900998.
61. Saitou K, Kawakami S, Maeda M. [A Family Case of Hereditary Dentatorubral-Pallidoluysian Atrophy] Idensei shijoukaku sekikaku tansoukyuu Rui tai ishukushou no 1 kakei ni tsuite (in Japanese). Takamatsu shimin byouin zasshi (Takamatsu Municipal Hospital Journal). 1997;13:31–7.
62. Sato K, Nobukuni K, Miyata S, Takada Y, Ihara Y, Namba R. [Four Cases of Dentatorubral-Pallidoluysian Atrophy with Epileptic Seizures: Rapid Progression of Neurological Symptoms Following Seizure Onset] Shijoukaku sekikaku tansoukyuu Rui tai ishukushou to tenkan hossa hossa shutsugen ni taiou shite shinkeishoujou no kyuusoku na shinkou ga mirareta 4 shourei (in Japanese). Tenkan kenkyuu (Journal of the Japan Epilepsy Society). 1999;17(2):121–7. doi; 10.3805/jjes.17.121.
63. Imamura J, Sugawara K, Matsuo N, Ito R, Nishida H. [Clinical Examination of a Family with Dentatorubral-Pallidoluysian Atrophy (DRPLA)] Shijoukaku sekikaku tansoukyuu Rui tai ishukushou (DRPLA) ikkakei no rinshouteki kentou (in Japanese). Gifu ken sougou iryou sentā nenpou (Annual of Gifu Prefectural General Medical Center). 2007;1(28):29–34.
64. Koyanagi S, Naito K. [Clinical and Pathological Consideration of Four Autopsy Cases Including a Parent-Child Case of Dominantly Inherited Myoclonic Epilepsy] Yuusei idengata miokurounusu tenkan no oyako rei wo fukumu 4 boukenrei to sono rinshou byourigakuteki kousatsu (in Japanese). Seishin shinkeigaku zasshi (Psychiatria et Neurologia Japonica). 1977;79(3):113–29.
65. Segawa F, Kinoshita M, Ishida T. [A Study of Motor Control Mechanisms in Dentatorubral-Pallidoluysian Atrophy (DRPLA)] Shijoukaku sekikaku tansoukyuu Rui tai ishukushou (DRPLA) ni okeru undou seigyo kikou no kentou (in Japanese). Rinshou nouha (Clinical Electroencephalography). 1991;33(3):183–9.
66. Asai S, Murata A, Sakata T. [Study on EEG Spatial Structure in Sibling Cases of Dentatorubral-Pallidoluysian Atrophy: Using FFT-Dipole-Approximation Method] Shijoukaku sekikaku tansoukyuu Rui tai ishukushou douhou rei ni okeru nouha kuukan kouzou no kentou FFT-Dipole-Approximation hou wo mochiite (in Japanese). Osaka tenkan kenkyuukai zasshi (The Osaka journal of epilepsy research). 1997;8(1):5–9.
67. Takahashi H, Ohama E, Naito H, Takeda S, Nakashima S, Makifuchi T, et al. Hereditary dentatorubral-pallidoluysian atrophy: clinical and pathologic variants in a family. Neurology. 1988;38:1065–70. doi: 10.1212/wnl.38.7.1065.
68. Mizukami K, Kawanishi Y, Tachikawa H, Arai T, Hori T, Sasaki M, et al. Dentatorubropallidoluysian atrophy with fourteen and six per second positive spikes on electroencephalogram. NEUROL PSYCHIATRY BRAIN RES. 1995;3:95–9.
69. Le Ber I, Camuzat A, Castelnovo G, Azulay J-P, Genton P, Gastaut J-L, et al. Prevalence of dentatorubral-pallidoluysian atrophy in a large series of white patients with cerebellar ataxia. Arch Neurol. 2003;60:1097–9. doi:10.1001/archneur.60.8.1097.
70. Vinton A, Fahey MC, O’Brien TJ, Shaw J, Storey E, Gardner RJM, et al. Dentatorubral-pallidoluysian atrophy in three generations, with clinical courses from nearly asymptomatic elderly to severe juvenile, in an Australian family of Macedonian descent. Am J Med Genet A. 2005;136:201–4. doi: 10.1002/ajmg.a.30355.
71. Wu KHC, Beran R, Procopis P, Davis M, Colley A. Dentatorubral-pallidoluysian atrophy (DRPLA) in an Australian family of Chinese descent. Twin Res Hum Genet. 2010;13:670.
72. Miguel R, Pelejão MR, Vale J, Pinto D. Dentatorubral pallidoluysiana atrophy: Study of a Portuguese family. Eur J Neurol. 2014;21:265.
73. Kutz C, Bundukamara C. Case series of 3 individuals of African descent with dentatorubral-pallidoluysian atrophy. Mov Disord. 2019;34:S213.
74. Amprosi M, Zech M, Lichtner P, Eckstein G, Unterberger I, Eigentler A, et al. The rare and the common: An Austrian DRPLA family harboring the European haplotype. Parkinsonism Relat Disord. 2021;87:119–21. doi: 10.1016/j.parkreldis.2021.04.024.
75. Arai K. [White Matter Lesions in Dentatorubral-Pallidoluysian Atrophy: Radiological and Neuropathological Study] Shijoukaku sekikaku tansoukyuu Rui tai ishuku ni okeru hakushitsu shougai houshasengakuteki oyobi shinkeibyourigakuteki kenkyuu (in Japanese). NEUROPATHOLOGY. 1995;15(3):154–62.
76. Miyazaki M, Hashimoto T, Nakagawa R. [Characteristic Evoked Potentials of Childhood Dentatorubral-Pallidoluysian Atrophy] Shouniki shijoukaku sekikaku tansoukyuu Rui tai ishukushou no tokuchou teki yuuhatsu deni (in Japanese). Brain & Development. 1996;18(5):389–93. doi; 10.1016/0387-7604(96)00039-3.
77. Sakahashi Y, Watanabe M, Fujiwara T. [Two Different Pathological Conditions of Photoparoxysmal Response in Hereditary Dentatorubral-Pallidoluysian Atrophy] Idensei shijoukaku sekikaku tansoukyuu Rui tai ishukushou ni okeru hikari hossa sei hannou no 2 shu no kotonaru byouriteki jouken (in Japanese). Brain & Development. 1997;19(4):285–9.
78. Harisankar K, Singh J, Mehta S, Lal V. Dentatorubral-pallidoluysian atrophy: a rare cause of epilepsy, ataxia and chorea. Pract Neurol. 2025;25:63–5. doi; 10.1136/pn-2024-004213.
79. Tokuda T, Ohara S, Hayashi R. [Clinical Pathological Study of Three Cases of Hereditary Dentatorubral-Pallidoluysian Atrophy] Idensei shijoukaku sekikaku tansoukyuu Rui tai ishukushou no 1 kakei 3 shourei no rinshou byourigakuteki kentou (in Japanese). Shinshuu igaku zasshi (The Shinshu Medical Journal). 1990;38(6):603–14.
80. Kawakatsu S. [Clinical Pathology of DRPLA: Examination of Two Families and Three Autopsy Cases] DRPLA no rinshou byouri 2 kakei 3 boukenrei no kentou kara (in Japanese). Shinkeibyourigaku (Neuropathology). 1991;11(3):189–99.
81. Miyanaga K, Yonemura K, Takagi M. [A Family Case of Dentatorubral-Pallidoluysian Atrophy] Shijoukaku sekikaku tansoukyuu Rui tai ishukushou no 1 kakei (in Japanese). Clinical Psychiatry 1995;37(5):515–8.
82. Nakamura K, Iwahashi K, Suwaki H. [A Family Case of Dentatorubral-Pallidoluysian Atrophy with Increased CAG Repeats from the Mother] Haha yurai de CAG ripiito ga akiraka ni fueta shijoukaku sekikaku tansoukyuu Rui tai ishukushou 1 kakei ni tsuite (in Japanese). Neurological Medicine 1995;43(4):339–42.
83. Iyoda K, Narahara K, Namba R. [A Case of Childhood-Onset Dentatorubral-Pallidoluysian Atrophy with Epilepsy: A Unique Disease Presentation with Electrical Status, Myoclonus, and Muscle Weakness] Shouniki hatsubyou no shijoukaku sekikaku tansoukyuu Rui tai ishukushou no tenkan electrical status wo tomonai miokurounusu to datsuryoku wo shuchou to suru tokui na byou zou wo teishita shourei (in Japanese). Tenkan kenkyu (Journal of the Japan Epilepsy Society). 1997;15(2):122–8.
84. Fujii K, Takanashi J, Saitou Y. [Examination of a Family with Dentatorubral-Pallidoluysian Atrophy: Clinical Features, Triplet Repeats, and MRI Correlation] Shijoukaku sekikaku tansoukyuu Rui tai ishukushou 1 kakei ni okeru kentou rinshouzou, triplet repeat, narabi ni MRI no kanren (in Japanese). Nou to hattatsu (Official Journal of the Japanese Society of Child Neurology). 1997;29(4):298–02. doi: 10.11251/ojjscn1969.29.298.
85. Takahara W, Ichihara N, Sato M. [Three Cases of Dentatorubral-Pallidoluysian Atrophy in a Family] Shijoukaku sekikaku tansoukyuu Rui tai ishukushou no 1 kakei 3 shourei (in Japanese). Kagawa ken naika ikaishi (The Journal of Kagawa Physicians' Association). 1997;33:50–3.
86. Murata K, Matsumura R, Nakamuro T. [A Family Case of Dentatorubral-Pallidoluysian Atrophy (DRPLA) with Anticipation but Decreased CAG Repeats] Shijoukaku sekikaku tansoukyuu Rui tai ishukushou (DRPLA) no 1 kakei anticipation ga mitomerarenagara CAG ripiito suu wa genshou shita 1 shourei (in Japanese). Rinshou shinkeigaku (Clinical Neurology). 1997;37(2):127–30.
87. Ishino H, Hirata J, Kumashiro N, Ohtahara S. [Three Sibling Cases of Myoclonic Epilepsy with One Autopsy: Degenerative Type] Miokurounusu tenkan no 3 douhou 1 boukenrei henseigata (in Japanese). Rinshou shinkeigaku (Clinical Neurology). 1973;13:27–34.
88. Sun S, Zhao W, Liu X. The relationship between the number of CAG repeats and clinical manifestations: a survey of Chinese DRPLA family. Acta Neurol Belg. 2023;123:1505–10. doi; 10.1007/s13760-023-02288-w.
89. Miyashita K, Inuzuka T, Ishikawa A. [A Family of Hereditary DRPLA: Clinical Symptomatic Differences Across Generations and Degenerative Findings in the Cerebral White Matter] Idensei DRPLA no 1 kakei sedaikan de no rinshou shoujou no sai to hottansha ni mitomerareta dainou hakushitsu no hensei shoken ni tsuite (in Japanese). Nou to shinkei (Brain and Nerve). 1992;44(3):279–84.
90. Miyazaki M, Kato T, Hashimoto T, Harada M, Kondo I, Kuroda Y. MR of childhood-onset dentatorubral-pallidoluysian atrophy. AJNR Am J Neuroradiol 1995;16:1834–6.
91. Hattori H, Higuchi Y, Okuno T, Asato R, Fukumoto M, Kondo I. Early-childhood progressive myoclonus epilepsy presenting as partial seizures in dentatorubral-pallidoluysian atrophy. Epilepsia. 1997;38:271–4. doi: 10.1111/j.1528-1157.1997.tb01116.x.
92. Lee IH, Soong BW, Lu YC, Chang YC. Dentatorubropallidoluysian atrophy in Chinese. Arch Neurol. 2001;58:1905–8. doi: 10.1001/archneur.58.11.1905.
93. Casseron W, Azulay JP, Broglin D, Kaphan E, Genton P, Le Ber I, et al. Phenotype variability in a caucasian family with dentatorubral-pallidoluysian atrophy. Eur Neurol. 2004;52:175–6. doi: 10.1159/000081859.
94. Morita T, Kotani H, Ishihara M, Naruse K, Fujieda M, Wakiguchi H, et al. Renal complications in two patients with dentatorubral-pallidoluysian atrophy. Clin Nephrol. 2007;67:44–8. doi: 10.5414/cnp67044.
95. Sunami Y, Koide R, Arai N, Yamada M, Mizutani T, Oyanagi K. Radiologic and neuropathologic findings in patients in a family with dentatorubral-pallidoluysian atrophy. Am J Neuroradiol. 2011;32:109–14. doi: 10.3174/ajnr.A2252.
96. Isobe N, Sakai Y, Kira R, Sanefuji M, Ishizaki Y, Sakata A, et al. Periodic Epileptiform Discharges in Children with Advanced Stages of Progressive Myoclonic Epilepsy. Clin EEG Neurosci. 2016;47:317–23. doi: 10.1177/1550059415579767
97. Zook-Lewis C, Greenwood R, Shiloh-Malawsky Y. Improved myoclonus and seizure control and dramatic improvement of neurological function in patients with dentatorubral pallidoluysian atrophy (DRPLA) treated with perampanel. Ann Neurol 2019;86:S140–1.
98. Alshimemeri S, Yoshida K, Visanji N, Rogaeva E, Munhoz R, Slow E, et al. Novel Dentato-Olivo-Luysian Atrophy in a Greek Family. Mov Disord 2020;35:S11. doi: 10.1002/mds.28268.
99. Iwabuchi K, Amano N, Yokoi S. [A Consideration of Two Families of Dentatorubral-Pallidoluysian Atrophy (Pseudo-Huntington's Chorea Type) (Hirayama)] Shijoukaku sekikaku tansoukyuu Rui tai ishukushou no 2 kakei pseudo-Huntington's chorea gata (Hirayama) ni tsuite no ichikousatsu (in Japanese). Rinshou shinkeigaku (Clinical Neurology). 1985;25(9):1052–60.
100. Akashi T, Andou J, Inose T. [Clinical Neuropathological Consideration of Dentatorubral-Pallidoluysian Atrophy (DRPLA)] Shijoukaku sekikaku tansoukyuu Rui tai ishukushou (DRPLA) no rinshou shinkeibyourigakuteki kousatsu (in Japanese). Rinshou seishin igaku (Japanese Journal of Clinical Psychiatry). 1987;16(8):1163–72.
101. Sakurai N, Takahashi A. [Two Types of Dentatorubral-Pallidoluysian Atrophy] Shijoukaku sekikaku tansoukyuu Rui tai ishukushou no 2 gata (in Japanese). Rinshou nouha (Clinical Electroencephalography). 1990;32(9):617–9.
102. Sasaki Y, Yamashita S, Iwamoto H. [A Case of Sisters with Cerebellar Ataxia, Myoclonus, and Intractable Epilepsy Showing Progressive Course] Shounou shicchoushou de hajimari miokurounusu to nanchisei tenkan wo mitome, shinkousei keika wo shimeshita shimairei (in Japanese). Kanagawa Children's Medical Center Journal 1995;24(4):254–63.
103. Yamashita S, Iwamoto H, Hara M. [A Case of Sisters with Hereditary Dentatorubral-Pallidoluysian Atrophy Onset in Infancy: Correspondence between DNA Analysis Findings and Clinical Pathological Findings] Youjiki hasshou no idensei shijoukaku sekikaku tansoukyuu Rui tai ishukushou no shimairei DNA kaiseki shoken to rinshou byouri shoken to no taiou (in Japanese). Nou to hattatsu (Official Journal of the Japanese Society of Child Neurology). 1995;27(6):473–9. doi; 10.11251/ojjscn1969.27.473.
104. Sasagawa M. [Two Cases of Juvenile Dentatorubral-Pallidoluysian Atrophy] Jakunen gata shijoukaku sekikaku tansoukyuu Rui tai ishukushou no 2 rei (in Japanese). Rinshou iden kenkyuu (Medical Genetics Research). 1997;19(1):31–8.
105. Nishio H, Wada H, Lee M, Matsuo T. [Analysis of CAG Repeats in the DRPLA Gene in a Family with Dentatorubral-Pallidoluysian Atrophy: Experience with Presymptomatic Diagnosis] Shijoukaku sekikaku tansoukyuu Rui tai ishukushou no 1 kakei ni okeru DRPLA idenshi no CAG ripiito no kaiseki hasshoumae shindan no keiken (in Japanese). Nou to hattatsu (Official Journal of the Japanese Society of Child Neurology). 1998;30(6):512–6. doi; 10.11251/ojjscn1969.30.512.
106. Sugie K, Nakamuro T, Harada N. [A Case of Siblings with Dentatorubral-Pallidoluysian Atrophy Complicated by Ehlers-Danlos Syndrome Type III] Shijoukaku sekikaku tansoukyuu Rui tai ishukushou ni Ehlers-Danlos shoukougun III gata wo gappeishita shiteirei (in Japanese). Rinshou shinkeigaku (Clinical Neurology). 1998;38(3):233–7.
107. Shimode Y, Ueki A, Gotou K, Nakajima T, Shinjo H, Miwa C. [Dentatorubral-Pallidoluysian Atrophy with Increased CAG Repeats through Maternal Transmission] maternal transmission de CAG ripiito ga zouka shita shijoukaku sekikaku tansoukyuu Rui tai ishukushou (in Japanese). Saishin seishin igaku (The Japanese Journal of Psychiatry). 2000;5(5):493–7.
108. Miyazaki K, Iijima M, Shibata K, Uchiyama S, Takemiya T, Iwata M. [A Case of Parent and Child with Different Clinical Types of Dentatorubral-Pallidoluysian Atrophy (DRPLA): Examination of Genetic Mutations and Clinical Types] Kotonaru rinshou gata wo teishita shijoukaku sekikaku tansoukyuu Rui tai ishukushou (DRPLA) no oyakorei idenshi hen'i to rinshou gata no kentou (in Japanese). Toukyou joshi ika daigaku zasshi (Journal of Tokyo Women's Medical University). 2000;70:E372–5.
109. Hashi R, Nakamura A, Sugimoto T, Kaneko I. [A Case of Sisters with Infantile-Onset Juvenile Dentatorubral-Pallidoluysian Atrophy (DRPLA): Examination of Temporal EEG Changes] Nyuujiki ni hasshou shita jakunensei shijoukaku sekikaku tansoukyuu Rui tai ishukushou (DRPLA) no shimairei keijiteki nouha henka no kentou (in Japanese). Nou to hattatsu (Official Journal of the Japanese Society of Child Neurology). 2007;39(6):445–9.
110. Naito K, Tanaka M, Hirose S, Koyanagi S. [Two Autopsy Cases of Degenerative Myoclonic Epilepsy with Chorea-Athetosis: Proposal of Hereditary Dentate-Pallidoluysian Atrophy] Butoubyou atetooze you undou wo tomonatta henseigata miokurounusu tenkan 2 boukenrei: idensei shijoukaku tansoukyuu kei shukushou no teishou (in Japanese). Seishin shinkeigaku zasshi (Psychiatria et Neurologia Japonica).1977;79(4):193–04.
111. Miyahara A, Saito Y, Sugai K, Nakagawa E, Sakuma H, Komaki H, et al. Reassessment of phenytoin for treatment of late stage progressive myoclonus epilepsy complicated with status epilepticus. Epilepsy Res. 2009;84:201–9. doi: 10.1016/j.eplepsyres.2009.02.010.
112. Oi K, Neshige S, Hitomi T, Kobayashi K, Tojima M, Matsuhashi M, et al. Low-dose perampanel improves refractory cortical myoclonus by the dispersed and suppressed paroxysmal depolarization shifts in the sensorimotor cortex. Clin Neurophysiol. 2021;132:e86–7. doi: 10.1016/j.clinph.2021.02.189.
113. Qu R, Dai Y, Qu X, Li Y, Shao X, Zhou R, et al. Use of perampanel in children with refractory epilepsy of genetic aetiology. Epileptic Disord. 2022;24:687–95. doi: 10.1684/epd.2022.1443.
114. Suzuki S, Kamoshita S, Ninomura S. Ramsay Hunt syndrome in dentatorubral-pallidoluysian atrophy. Pediatr Neurol. 1985;1:298–301. doi: 10.1016/0887-8994(85)90032-3.
115. Pfeiffer RF, McComb RD. Dentatorubro-pallidoluysian atrophy of the myoclonus epilepsy type with posterior column degeneration. Mov Disord. 1990;5:134–8. doi: 10.1002/mds.870050207.
116. Imamura A, Ito R, Tanaka S, Fukutomi O, Shimozawa N, Nishimura M, et al. High-intensity proton and T2-weighted MRI signals in the globus pallidus in juvenile-type of dentatorubral and pallidoluysian atrophy. Neuropediatrics. 1994;25:234–7. doi: 10.1055/s-2008-1073027.
117. Imamura A, Sugai K, Watanabe S, Hamada F, Kurashige T, Takashima S. High intensity in the globus pallidus on proton and T2-weighted MRI in a case of dentato-ruburo-pallido-luysian atrophy of myoclonus epilepsy type. Acta Paediatr Jpn Overseas Ed. 1994;36:527–30. doi: 10.1111/j.1442-200x.1994.tb03240.x.
118. Shimizu N, Yamami T, Nakayama M, Ikeuchi T, Koide R, Tsuji S. A sporadic case of dentatorubral pallidoluysian atrophy (DRPLA) with CAG repeat expansion but no clinical abnormalities in the father. J Neurol Neurosurg Psychiatry. 1996;61:113–4. doi: 10.1136/jnnp.61.1.113.
119. Villani F, Gellera C, Spreafico R, Castellotti B, Casazza M, Carrara F, et al. Clinical and molecular findings in the first identified Italian family with dentatorubral-pallidoluysian atrophy. Acta Neurol Scand. 1998;98:324–7. doi: 10.1111/j.1600-0404.1998.tb01742.x.
120. Cox H, Costin-Kelly NM, Ramani P, Whitehouse WP. An established case of dentatorubral pallidoluysian atrophy (DRPLA) with unusual features on muscle biopsy. Eur J Paediatr Neurol Off J Eur Paediatr Neurol Soc. 2000;4:119–23. doi: 10.1053/ejpn.2000.0279.
121. Takano T, Okuno K, Maruo Y, Takeuchi Y. A pediatric patient with sporadic dentatorubral pallidoluysian atrophy. Pediatr Neurol. 2003;28:72–3. doi: 10.1016/s0887-8994(02)00496-4.
122. Watarai M, Hashimoto T, Yamamoto K, Matsumoto Y, Tada T, Ikeda S. Pallidotomy for severe generalized chorea of juvenile-onset dentatorubral-pallidoluysian atrophy. Neurology. 2003;61:1452–4. doi: 10.1212/01.wnl.0000094202.26313.73.
123. Jung DS, Lee J-H, Lee J-E, Park H-J, You HW, Lee JS. Corneal endothelial changes as a clinical diagnostic indicator of dentatorubropallidoluysian atrophy. Cornea. 2004;23:210–4. doi: 10.1097/00003226-200403000-00018.
124. Brunetti-Pierri N, Wilfong AA, Hunter J V, Craigen WJ. A severe case of dentatorubro-pallidoluysian atrophy (DRPLA) with microcephaly, very early onset of seizures, and cerebral white matter involvement. Neuropediatrics. 2006;37:308–11. doi: 10.1055/s-2006-955967.
125. Kobayashi K, Hata H, Oka M, Ito M, Yoshinaga H, Kashihara K, et al. Age-related electrical status epilepticus during sleep and epileptic negative myoclonus in DRPLA. Neurology. 2006;66:772–3. doi: 10.1212/01.wnl.0000200958.30060.36.
126. Takamure M, Hirano M, Taoka T, Ueno S. White matter T2 hyperintensity development and clinical deterioration after status epilepticus in a patient with dentatorubral-pallidoluysian atrophy. Clin Neurol Neurosurg. 2006;108:482–5. doi: 10.1016/j.clineuro.2005.01.011.
127. Yiş U, Dirik E, Gündoğdu-Eken A, Başak AN. Dentatorubral pallidoluysian atrophy in a Turkish family. Turk J Pediatr. 2009;51:610–2.
128. Sudo K, Hata D, Yokoyama N, Kawashima J, Yabe I, Tajima Y, et al. Absence seizures with myoclonic seizures as an early manifestation of dentato-rubro-pallido-luysian atrophy (DRPLA): a follow-up clinical course of twelve years. Acta Neurol Belg. 2010;110:84–8.
129. Simpson M, Smith A, Kent H, Roxburgh R. Distinctive MRI abnormalities in a man with dentatorubral-pallidoluysian atrophy. J Neurol Neurosurg Psychiatry. 2012;83:529–30. doi: 10.1136/jnnp-2011-301612.
130. Saito T, Sugai K, Sasaki M, Shibuya M, Saito Y. Neuroaxonal dystrophy in dorsal column nucli in a patient with juvenile dentatorubro-pallidoluysian atrophy (DRPLA). Neuropathology. 2013;33:358. doi: 10.1111/neup.12034.
131. Zadori D, Tanczos T, Jakab K, Vecsei L, Klivenyi P. The first identified Hungarian patient with dentatorubralpallidoluysian atrophy (DRPLA). Mov Disord. 2014;29:S198. doi: 10.1002/mds.25914.
132. Nam TM, Cho KR, Youn J, Cho JW, Lee J Il. Deep brain stimulation in a dentatorubral-pallidoluyisian atrophy patient with myoclonic dystonia. J Clin Neurosci. 2015;22:1976–8. doi: 10.1016/j.jocn.2015.04.010.
133. Souza PVS De, Batistella GNDR, Pinto WBVDR, Oliveira ASB. Teaching Neuro Images: Leukodystrophy and progressive myoclonic epilepsy disclosing DRPLA. Neurology. 2016;86:e58–9. doi: 10.1212/WNL.0000000000002356.
134. Shiraishi H, Egawa K, Ito T, Kawano O, Asahina N, Kohsaka S. Efficacy of perampanel for controlling seizures and improving neurological dysfunction in a patient with dentatorubral-pallidoluysian atrophy (DRPLA). Epilepsy Behav Case Reports. 2017;8:44–6. doi: 10.1016/j.ebcr.2017.05.004.
135. Narita Z, Sumiyoshi T. Successful treatment of psychosis in dentatorubralpallidoluysian atrophy with quetiapine: A case report. Neuropsychopharmacol Reports. 2018;38:44–6. doi: 10.1002/npr2.12005
136. Nandanwar D, George E, Basha M. Nocturnal hyperkinetic spells in dentatorubral pallidoluysian atrophy. Neurology.c 2018;90. doi; 10.1212/WNL.90.15_supplement.P1.079.
137. Robinson R, Patton RC, Hemingway C. The 100000 genome project: More questions than answers? Dev Med Child Neurol. 2019;61:103. doi: 10.1111/dmcn.14120.
138. Sadat R, Hull M, Parnes M, Emrick L. eP226: Dual diagnoses in neurogenetics- A case series of pediatric movement disorders and clinical management. Gen Med. 2022;24:S140–1. doi: 10.1016/j.gim.2022.01.262.
139. Queirós H, Carneiro I, Martins E, Morgadinho A, Pereira D, Cordeiro G. MR imaging features of Dentatorubral-pallidoluysian atrophy (DRPLA): A case report. Mov Disord. 2022;37:S33. doi: 10.1002/mds.29223.
140. Mizukami K, Sasaki M, Shiraishi H. [An Autopsy Case of Dentatorubral-Pallidoluysian Atrophy (DRPLA) with Atypical Pathological Findings] Hiteikeiteki byourigakuteki shoken wo teishita shijoukaku sekikaku tansoukyuu Rui tai ishukushou no 1 boukenrei (in Japanese). The Japanese Journal of Psychiatry and Neurology. 1992;46(3):749–54.
141. Kobayashi K, Takeuchi A, Oka M, Akiyama M, Ohtsuka Y. [Amelioration of Disabling Myoclonus in a Case of DRPLA by Levetiracetam] DRPLA shourei ni okeru, kinou shougai wo hikiokosu miokurounusu no rebechirasetamu ni yoru kaizen (in Japanese). Brain & Development. 2012;34(5):368–71. doi; 10.1016/j.braindev.2011.07.013.
142. Kim BK, Park JM. Detatorubral pallidoluysian atrophy with cognitive impairment, epilepsy, movement disorders, and psychosis – a case. Neurocase. 2025;1–3. doi; 10.1080/13554794.2024.2447116.
143. Singh J, Mehta S, Lal V. A rare case of genetically confirmed dentatorubral-pallidoluysian atrophy from North India. Ann Mov Disord. 2024;7:S34–5. doi; 10.4103/aomd.aomd_14_24.
144. Kharat S, Ojha P, Patil S, Ansari A, Aglave V, Nagendra S. Case Report: Ataxia, Movement Disorder, and Epilepsy in a Young Male - An Interesting Combination Diagnosed as Genetically Confirmed. Ann Indian Acad Neurol. 2023;26:S153.
145. Sakata T, Murata A, Kashii Y, Tamagaki C, Watanabe S, Saito M. [A familial case of DRPLA diagnosed by an autopsy associated with hemoglobinopathy (Hb Takamatsu)]. Ijou kesshikiso shou (Hb Takamatsu) wo mitome bouken nite shindan ga kakutei sareta DRPLA no 1 kakei (in Japanese). Rinsho Shinkeigaku (Clinical Neurology). 1993;33:777–80.
146. Hirasawa M, Ikebe S, Komatsuzaki Y, Takanashi M, Mori H, Urabe T, et al. [A 52-year-old woman with dyskinesia, epilepsy and gait disturbance]. Fuzuii undou, tankan, chihou, hokoushougai wo teishita 52 sai josei (in Japanese). No To Shinkei (Brain and Nerve). 2002;54:919–27.
147. Matsuo K, Itoh K, Fujii C, Yamada T, Miyata K, Yamamoto Y, et al. An autopsy case of dentatorubralpallidoluysian atrophy presenting intraneuronal accumulation of polyglutamine in the widespread areas of central nervous system. Neuropathology. 2012;32:369. doi: 10.1111/j.1440-1789.2012.01324.x.
148. Katsube T, Kobayashi S, Yamaguchi S. [A Clinically Considered Case of Dentatorubropallidoluysian Atrophy (DRPLA)] Rinshouteki ni Dentatorubropallidoluysian Atrophy (DRPLA) to kangaerareta 1 rei (in Japanese). CT kenkyu (Progress in Computerized Tomography). 1987;9(3):330–5.
149. Yoshida N, Harada K, Nakanishi T. [A Case of Dentatorubral-Pallidoluysian Atrophy with Photogenic Epilepsy] Hikari kabinsei tenkan wo gappeishita shijoukaku sekikaku tansoukyuu Rui tai ishukushou no 1 rei (in Japanese). Rinshou nouha (Clinical Electroencephalography). 1988;30(9):624–6.
150. Naito H, Inazuki G. [Psychiatric Symptoms of DRPLA (Dentatorubropallidoluysian Atrophy)] DRPLA (dentatorubropallidoluysian atrophy) no seishin shoujou (in Japanese). Rinshou seishin igaku (Japanese Journal of Clinical Psychiatry). 1991;20(8):1195–04.
151. Washimi Y, Kaneoke Y, Sakurai N. [Motor-Related Potentials in Cerebellar Dentate Nucleus Degeneration] Shounou shijoukakukei henseishou no undoukanren noudeni (in Japanese). Rinshou shinkei (Clinical Neurology). 1992;32(6):567–71.
152. Arai T, Ichikawa T, Mizukami K. [A Case of DRPLA with 14Hz Positive Spikes] 14Hz yousei kyokuha wo mitometa DRPLA no 1 rei (in Japanese). Rinshou nouha. (Clinical Electroencephalography). 1992;34(8):552–6.
153. Takagi T, Nakamura J, Kitamoto T. [A Case of Multi-System Disease Suspected as Dentatorubral-Pallidoluysian Atrophy] Takeitousei shikkan shijoukaku sekikaku tansoukyuu Rui tai ishukushou to kangaerareru 1 rei (in Japanese). Kyuushuu shinkei seishin igaku (Kyushu Neuropsychiatry). 1992;38(3):320–7.
154. Enokida M, Takehara Y, Aikawa H. [A Familial Case Suspected as Dentatorubral-Pallidoluysian Atrophy (DRPLA)] Kazokusei ni hasshou shi shijoukaku sekikaku tansoukyuu Rui tai ishukushou (DRPLA) ga utagawareta 1 shourei (in Japanese). Seishinka chiryougaku (Japanese Journal of Psychiatric Treatment). 1993;8(11):1380–4.
155. Urushiya M, Kawamura K, Kageyama T. [A Case of Progressive Myoclonic Epilepsy (PME) Suspected as Sporadic Dentatorubral-Pallidoluysian Atrophy (DRPLA)] Kohatsusei shijoukaku sekikaku tansoukyuu Rui tai ishukushou (DRPLA) to kangaerareta shinkousei miokurounusu tenkan (PME) no 1 rei (in Japanese). Sumitomo byouin igakuzasshi (The Medical Journal of Sumitomo Hospital). 1993;1(20):89–95.
156. Higashi K. [A Case of Hereditary Dentatorubral-Pallidoluysian Atrophy (HDRPLA) Diagnosed by DNA Analysis] DNA bunseki nite idensei shijoukaku sekikaku tansoukyuu Rui tai ishukushou (HDRPLA) to shindan shieta 1 rei (in Japanese). Kodomo iryou sentā igakushi (Kanagawa Children's Medical Center Journal). 1994;23(3):192–5.
157. Zenmoto H, Sahara M, Tanaka K. [A Case of Sporadic Dentatorubral-Pallidoluysian Atrophy (DRPLA) Diagnosed through Gene Analysis] Idenshi shindan ga yuuyou de atta kohatsurei to kangaerareta shijoukaku sekikaku tansoukyuu Rui tai ishukushou (DRPLA) no 1 rei (in Japanese). Rinsho shinkeigaku (Clinical Neurology). 1995;35(2):201–3.
158. Sato K, Hayahara T, Namba R. [A Case of Dentatorubral-Pallidoluysian Atrophy with Homologous Triplet Repeats: Comparison with Clinical Symptoms and Triplet Repeat Number in Other Cases] Triplet repeat no homo setsugou wo teishita shijoukaku sekikaku tansoukyuu Rui tai ishukushou no 1 shourei ta shourei no rinshou shoujou, triplet repeat suu to hikakushite (in Japanese). Iryo (Japanese Journal of National Medical Services). 1996;50(1):40–3.
159. Asai S, Murata A, Kashiwai Y. [An Autopsy Case of Late-Onset Dentatorubral-Pallidoluysian Atrophy (DRPLA)] Kourei hasshou shita shijoukaku sekikaku tansoukyuu Rui tai ishukushou (DRPLA) no 1 boukenrei (in Japanese). Rounen seishin igaku zasshi (Japanese Journal of Geriatric Psychiatry) 1998;9(1):67–71.
160. Wada Y, Matsuoka T, Imai K, Taniike M, Mano T, Ono J, et al. [A Case of Juvenile Dentatorubral-Pallidoluysian Atrophy (DRPLA) with Psychomotor Developmental Delay from Infancy] Nyuuyoujiki kara seishin undou hattatsu chitai wo mitometa jakunengata shijoukaku sekikaku tansoukyuu Rui tai ishukushou (DRPLA) no 1 rei (in Japanese). Nou to hattatsu (Official Journal of the Japanese Society of Child Neurology). 1998;30(6):543–8.
161. Aoyagi K, Rinbe H, Ikeuchi K, Aihara M, Sada Y, Nakazawa S. [A Case of a Young Girl with Dentatorubral-Pallidoluysian Atrophy Diagnosed Early through Gene Analysis ‘Neurological Disease’] ‘Nou shinkeishikkan’ idenshi kaiseki ni yori souki ni shindan shieta shijoukaku sekikaku tansoukyuu Rui tai ishukushou no 1 joji rei (in Japanese). Shounika rinshou (Japanese Journal of Pediatrics) 1999;52(6):1043–7.
162. Irino T, Miyagishi T, Tsusaka K, Matsumoto A, Yabe I, Sudo K, et al. [A Case of Juvenile DRPLA Diagnosed through Gene Analysis] Idenshi kaiseki ga shindan ni yuuyou de atta jakunengata DRPLA no 1 rei (in Japanese). Shiritsu Sapporo byouin ishi (The Journal of Sapporo City General Hospital). 1999;59(2):167–72.
163. Shikama Y, Katagiri T, Moriya Y. [A Case of Dentatorubral-Pallidoluysian Atrophy (DRPLA): Changes in EEG Findings Over Time] Shijoukaku sekikaku tansoukyuu Rui tai ishukushou (DRPLA) no 1 rei nouha shoken no keijiteki henka ni tsuite (in Japanese). Yamagata kenritsu byouin igakuzasshi (Journal of Yamagata National Hospital). 2000;34(1):29–31.
164. Takayama N, Iwase Y, Sakio H. [Anesthesia Experience in Patients with Dentatorubral-Pallidoluysian Atrophy] Shijoukaku sekikaku tansoukyuu Rui tai ishukushou kanja no masui keiken (in Japanese). The Japanese Journal of Anesthesiology 2002;51(8):902–3.
165. Yanagidate F, Dohi S, Hamaya Y, Ueda N. [A Case of Dentatorubral-Pallidoluysian Atrophy with Increased Tonic Seizures and Involuntary Movements During Anesthesia Recovery] Masui kakuseiji ni kyouchokusei hossa to fuzii undou no zouchou wo mitometa shijoukaku sekikaku tansoukyuu Rui tai ishukushou no 1 shourei (in Japanese). The Japanese Journal of Anesthesiology 2002;51(5):532–4.
166. Miura T, Ishikawa Y, Ishikawa Y, Minami R, Kimura M, Yamaguchi S. [A Case of Dentatorubral-Pallidoluysian Atrophy in a Child with Idiosyncratic Liver Injury Due to Valproic Acid Observed During Carnitine Administration] Karunichin touyochuu ni mirareta barupuro san ni yoru tokuitaishitsu teki kan shougai no shijoukaku sekikaku tansoukyuu Rui tai ishukushou ji no 1 rei (in Japanese). Japanese Journal of Pediatrics 2002;65(4):683–6.
167. Kanayama M, Tsukamoto T, Miyaji T, Hamaguchi K, Fujimoto S, Ishikawa T. [A Case of Dentatorubral-Pallidoluysian Atrophy (DRPLA) with Developmental Delay from Late Infancy] Nyuujiki kouhan kara hattatsu chitai wo mitometa shijoukaku sekikaku tansoukyuu Rui tai ishukushou (DRPLA) no 1 rei (in Japanese). Nou to hattatsu (Official Journal of the Japanese Society of Child Neurology). 2004;36(5):407–12.
168. Hirose M, Yokoyama H, Noguchi R, Haginoya K, Aoki M, Iinuma K. [A Case of Dentatorubral-Pallidoluysian Atrophy with Effective Piracetam for Action Myoclonus] Piracetam ga dousasei miokurounusu ni yuukou de atta shijoukaku sekikaku tansoukyuu Rui tai ishukushou no 1 rei (in Japanese). Nou to hattatsu (Official Journal of the Japanese Society of Child Neurology). 2004;36(1):75–9.
169. Okada M, Nakagawa E, Masuyama T, Fujikawa Y, Komaki H, Sugai K. [Examination of Three Cases of Severe Mental and Physical Disabilities with Unilateral Pleural Effusion During Long-Term Dantrolene Administration] Dantrolene chouki touyochuu ni hensokusei kyousui choryuu wo kitashita juushou shinshin shougaisha 3 shourei no kentou (in Japanese). Nou to hatatsu (Official Journal of the Japanese Society of Child Neurology). 2006;38(1):39–43. doi: 10.11251/ojjscn1969.38.39.
170. Takeuchi T, Moroboshi T, Chiba S, Noguchi S, Takayama R, Kato T, et al. [A Case of Dentatorubral-Pallidoluysian Atrophy with Acute Renal Failure] Kyuusei jinfuzenn wo teishita shijoukaku sekikaku tansoukyuu Rui tai ishukushou no 1 rei (in Japanese). Rinshou shouni igaku (The Journal of Clinical Pediatrics, Sapporo). 2006;54(3):78–82.
171. Nakayama T, Funatsuka M, Hayashi K, Saito K, Oguni H, Osawa M. [TRH Therapy for Involuntary Movements in Juvenile-Onset Dentatorubral-Pallidoluysian Atrophy] Jakunen hasshou shijoukaku sekikaku tansoukyuu Rui tai ishukushou no fuzui undou ni taisuru TRH ryouhou (in Japanese). Tenkan kenkyuu (Journal of the Japan Epilepsy Society). 2007;25(1):4–9. doi; 10.3805/jjes.25.4.
172. Tominaga Y, Iwatani S, Kitai M, Hashimoto N, Shintani K, Shimono K, et al. [A Case of Dentatorubral-Pallidoluysian Atrophy Treated with Ketogenic Diet Therapy] Keton shoku ryouhou wo okonatta shijoukaku sekikaku tansoukyuu Rui tai ishukushou no 1 rei (in Japanese). Osaka tenkan kenkyuukai zasshi (The Osaka journal of epilepsy research). 2009;20(1):27–32.
173. Kurihara M, Takahashi K, Kohagisawa T, Yamauchi Y, Ida H. [Long-term Follow-up of a 28-Year-Old Female with Dentatorubral-Pallidoluysian Atrophy (DRPLA): Utility of Gait Analysis] Chouki keika kansatsu wo shita shijoukaku sekikaku tansoukyuu Rui tai ishukushou (DRPLA) no 28 sai josei hokou bunseki no yuuyousei (in Japanese). Nou to hattatsu (Official Journal of the Japanese Society of Child Neurology). 2009;41(4):294–8.
174. Matsuura S, Morimoto Y, Sugimura M, Taki K, Maeda M, Niwa H. [Anesthesia Experience in a Patient with Dentatorubral-Pallidoluysian Atrophy Receiving Ketogenic Diet Therapy for Intractable Epilepsy] Nanchisei tenkan ni taishi keton shoku chiryou wo uketeiru shijoukaku sekikaku tansoukyuu Rui tai ishukushou kanja no masui keiken (in Japanese). Masui (The Japanese Journal of Anesthesiology). 2009;58(6):762–4.
175. Kizuki H. [A Case of Dentatorubral-Pallidoluysian Atrophy (DRPLA) with Recurrent Status Epilepticus Effectively Treated with Levetiracetam] Kurikaesu keiren juuseki hossa wo kitasu shijoukaku sekikaku tansoukyuu Rui tai ishukushou (DRPLA) ni taishite rebechirasetamu ga yuukou de atta 1 rei (in Japanese). Shinyaku to rinshou (Journal of New Remedies & Clinics). 2012;61(4):859–62.
176. Hamada S, Shimakawa S, Satoura S, Naito E, Hashimoto T. [A Case of Dentatorubral-Pallidoluysian Atrophy Effectively Treated with Levetiracetam] Levetiracetam ga chokou shita shijoukaku sekikaku tansoukyuu Rui tai ishukushou no 1 rei (in Japanese). Nou to hattatsu. (Official Journal of the Japanese Society of Child Neurology). 2014;46(6):439–42.
177. Iba Y, Fujita S, Sugimoto K, Saigo K, Okada M, Takemura T. [A Case of Dentatorubral-Pallidoluysian Atrophy with Intractable Epilepsy and Focal Segmental Glomerulosclerosis] Nanchisei tenkan oyobi soujou shikyuutai koukashou wo gappei shita shijoukaku sekikaku tansoukyuu Rui tai ishukushou no 1 rei (in Japanese). Osaka tenkan kenkyuukai zasshi (The Osaka journal of epilepsy research). 2015;26(1):13–8.
178. Uenishi Y, Tsuji T, Takahashi H, Yamamoto M, Sakata M, Ukai S. [A Case of DRPLA Diagnosed After Six Years of Follow-Up] 6 nenkan no keika de shindan kakutei shita DRPLA no 1 rei (in Japanese). Oosaka tenkan kenkyuukai zasshi (The Osaka journal of epilepsy research). 2019;30(1):19–24.
179. Fukuda K, Hirayama A, Degawa H, Tanimura K, Saito K. [A Case of Dentatorubral-Pallidoluysian Atrophy Successfully Treated with Intravenous Sedation] Joumyaku nai chinsei hou no ouyou ga yuukou de atta shijoukaku sekikaku tansoukyuu Rui tai ishukushou no 1 shourei (in Japanese). Shougaisha shika (Journal of the Japanese Society for Disability and Oral Health). 2021;42(3):276–80.
180. Takai E. [A Case of Spinocerebellar Degeneration (Dentatorubral-Pallidoluysian Atrophy) with Oral Intake Resumed After Glottis Closure Surgery] Byouin shika yori seimon heisa jutsugo ni keikou sesshu wo saikai dekita sekizui shounou henseishou (shijoukaku sekikaku tansoukyuu Rui tai ishukushou) no 1 rei (in Japanese). Oosaka fu shikai shikaizasshi (The Journal of Osaka Dental Association). 2021;1(777):22–3.
181. Kimura N, Takahata N, Nishibori K. [An Autopsy Case of Degenerative Myoclonic Epilepsy] Miokurounusu tenkan henseigata no 1 boukenrei (in Japanese).Rinshou shinkei (Clinical Neurology) 1974;14:568–74.
