# Supplementary material for: Epilepsy in dentatorubral–pallidoluysian atrophy: A systematic review and meta‐analysis
Source: Epilepsia. 2025 Oct 28;67(2):696–711. doi: 10.1111/epi.18700 (PMC12927676; doi:10.1111/epi.18700)
Supplement: Supplementary file 1 — Data S1. [file EPI-67-696-s001.zip › epi18700-sup-0001-FigureS1-S14-TableS1-S3@DRPLA_Supplementary1_Document_nk30_hi2_th6_R_nk37_tk4_th1_hi1_ver3.docx]

**Supplementary Document 1**

**in**

**Epilepsy in Dentatorubral-Pallidoluysian Atrophy: A Systematic Review and Meta-Analysis**

**It includes**

**Methods S1-5;**

**Results S1-2;**

**Figures S1-S14; and**

**Tables S2-3.**

**Table S1 (Summary of study characteristics and findings included in the systematic review and meta-analysis) is shown in Supplementary Document 2 separately.**

**References for all included studies are listed in Supplementary Document 3.**

**Methods S1**

**Search terms**

The MEDLINE (PubMed) search terms used in this study were as follows:

(“DRPLA” [tiab] or “Dentatorubral-pallidoluysian atrophy” [tiab])

AND

((“epilepsy” [tiab] or “epilepsies” [tiab]) OR (“epilepsy” [MeSH]) OR (“seizure” [tiab] or “seizures” [tiab]) OR “seizures” [MeSH]))

The Embase search terms used in this study were as follows:

(DRPLA:ti,ab OR ‘Dentatorubral-pallidoluysian atrophy’:ti,ab)

AND

((epilepsy:ti,ab OR epilepsies:ti,ab) OR (epilepsy/exp) OR (seizure:ti,ab OR seizures:ti,ab) OR (seizures/exp))

The CENTRAL search terms used in this study were as follows:

(DRPLA:ti,ab OR “Dentatorubral-pallidoluysian atrophy”:ti,ab)

AND

((epilepsy:ti,ab OR epilepsies:ti,ab) OR ([mh epilepsy]) OR (seizure:ti,ab OR seizures:ti,ab) OR ([mh seizures])).

The ICHUSHI search terms used in this study were as follows:

(歯状核赤核淡蒼球ルイ体萎縮症/TA or DRPLA/TA or "Dentatorubral-pallidoluysian atrophy"/TA) and (てんかん/TA or epilepsy/TA or てんかん/TH or けいれん性発作/TA or seizure/TA or けいれん性発作/TH) and PT=会議録除く

*The meanings of each Japanese term are shown as below:

歯状核赤核淡蒼球ルイ体萎縮症: “Dentatorubral-pallidoluysian atrophy” in Japanese

てんかん: “epilepsy” in Japanese

けいれん性発作: “seizure” in Japanese

会議録除く: “excluding meeting abstracts” in Japanese

**Methods S2**

**Definition of clinicogenomic background characteristics**

**Sex**

Sex was defined as the biological sex reported in each article.

**Age of onset**

Age of onset was defined as the age at which the earliest DRPLA symptom first appeared, as reported in each article. These symptoms included ataxia, myoclonus, choreoathetosis, epileptic seizures, intellectual decline, dementia, personality changes, behavioral disorders, or psychiatric symptoms.

**Diagnostic method**

The diagnostic method was categorized into genetic, histopathological, and clinical based on the diagnostic approach for DRPLA described in each article as follows:

[Genetic]

If the diagnosis was confirmed by demonstrating the CAG triplet repeat expansion through genetic testing, the diagnostic method of the patients was classified as genetic. We included all testing methods for detecting CAG triplet repeat expansion without restriction to a specific technique.

Additionally, if at least one family member had genetic testing confirming the CAG triplet repeat, the diagnostic method of all family members having the symptoms of DRPLA was classified as genetic.

[Histopathological]

If the diagnosis was made by histopathological findings of the brain, the diagnostic method of the patients was classified as histopathological.

[Clinical]

If neither genetic nor histopathological methods were used.

Additionally, if one family member was diagnosed with DRPLA using the histopathological method, the diagnostic method for all other family members showing symptoms of DRPLA, but without genetic testing or histopathological confirmation, was classified as clinical.

**CAG Triplet Repeat Number**

The CAG triplet repeat number was defined as the value reported in each article, as we included all testing methods described without restriction to a specific technique.

**DRPLA-related clinical features (ataxia, choreoathetosis, extrapyramidal signs, cognitive symptoms, psychiatric symptoms)**

**[ataxia]**
We defined cases with ataxia as including descriptions of the term “ataxia”, as well as all derived forms (e.g., ataxias, ataxic), compound expressions, and contextual occurrences.

**[choreoathetosis]**
We defined cases with choreoathetosis as including descriptions of the term “choreoathetosis”, all derivatives of chorea (e.g., choreas, choreatic, choreo-ballism), all derivatives of athetosis (e.g., athetoses, athetoid), compound expressions containing either or both roots, and contextual occurrences.

**[extrapyramidal signs]**
We defined cases with extrapyramidal signs as including descriptions of the term “extrapyramidal”, its major constituent manifestations (such as dystonia, tremor, parkinsonism, rigidity, and bradykinesia), as well as all derived forms, compound expressions, and contextual occurrences.

**[psychiatric symptoms]**
We defined cases with psychiatric symptoms as including descriptions of the listed terms and phrases, as well as all derived forms, compound expressions, and contextual occurrences, such as psychiatric symptom, behavioral change/symptom, irritability, anger, impulsivity, aggressiveness, uncooperativeness, personality/character change, nervousness, panic, excessive involvement in pleasurable activities, euphoria, mood instability/mood change, hyper-erotism/hypersexuality/abnormal sexuality, suicidal tendency, suicidal ideation, suicidal attempt, suicidal behavior, self-harm, positive symptoms, hallucinations, delusions, psychosis, soliloquy, disorganized thinking, disorganized speech, schizophrenia-like symptoms, negative symptoms, blunted affect, asociality, avolition, anhedonia, disorders of diminished motivation, apathy, abulia, and akinetic mutism.

**[cognitive impairment]**
We defined cases with cognitive impairment as including descriptions of the term “cognitive impairment” and a broad range of related conditions, such as intellectual disability, mental retardation, mental deterioration, dementia, learning impairment, concentration disturbance, attention deficit, memory problems, absence of meaningful speech, and an IQ score below 70, together with all derived forms, compound expressions, and contextual occurrences.

**Inheritance pattern**

The inheritance pattern (paternal or maternal) was determined based on descriptions in the text or pedigree in each article. If any paternal or maternal relative was diagnosed with DRPLA using the diagnostic methods previously mentioned, the patient was classified as having paternal or maternal inheritance, respectively. Additionally, if any DRPLA symptom—ataxia, myoclonus, choreoathetosis, epileptic seizures, intellectual decline, dementia, personality changes, behavioral disorders, or psychiatric symptoms—was reported in a paternal or maternal relative, even without a confirmed diagnosis of DRPLA, the patient was also classified as having paternal or maternal inheritance, respectively.

**Methods S3**

**Definition of seizure characteristics**

Based on the ILAE 2017 seizure classification, we categorized reported seizures into the following types: tonic-clonic seizure, myoclonic seizure, tonic seizure, atonic seizure, absence seizure, clonic seizure, focal seizure, and status epilepticus. Additionally, we assessed the presence of photosensitive seizure as triggered seizures. For Japanese literature, two board-certified, native Japanese-speaking epileptologists independently translated seizure types into English. Any discrepancies in their translations were resolved through discussion, and the terminology was standardized in English.

The specific criteria for inclusion/exclusion for each seizure category are detailed below.

[tonic-clonic seizure]

Included:

-bilateral tonic-clonic seizure

-generalized myoclonic seizure evolving generalized tonic-clonic seizure*

-generalized tonic-clonic attack

-generalized tonic-clonic convulsion, generalized tonic-clonic event

-generalized tonic-clonic seizure

-generalized tonic-clonic seizure followed by serial seizures

-grand mal convulsive attack

-grand mal seizure

-partial seizure evolving to secondary generalized tonic-clonic convulsion*

-secondary generalized seizure

-versive seizure with secondary generalized tonic-clonic convulsion*

Excluded:

-generalized convulsion

-generalized convulsive seizure

[myoclonic seizure]

Included:

-absence seizure with eyelid myoclonus*

-atypical absence seizure with myoclonus*

-generalized myoclonic attack

-generalized myoclonic jerk

-generalized myoclonic seizure

-generalized myoclonic seizure evolving generalized tonic-clonic seizure*

-generalized tonic-clonic seizure following trains of bilateral massive myoclonus

-myoclonic absence seizure*

-myoclonic clonic seizure*

-myoclonic jerk

-myoclonic jerk of the upper limbs

-myoclonic seizure with photosensitivity*

-myoclonus to generalized tonic-clonic convulsion

-myoclonic seizure with loss of consciousness

-myoclonic status epilepticus*

-myoclonus status epilepticus*

-tonic-clonic seizures with a myoclonic component

Excluded:

-myoclonus without terms that can indicate any seizures

-unconsciousness accompanied generalized muscle jerk

[tonic seizure]

Included:

-bilateral tonic convulsion

-generalized tonic attack

-generalized tonic convulsion starting from the upper extremities

-generalized tonic drop attack

-tonic convulsion

-tonic seizure with photosensitivity

Excluded:

-tonic-clonic seizure

[atonic seizure]

Included:

-atonic seizure

-atonic drop attack

-possible atonic seizure

Excluded:

-astatic seizure

-fall seizure

-loss of consciousness following a backward fall

[absence seizure]

Included:

-absence seizure with eyelid myoclonus*

-atypical absence seizure with myoclonus*

-atypical absence seizure

-atypical absence

-probable absence status epilepticus*

-myoclonic absence seizure*

-non-typical absence seizure

Excluded:

-loss of consciousness without terms that can indicate any seizures

-seizure with motion arrest and stare

[clonic seizure]

Included:

-generalized clonic convulsion

-clonic convulsion

-clonic seizure

-clonic seizure with photosensitivity

-myoclonic clonic seizure*

-ocular clonic

-clonic convulsion with vocalization

Excluded:

-tonic-clonic seizure

[focal seizure]

Included:

-complex partial seizure

-crying seizure with altered consciousness

-focal complex seizure

-focal seizure

-general and complex partial seizure

-generalized seizure with version

-generalized tonic-clonic seizure starting from facial convulsion

-generalized tonic convulsion starting from the upper extremities

-hemi-clonic seizure

-incompetent-type convulsive seizure

-Jacksonian seizure

-lateralized convulsion

-loss of consciousness following deviation of the eyes

-partial seizure evolving to secondary generalized tonic-clonic convulsion*

-partial seizure with versive

-partial status epilepticus involving the limbs and side of the face with conjugate eye deviation associated with generalization*

-seizure with tonic stiffening of the corner of the mouth and the upper limb

-seizure with version and flexion of the hand

-versive seizure evolving to partial onset generalized tonic-clonic seizure

-versive seizure with secondary generalized tonic-clonic convulsion*

Excluded:

-generalized epileptic seizure with loss of consciousness

-loss of consciousness following a backward fall

-loss of consciousness without terms that can indicate any seizures

-unconsciousness accompanied generalized muscle jerk

[status epilepticus]

Included:

-status epilepticus

-convulsive status epilepticus

-partial status epilepticus involving the limbs and side of the face with conjugate eye deviation associated with generalization*

-probable absence status epilepticus*

-generalized tonic-clonic convulsion following generalized myoclonus status epilepticus*

-myoclonic status epilepticus*

Excluded:

None

[photosensitive seizure]

Included:

-tonic seizure with photosensitivity

-clonic seizure with photosensitivity

-myoclonic seizure with photosensitivity*

-photosensitive seizure

-photostimulation induced seizure

-generalized convulsive during photostimulation

-generalized convulsive seizure due to photic stimulation

Excluded:

None

*: The term is included in two or more seizure categories.

In addition, based on the ILAE 2025 seizure classification, we categorized reported seizures into the following types: generalized seizure, unknown seizure, focal seizure, and unclassified seizure.

The specific criteria for inclusion/exclusion for each seizure category are detailed below.

[Generalized onset seizure]

Included:

-seizures described explicitly as “generalized onset”

-generalized tonic-clonic seizure

-myoclonic seizure

-atonic seizure

-absence seizure

-clonic seizure

[Unknown seizure]

Included:

-seizures for which onset (generalized vs focal) cannot be determined

(For example: “bilateral convulsion,” “loss of consciousness” without any lateralizing or focal signs or features.)

[Focal onset seizure]

Included:

-seizures described explicitly as “focal onset” or “partial onset”

-seizures with lateralizing focal signs or features at onset

-seizures described as “focal to bilateral tonic-clonic” (i.e. secondarily generalized)

[Unspecified seizure type]

Included:

-seizures described only with general terms such as “epileptic seizure,” “convulsion,” “seizure,” without indication of onset or semiology of seizure type

**Methods S4**

**Definition of electrophysiological findings**

**EEG Findings**

We evaluated three EEG patterns: slow wave bursts, photoparoxysmal responses, and interictal epileptiform discharges (IEDs). Slow wave bursts were classified only when explicitly described using the phrase “slow wave bursts.” Similarly, photoparoxysmal responses were classified when described verbatim as “photoparoxysmal responses” or “photic paroxysmal responses.” IEDs were defined as findings described using terms such as "spike," "sharp," "spike-and-wave," "sharp-and-wave," "spike-and-wave complex," "sharp-and-wave complex," "polyspike," "polysharp," "polyspike-and-wave," "polysharp-and-wave," "polyspike-and-wave complex," or "polysharp-and-wave complex."

Only findings explicitly described in the text were included, including instances where only the results were reported without detailed waveform descriptions. EEG waveforms included in the articles were not interpreted independently; instead, we relied entirely on the authors’ original descriptions and classifications. Data were extracted without regard to whether findings were generalized or focal.

**SEP Findings**

We evaluated all SEP findings described in the text, regardless of the site of stimulation, recording site, or recording conditions. Only findings explicitly described in the text were included, along with instances where only the results were mentioned but no specific findings were presented. Even if SEP waveforms were present in the article, we did not interpret them ourselves; instead, we adhered to the authors’ assessments and definitions as reported in each article.

**Methods S5**

**Definition of drug-resistant epilepsy and uncontrollable seizures**

We evaluated outcomes of DRPLA-related epilepsy in the following two ways: (1) drug-resistant epilepsy, and (2) uncontrollable seizures. Drug-resistant epilepsy was defined as patients who did not achieve seizure freedom despite trials of two or more anti-seizure medications, regardless of the types or doses. This definition followed the widely accepted ILAE criteria. On the other hand, uncontrollable seizures were defined more broadly as cases in which seizure freedom was not achieved, irrespective of treatment details, or as cases described in the original reports as “severe,” “intractable,” or “uncontrollable.” In other words, uncontrollable seizures encompassed drug-resistant epilepsy as well as other cases where seizures persisted but information on treatment was insufficient, or where the cases did not strictly meet the drug-resistant epilepsy criteria.

**Results S1**

**Electrophysiology: Systematic review of SEP findings restricted to studies published in English**

A systematic review of SEP findings included 9 studies, reporting on 34 patients with DRPLA, of whom 28 had epilepsy. No study directly compared SEP findings between patients with and without epilepsy. Among patients with DRPLA-related epilepsy, the following abnormalities were reported: prolonged central conduction time (CCT, defined as the latency between cervical spinal cord and primary somatosensory cortex responses) in 2 patients across 2 studies; reduced N20 amplitude (representing primary somatosensory cortex response) in 3 patients from 1 study; prolonged N20 latency in 3 patients from 1 study. Giant SEPs (considered indicative of cortical hyperexcitability) were reported in 2 patients from 1 study, while 3 studies including 16 patients with epilepsy reported absence of giant SEPs.

**Results S2**

**Survival time from the clinical onset of DRPLA: Systematic review restricted to studies published in English**

Regarding survival time from the onset of DRPLA, 237 patients with epilepsy and 76 patients without epilepsy were included in the analysis. The median survival time from onset was 20 years (95% CI: 16.7–23.3) in patients with epilepsy and 16 years (95% CI: 14.5–17.5) in those without epilepsy. **Figure S14** illustrates the Kaplan–Meier curves. The log-rank test failed to demonstrate a significant difference in survival times in patients with epilepsy compared to those without epilepsy (χ² = 1.37, p = 0.24).


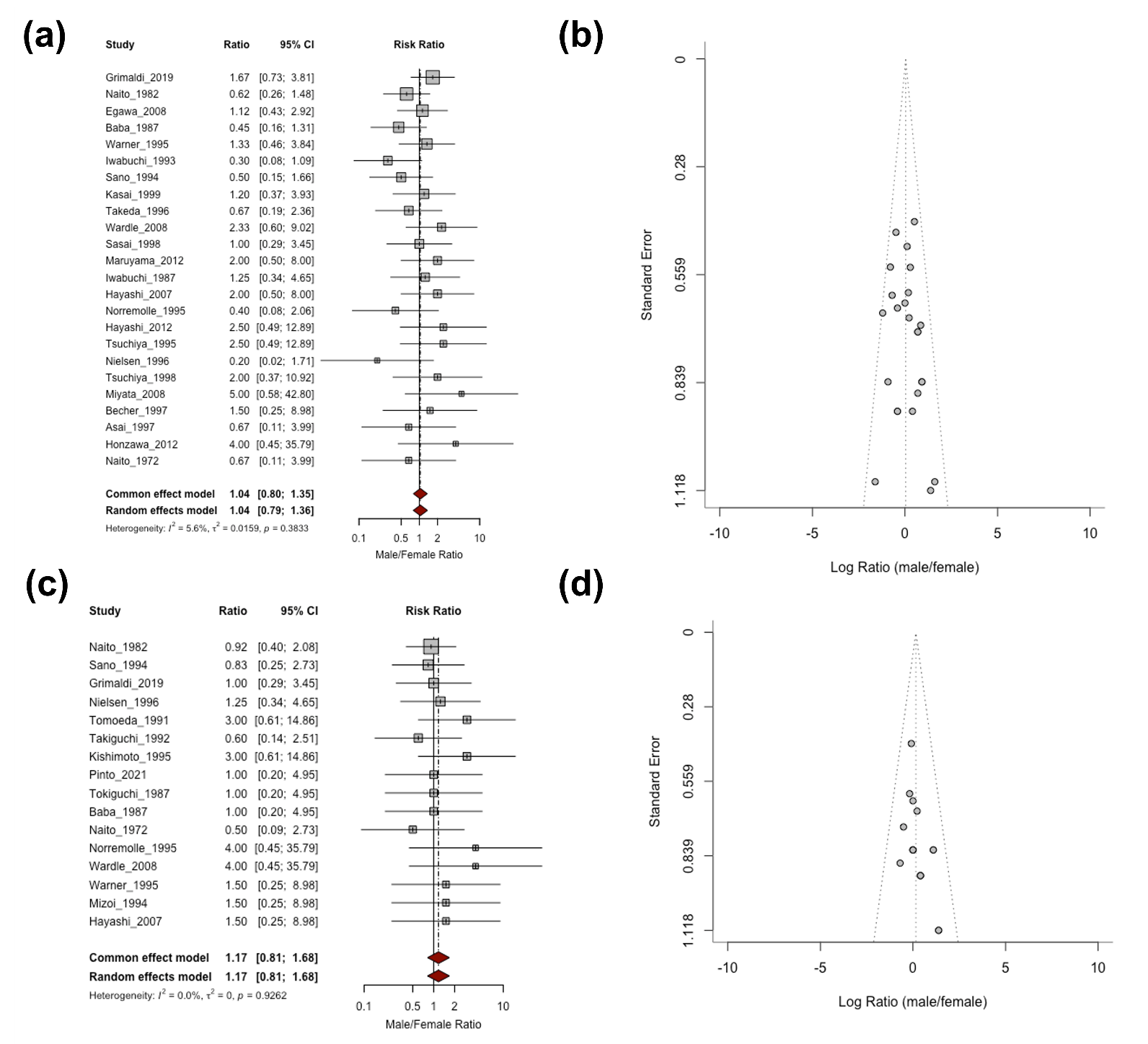


**Figure S1. Meta-analyses** **of the sex ratio (male/female) in DRPLA patients with and without epilepsy.** (a) Forest plot for DRPLA patients with epilepsy. (b) Funnel plot for DRPLA patients with epilepsy. (c) Forest plot for DRPLA patients without epilepsy. (d) Funnel plot for DRPLA patients without epilepsy.


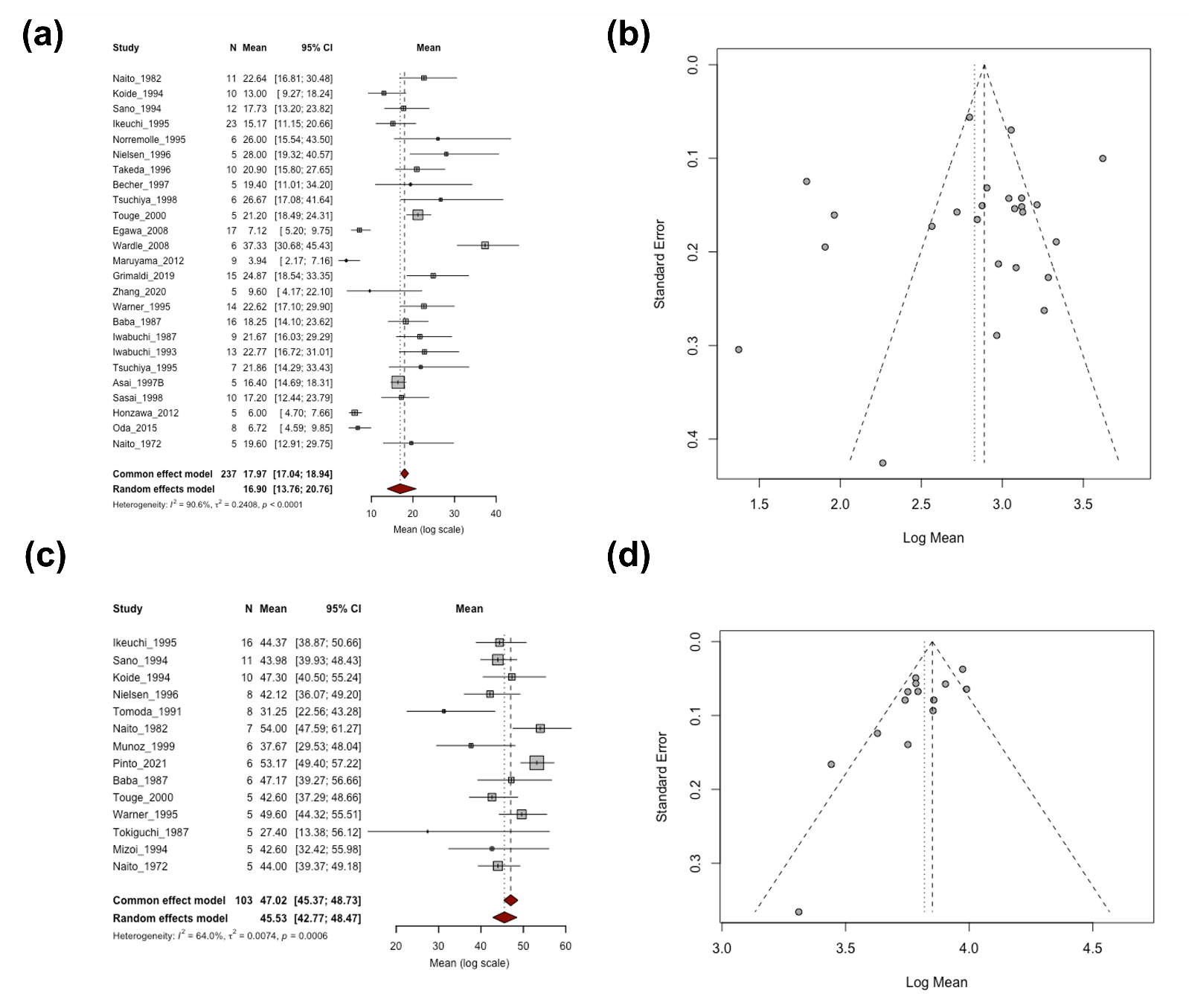


**Figure S2. Meta-analyses of the age onset in DRPLA patients with and without epilepsy.** (a) Forest plot for DRPLA patients with epilepsy. (b) Funnel plot for DRPLA patients with epilepsy. (c) Forest plot for DRPLA patients without epilepsy. (d) Funnel plot for DRPLA patients without epilepsy.


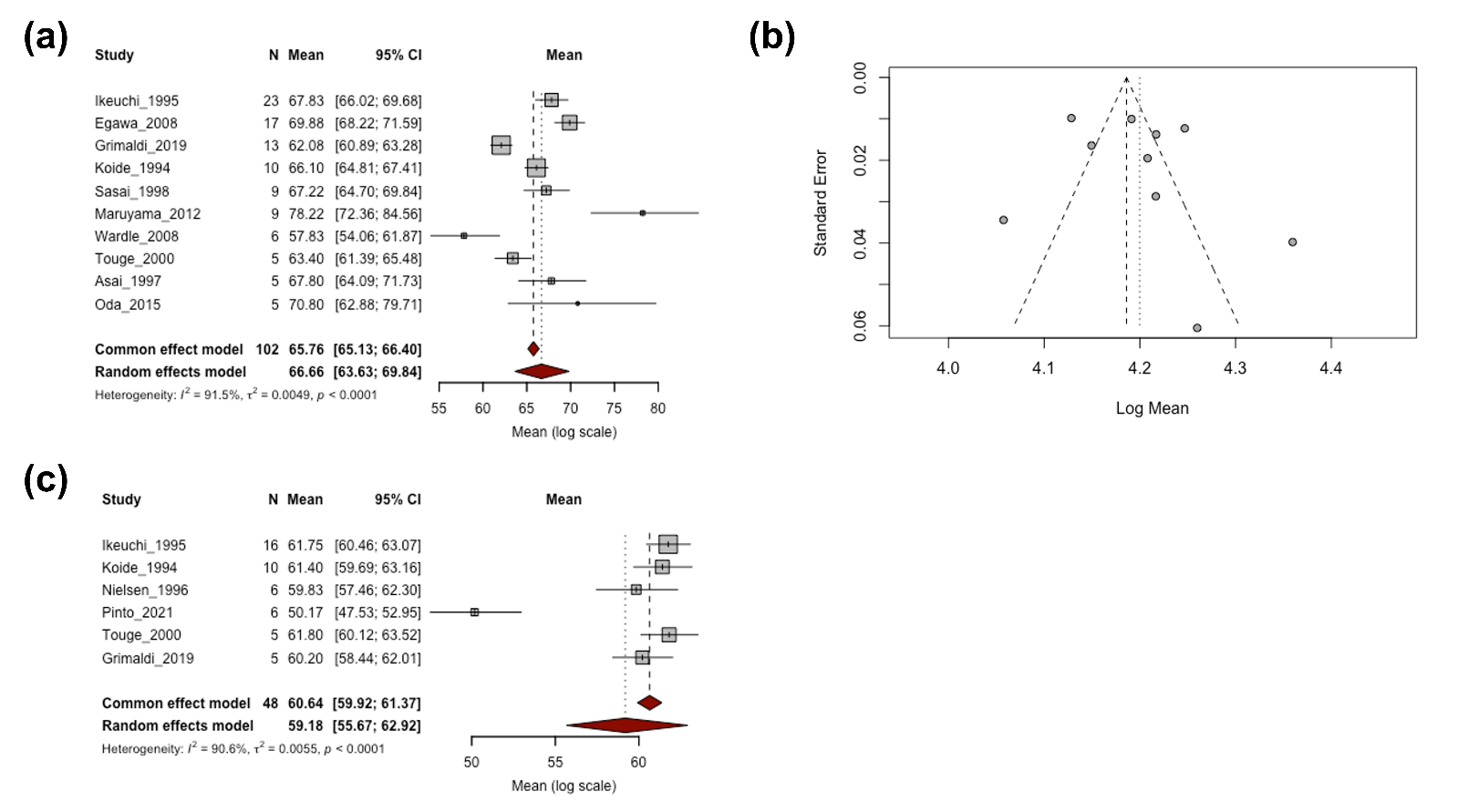


**Figure S3. Meta-analyses of the number of CAG repeats in DRPLA patients with and without epilepsy.** (a) Forest plot for DRPLA patients with epilepsy. (b) Funnel plot for DRPLA patients with epilepsy. (c) Forest plot for DRPLA patients without epilepsy. A funnel plot for DRPLA patients without epilepsy was not created because the number of included studies was less than ten.


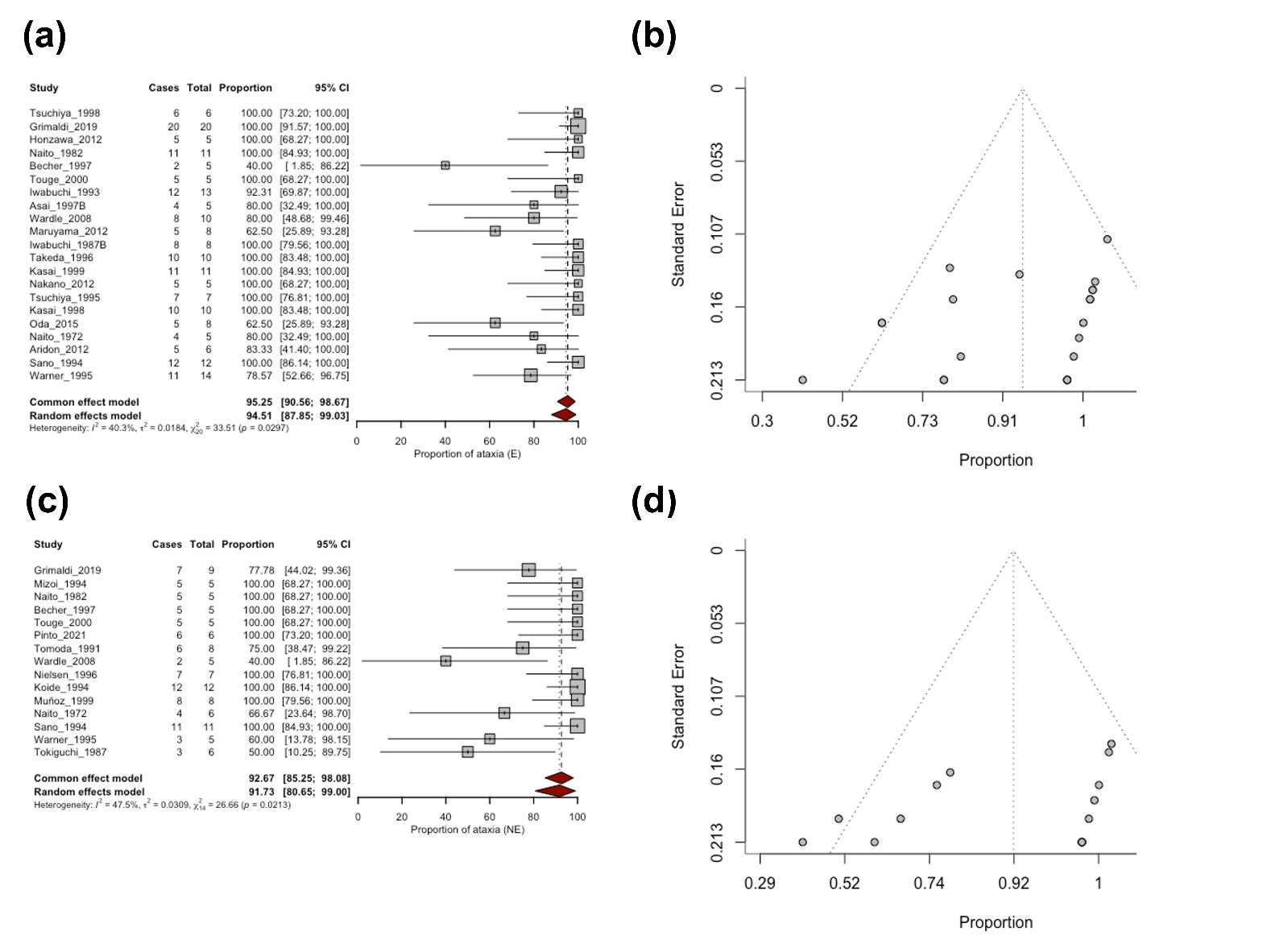


**Figure S4. Meta-analyses of the prevalence of ataxia in DRPLA patients with and without epilepsy.** (a) Forest plot for DRPLA patients with epilepsy. (b) Funnel plot for DRPLA patients with epilepsy. (c) Forest plot for DRPLA patients without epilepsy. (d) Funnel plot for DRPLA patients without epilepsy.


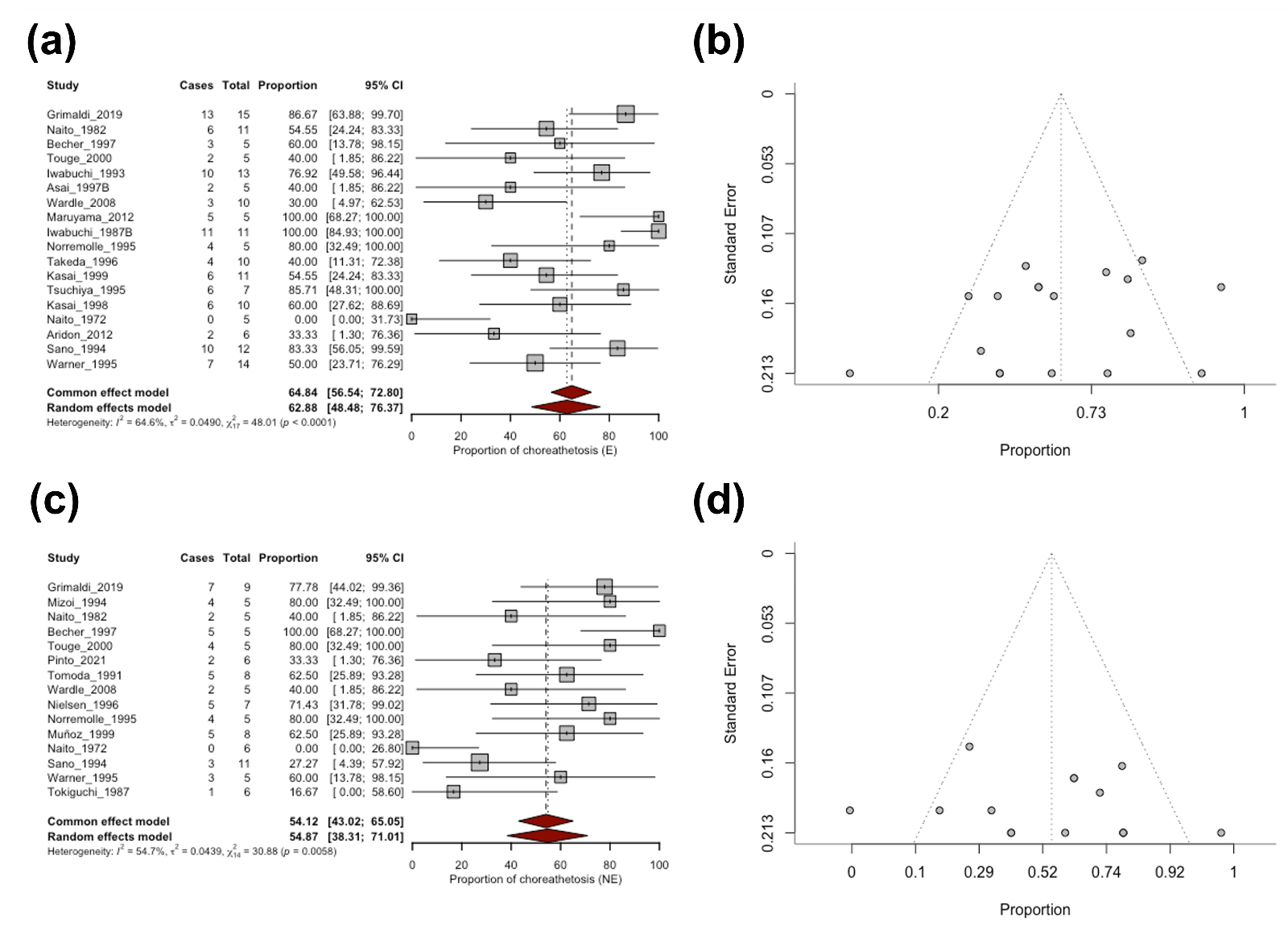


**Figure S5. Meta-analyses of the prevalence of choreoathetosis in DRPLA patients with and without epilepsy.** (a) Forest plot for DRPLA patients with epilepsy. (b) Funnel plot for DRPLA patients with epilepsy. (c) Forest plot for DRPLA patients without epilepsy. (d) Funnel plot for DRPLA patients without epilepsy.


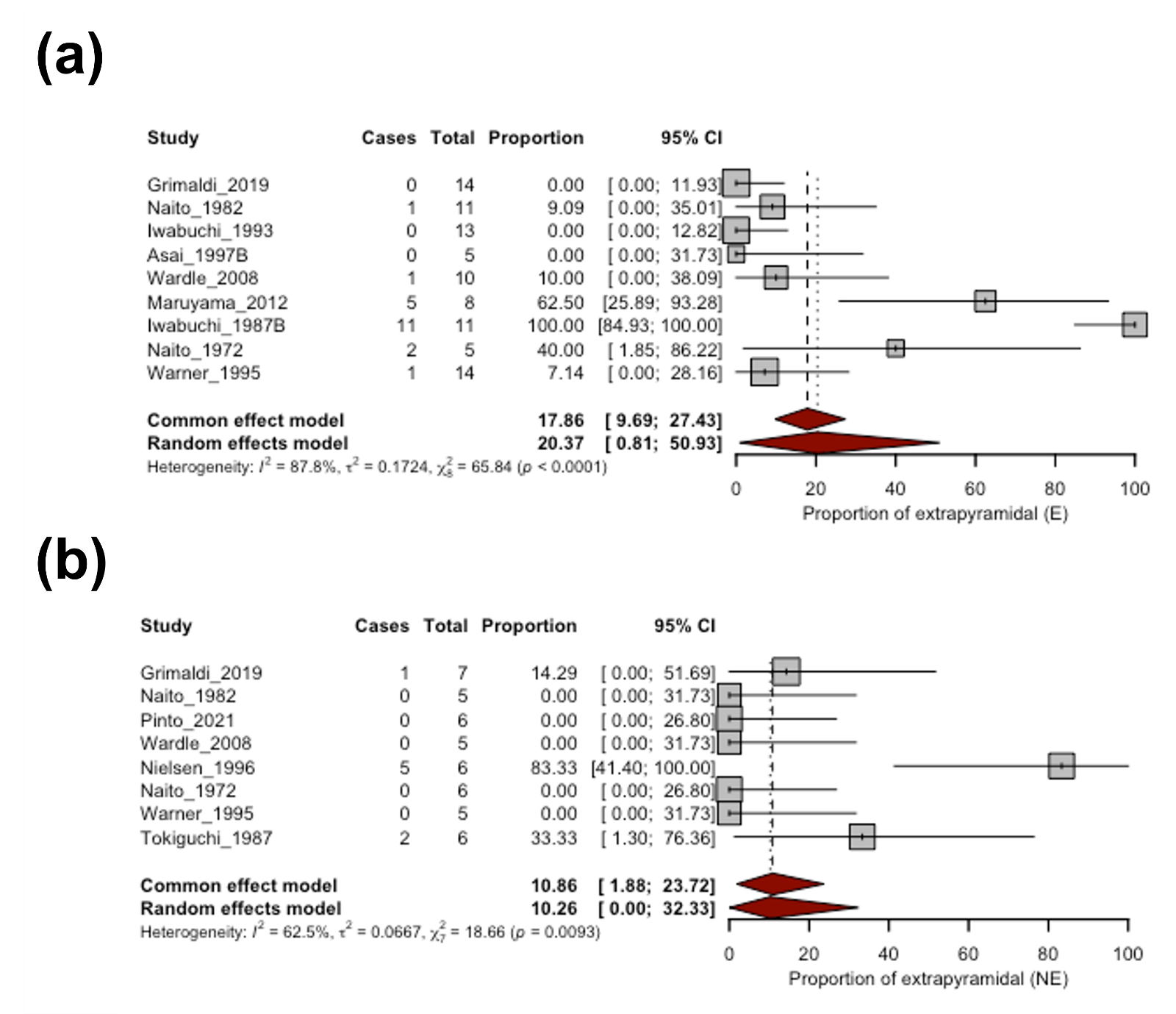


**Figure S6. Meta-analyses of the prevalence of extrapyramidal signs in DRPLA patients with and without epilepsy.** (a) Forest plot for DRPLA patients with epilepsy. (b) Forest plot for DRPLA patients without epilepsy. Funnel plots for DRPLA patients with or without epilepsy were not created because the number of included studies was less than ten.


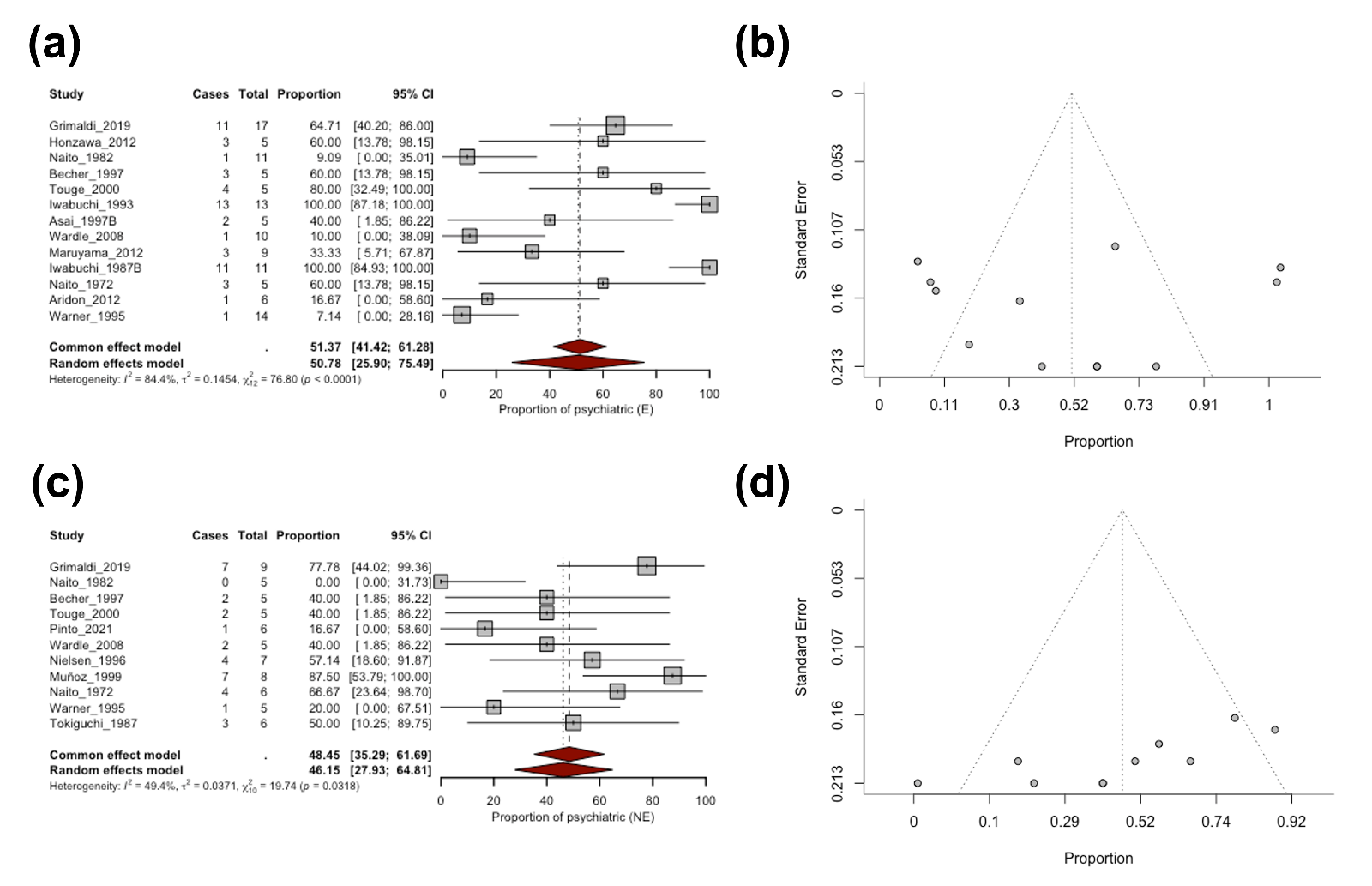


**Figure S7. Meta-analyses of the prevalence of psychiatric symptoms in DRPLA patients with and without epilepsy.** (a) Forest plot for DRPLA patients with epilepsy. (b) Funnel plot for DRPLA patients with epilepsy. (c) Forest plot for DRPLA patients without epilepsy. (d) Funnel plot for DRPLA patients without epilepsy.


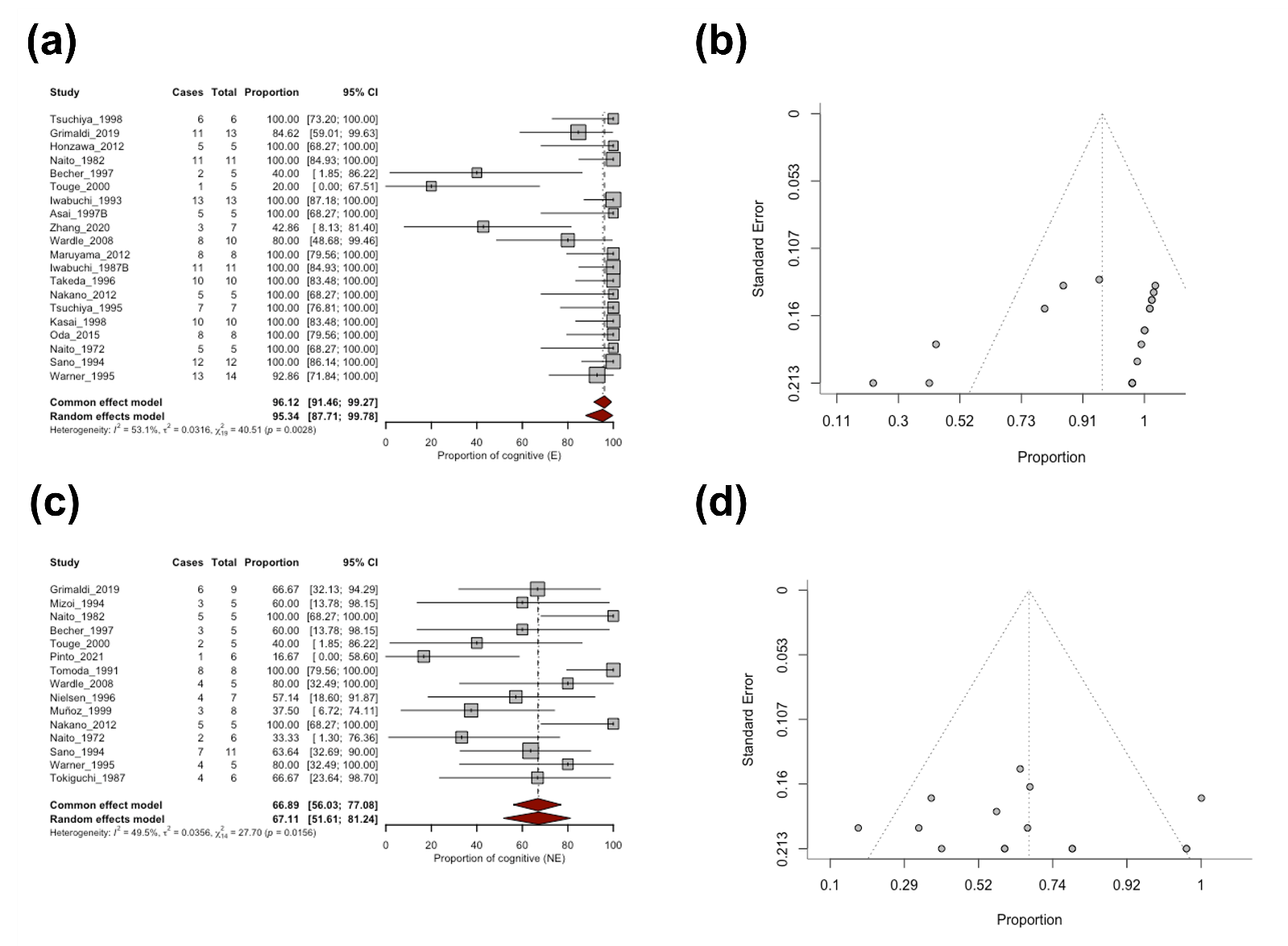


**Figure S8. Meta-analyses of the prevalence of cognitive impairment in DRPLA patients with and without epilepsy.** (a) Forest plot for DRPLA patients with epilepsy. (b) Funnel plot for DRPLA patients with epilepsy. (c) Forest plot for DRPLA patients without epilepsy. (d) Funnel plot for DRPLA patients without epilepsy.


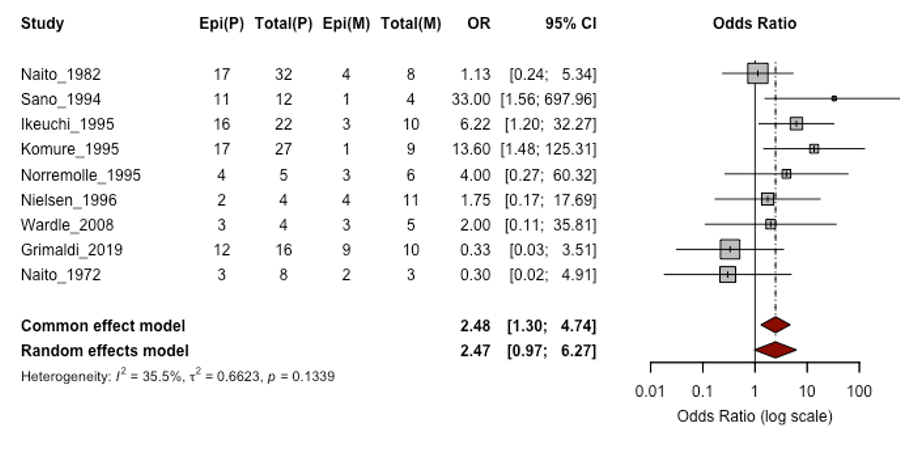


**Figure S9. Meta-analysis of the odds ratio for developing epilepsy in patients with DRPLA according to paternal versus maternal inheritance.** A funnel plot was not created because fewer than ten studies were included. Epi (P): Number of patients with DRPLA-related epilepsy and paternal inheritance. Total (P): Total number of DRPLA patients with paternal inheritance, regardless of epilepsy status. Epi (M): Number of patients with DRPLA-related epilepsy and maternal inheritance. Total (M): Total number of DRPLA patients with maternal inheritance, regardless of epilepsy status.


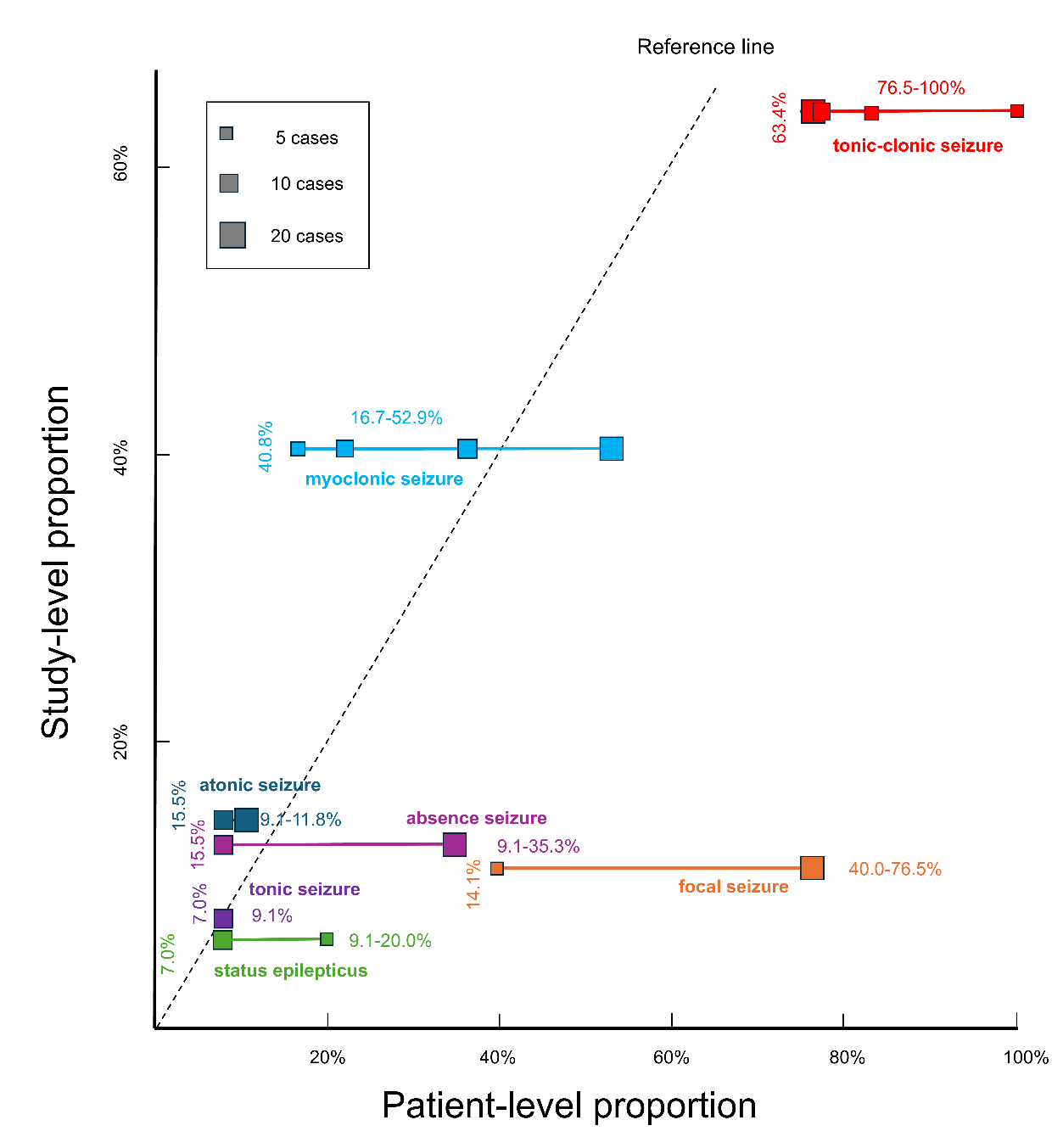


**Figure S10. Relationship between the study-level proportions and the patient-level proportions of each seizure characteristic, restricted to studies published in English.** This figure illustrates the *study-level* and *patient-level proportions* of each seizure characteristic. The *study-level proportion* (plotted on the Y-axis and indicated by the vertical number in each color) is defined as the proportion of studies that reported each specific seizure characteristic among the 71 included in this systematic review. The *patient-level proportion* (plotted on the X-axis and indicated by the horizontal number in each color) is defined as the proportion of patients with each specific seizure characteristic within individual studies. The *patient-level proportion* was calculated only from studies that (1) included five or more patients with DRPLA-related epilepsy and (2) provided data on the presence of the seizure characteristic in question. Each square represents a single study, and the size of the square reflects the total number of reported patients in that study, which serves as the denominator for the patient-level proportion. Each color corresponds to a specific seizure characteristic. A reference line (shown as a black dotted line) indicates where the *study-level proportion* equals the *patient-level proportion*.


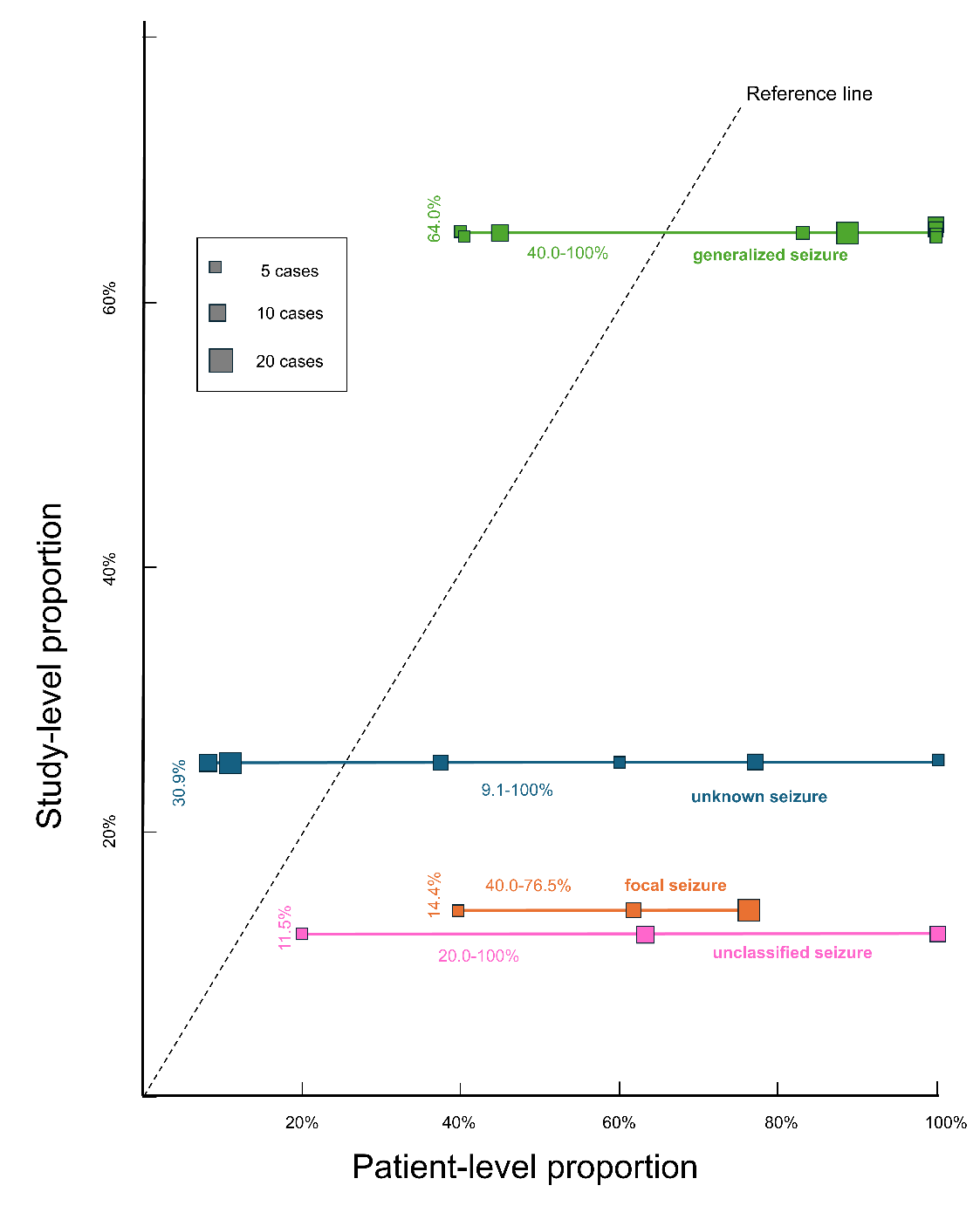


**Figure S11. Relationship between the study-level proportions and the patient-level proportions of each seizure characteristic, based on the ILAE 2025 seizure classification.** This figure illustrates the *study-level* and *patient-level proportions* of each seizure characteristic. The *study-level proportion* (plotted on the Y-axis and indicated by the vertical number in each color) is defined as the proportion of studies that reported each specific seizure characteristic among the 139 included in this systematic review. The *patient-level proportion* (plotted on the X-axis and indicated by the horizontal number in each color) is defined as the proportion of patients with each specific seizure characteristic within individual studies. The *patient-level proportion* was calculated only from studies that (1) included five or more patients with DRPLA-related epilepsy and (2) provided data on the presence of the seizure characteristic in question. Each square represents a single study, and the size of the square reflects the total number of reported patients in that study, which serves as the denominator for the patient-level proportion. Each color corresponds to a specific seizure characteristic. A reference line (shown as a black dotted line) indicates where the *study-level proportion* equals the *patient-level proportion*.


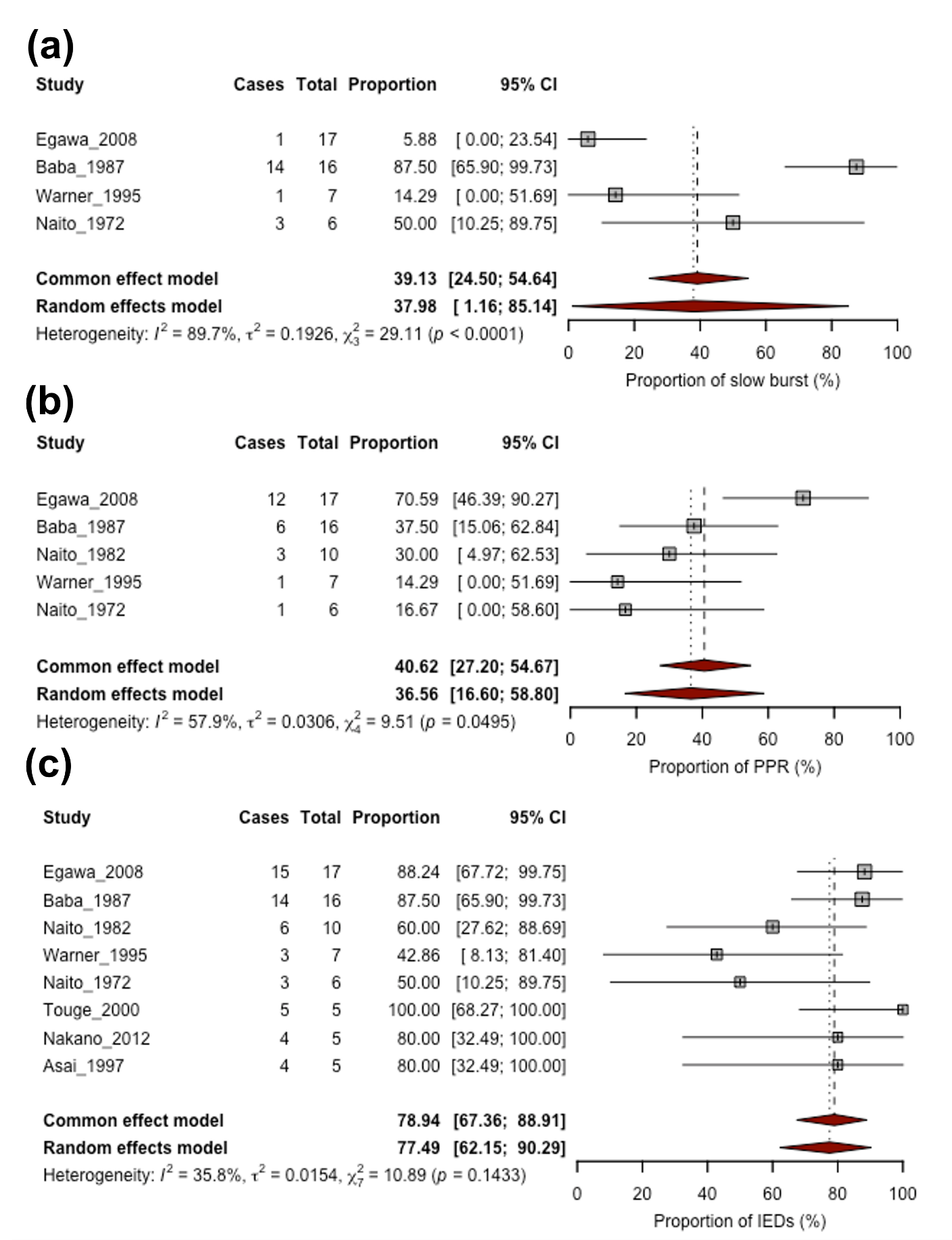


**Figure S12. Meta-analyses of the proportion of EEG findings in patients with DRPLA-related epilepsy.** (a) Forest plot for slow bursts. (b) Funnel plot for photoparoxysmal responses. (c) Forest plot for interictal epileptiform discharges. Funnel plots were not created because the number of included studies was less than ten.


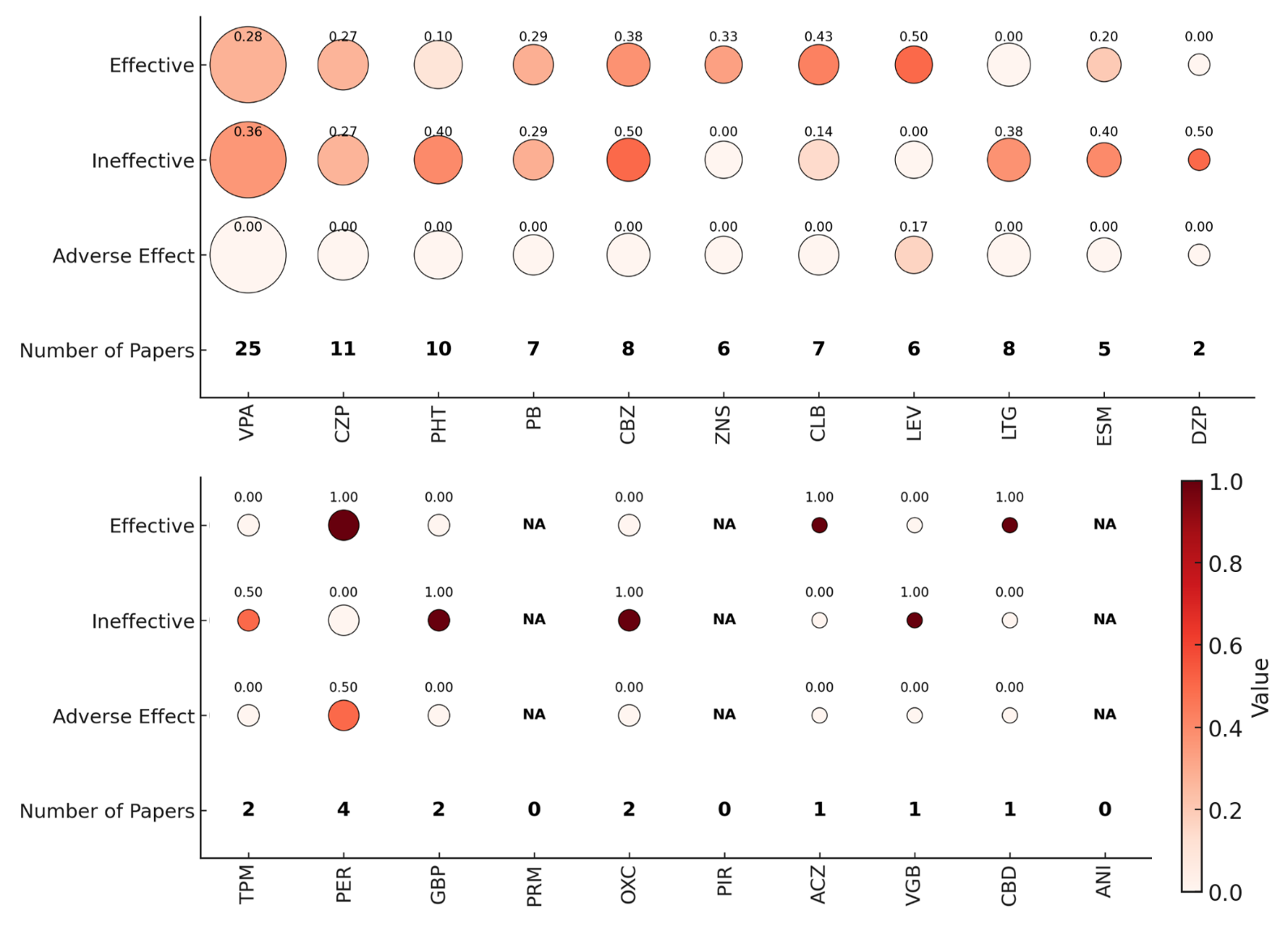


**Figure S13. Relationship between the number of studies reporting the use of specific anti-seizure medications (ASMs) and the number of studies reporting them as effective, ineffective, or associated with adverse effects, restricted to studies published in English.** The size of each circle represents the number of studies reporting the use of that ASM. The numbers shown above each circle indicate the proportion of studies reporting the ASM as effective, ineffective, or associated with adverse effects. The color of each circle also reflects these proportions. ACZ: Acetazolamide. ANI: Aniracetam. CBZ: Carbamazepine. CBD: Cannabidiol. CLB: Clobazam. CZP: Clonazepam. DZP: Diazepam. ESM: Ethosuximide. GBP: Gabapentin. LEV: Levetiracetam. LTG: Lamotrigine. OXC: Oxcarbazepine. PB: Phenobarbital. PER: Perampanel. PHT: Phenytoin. PIR: Piracetam. PRM: Primidone. TPM: Topiramate. VGB: Vigabatrin. VPA: Valproic acid.


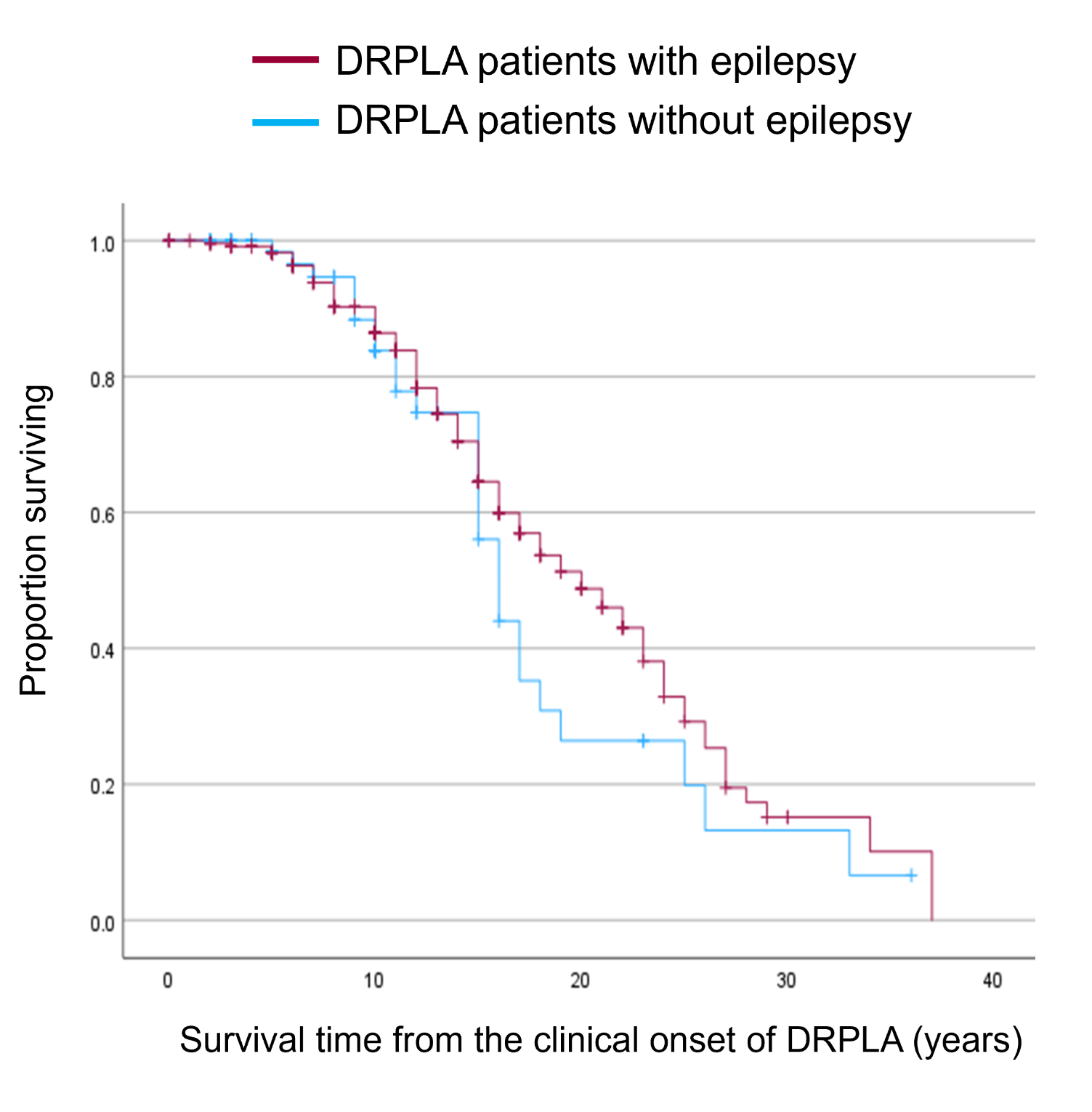


**Figure S14. Kaplan–Meier survival curves in DRPLA patients with and without epilepsy, restricted to studies published in English.** Survival times from the clinical onset of DRPLA were compared between patients with epilepsy (n=237) and those without epilepsy (n=76).

**Table S1** (Summary of study characteristics and findings included in the systematic review and meta-analysis) is shown in **Supplementary Document 2** separately**.**

**Table S2. Overview of the meta-analyses findings, including sensitivity analyses.**

|  | Threshold* | Number of included studies | | Results of meta-analysis | |
| --- | --- | --- | --- | --- | --- |
|  |  | Epilepsy (+) | Epilepsy (-) | Epilepsy (+) | Epilepsy (-) |
| Sex (male/female) | ≥3 | 50 | 24 | 1.04  (95% CI: 0.83–1.31) | 1.11  (95% CI: 0.79–1.55) |
|  | ≥4 | 38 | 20 | 1.02  (95% CI: 0.80–1.30) | 1.07  (95% CI: 0.76–1.51) |
|  | ≥5 | 24 | 16 | 1.04  (95% CI: 0.79–1.36) | 1.17  (95% CI: 0.81–1.68) |
|  | ≥6 | 20 | 11 | 1.03  (95% CI: 0.77–1.38) | 1.04  (95% CI: 0.70–1.55) |
|  | ≥10 | 11 | 3 | 0.89  (95% CI: 0.63–1.26) | 0.91  (95% CI: 0.51–1.65) |
| Onset age of DRPLA | ≥3 | 54 | 22 | 15.04 years old  (95% CI: 12.72–17.77) | 44.94 years old  (95% CI: 42.17–47.90) |
|  | ≥4 | 36 | 19 | 15.47years old  (95% CI: 12.97–18.45) | 46.50 years old  (95% CI: 44.25–48.88) |
|  | ≥5 | 25 | 14 | 16.90 years old  (95% CI: 13.76–20.76) | 45.53 years old  (95% CI: 42.77–48.47) |
|  | ≥6 | 18 | 9 | 17.37 years old  (95% CI: 13.60–22.17) | 45.70 years old  (95% CI: 41.88–49.85) |
|  | ≥10 | 11 | 3 | 17.58 years old  (95% CI: 14.28–21.66) | 44.74 years old  (95% CI: 41.73–47.97) |
| Number of CAG repeats | ≥3 | 29 | 12 | 67.65 repeats  (95% CI: 65.98–69.36) | 60.78 repeats  (95% CI: 58.57–63.08) |
|  | ≥4 | 16 | 7 | 66.53 repeats  (95% CI: 64.51–68.61) | 59.81 repeats  (95% CI: 56.56–63.25) |
|  | ≥5 | 10 | 6 | 66.66 repeats  (95% CI: 63.63–69.84) | 59.18 repeats  (95% CI: 55.67–62.92) |
|  | ≥6 | 7 | 4 | 66.68 repeats  (95% CI: 62.53–71.09) | 58.21 repeats  (95% CI: 52.97–63.96) |
|  | ≥10 | 4 | 2 | 66.38 repeats  (95% CI: 63.16–69.76) | 61.62 repeats  (95% CI: 60.59–62.68) |
| Ataxia | ≥3 | 49 | 21 | 89.89%  (95% CI: 82.45–95.86) | 93.27%  (95% CI: 84.57–99.05) |
|  | ≥4 | 34 | 20 | 92.21%  (95% CI: 85.20–97.52) | 92.82%  (95% CI: 83.71–98.94) |
|  | ≥5 | 21 | 15 | 94.51%  (95% CI: 87.85–99.03) | 91.73%  (95% CI: 80.65–99.00) |
|  | ≥6 | 15 | 9 | 95.39%  (95% CI: 88.61–99.59) | 92.11%  (95% CI: 78.69–99.80) |
|  | ≥10 | 9 | 2 | 97.42%  (95% CI: 91.52–100.00) | 100.00%  (95% CI: 91.70–100.00) |
| Choreoathetosis | ≥3 | 39 | 19 | 53.94%  (95% CI: 41.85–65.85) | 57.78%  (95% CI: 40.96–73.90) |
|  | ≥4 | 28 | 18 | 57.08%  (95% CI: 44.35–69.45) | 55.47%  (95% CI: 38.68–71.77) |
|  | ≥5 | 18 | 15 | 62.88%  (95% CI: 48.48–76.37) | 54.87%  (95% CI: 38.31–71.01) |
|  | ≥6 | 12 | 8 | 66.05%  (95% CI: 51.05–79.77) | 42.86%  (95% CI: 21.95–64.93) |
|  | ≥10 | 10 | NA | 66.58%  (95% CI: 50.37–81.23) | NA |
| Extrapyramidal signs | ≥3 | 25 | 10 | 20.33%  (95% CI: 6.41–37.79) | 13.05%  (95% CI: 0.20–34.97) |
|  | ≥4 | 16 | 10 | 25.18%  (95% CI: 6.84–48.24) | 13.05%  (95% CI: 0.20–34.97) |
|  | ≥5 | 9 | 8 | 20.37%  (95% CI: 0.81–50.93) | 10.26%  (95% CI: 0.00–32.33) |
|  | ≥6 | 7 | 5 | 22.11%  (95% CI: 0.19–58.33) | 20.22%  (95% CI: 0.00–55.89) |
|  | ≥10 | 6 | NA | 16.83%  (95% CI: 0.00–55.10) | NA |
| Psychiatric symptoms | ≥3 | 31 | 15 | 43.77%  (95% CI: 26.84–61.29) | 51.46%  (95% CI: 34.52–68.27) |
|  | ≥4 | 21 | 14 | 49.45%  (95% CI: 29.06–69.91) | 50.67%  (95% CI: 33.01–68.27) |
|  | ≥5 | 13 | 11 | 50.78%  (95% CI: 25.90–75.49) | 46.15%  (95% CI: 27.93–64.81) |
|  | ≥6 | 8 | 6 | 45.78%  (95% CI: 13.35–80.06) | 61.64%  (95% CI: 40.43–81.12) |
|  | ≥10 | 6 | NA | 52.29%  (95% CI: 11.75–91.26) | NA |
| Cognitive impairment | ≥3 | 50 | 19 | 97.69%  (95% CI: 94.08–99.80) | 67.86%  (95% CI: 52.07–82.18) |
|  | ≥4 | 34 | 18 | 96.78%  (95% CI: 91.89–99.72) | 66.06%  (95% CI: 50.00–80.76) |
|  | ≥5 | 20 | 15 | 95.34%  (95% CI: 87.71–99.78) | 67.11%  (95% CI: 51.61–81.24) |
|  | ≥6 | 14 | 8 | 97.20%  (95% CI: 91.38–100.00) | 58.52%  (95% CI: 37.33–78.43) |
|  | ≥10 | 9 | NA | 97.59%  (95% CI: 92.46–100.00) | NA |
| Paternal inheritance | ≥3 | 11 | | Odds ratio: 2.45 (95% CI: 1.08–5.54) | |
|  | ≥4 | 10 | | Odds ratio: 2.58 (95% CI: 1.09–6.08) | |
|  | ≥5 | 9 | | Odds ratio: 2.47 (95% CI: 0.97–6.27) | |
|  | ≥6 | 6 | | Odds ratio: 3.08 (95% CI: 0.93–10.23) | |
|  | ≥10 | 5 | | Odds ratio: 3.50 (95% CI: 0.81–15.22) | |
| Slow bursts in EEG | ≥3 | 11 | NA | 34.72%  (95% CI: 8.74–65.30) | NA |
|  | ≥4 | 8 | NA | 24.63%  (95% CI: 1.10–59.11) | NA |
|  | ≥5 | 4 | NA | 37.98%  (95% CI: 1.16–85.14) | NA |
|  | ≥6 | 4 | NA | 37.98%  (95% CI: 1.16–85.14) | NA |
|  | ≥10 | 2 | NA | 44.51%  (95% CI: 0.00–100.00) | NA |
| Photoparoxysmal responses in EEG | ≥3 | 12 | NA | 33.38%  (95% CI: 15.94–52.83) | NA |
|  | ≥4 | 8 | NA | 37.02%  (95% CI: 15.50–60.97) | NA |
|  | ≥5 | 5 | NA | 36.56%  (95% CI: 16.60–58.80) | NA |
|  | ≥6 | 5 | NA | 36.56%  (95% CI: 16.60–58.80) | NA |
|  | ≥10 | 3 | NA | 47.36%  (95% CI: 23.02–72.32) | NA |
| Interictal epileptiform discharges in EEG | ≥3 | 20 | NA | 80.47%  (95% CI: 68.77–90.45) | NA |
|  | ≥4 | 14 | NA | 79.02%  (95% CI: 65.36–90.53) | NA |
|  | ≥5 | 8 | NA | 77.49%  (95% CI: 62.15–90.29) | NA |
|  | ≥6 | 5 | NA | 71.52%  (95% CI: 50.77–88.93) | NA |
|  | ≥10 | 3 | NA | 81.76%  (95% CI: 64.28–94.85) | NA |

*: Number of patients reported for each outcome in each included study**.**

**Table S3. Overview of the meta-analysis findings, including sensitivity analyses restricted to English-language studies.**

|  | Threshold* | Number of included studies | | Results of meta-analysis | |
| --- | --- | --- | --- | --- | --- |
|  |  | Epilepsy (+) | Epilepsy (-) | Epilepsy (+) | Epilepsy (-) |
| Sex (male/female) | ≥3 | 33 | 16 | 1.13  (95% CI: 0.86–1.48) | 1.29  (95% CI: 0.88–1.91) |
|  | ≥4 | 26 | 13 | 1.16  (95% CI: 0.87–1.54) | 1.25  (95% CI: 0.83–1.87) |
|  | ≥5 | 17 | 11 | 1.19  (95% CI: 0.87–1.61) | 1.32  (95% CI: 0.87–2.01) |
|  | ≥6 | 16 | 7 | 1.18  (95% CI: 0.86–1.61) | 1.17  (95% CI: 0.73–1.76) |
|  | ≥10 | 8 | 3 | 1.05  (95% CI: 0.73–1.52) | 0.91  (95% CI: 0.51–1.65) |
| Onset age of DRPLA | ≥3 | 35 | 15 | 14.74 years old  (95% CI: 11.91–18.23) | 44.66 years old  (95% CI: 41.01–48.65) |
|  | ≥4 | 26 | 12 | 15.84 years old  (95% CI: 12.71–19.74) | 46.54 years old  (95% CI: 43.44–49.87) |
|  | ≥5 | 17 | 10 | 18.23 years old  (95% CI: 14.23–23.36) | 45.74 years old  (95% CI: 42.31–49.45) |
|  | ≥6 | 13 | 8 | 17.85 years old  (95% CI: 13.12–24.28) | 45.35 years old  (95% CI: 41.06–50.09) |
|  | ≥10 | 8 | 3 | 16.96 years old  (95% CI: 12.77–22.54) | 44.74 years old  (95% CI: 41.73–47.97) |
| Number of CAG repeats | ≥3 | 19 | 12 | 66.88 repeats  (95% CI: 64.75–69.08) | 60.78 repeats  (95% CI: 58.57–63.08) |
|  | ≥4 | 10 | 7 | 66.38 repeats  (95% CI: 63.11–69.82) | 59.81 repeats  (95% CI: 56.56–63.25) |
|  | ≥5 | 7 | 6 | 66.11 repeats  (95% CI: 61.91–70.59) | 59.18 repeats  (95% CI: 55.67–62.92) |
|  | ≥6 | 6 | 4 | 66.60 repeats  (95% CI: 61.65–71.95) | 58.21 repeats  (95% CI: 52.97–63.96) |
|  | ≥10 | 4 | 2 | 66.38 repeats  (95% CI: 63.16–69.76) | 61.62 repeats  (95% CI: 60.59–62.68) |
| Ataxia | ≥3 | 28 | 16 | 95.22%  (95% CI: 88.48–99.48) | 94.70%  (95% CI: 85.48–99.86) |
|  | ≥4 | 23 | 15 | 94.38%  (95% CI: 86.56–99.38) | 94.18%  (95% CI: 84.35–99.81) |
|  | ≥5 | 14 | 12 | 95.17%  (95% CI: 86.69–99.88) | 94.55%  (95% CI: 84.07–99.98) |
|  | ≥6 | 11 | 7 | 96.02%  (95% CI: 88.14–99.99) | 97.10%  (95% CI: 88.29–100.00) |
|  | ≥10 | 7 | 2 | 97.45%  (95% CI: 89.74–100.00) | 100.00%  (95% CI: 91.70–100.00) |
| Choreoathetosis | ≥3 | 23 | 15 | 61.31%  (95% CI: 49.70–72.44) | 60.90%  (95% CI: 45.06–75.87) |
|  | ≥4 | 20 | 14 | 62.63%  (95% CI: 51.75–73.02) | 58.40%  (95% CI: 42.73–73.42) |
|  | ≥5 | 13 | 12 | 62.92%  (95% CI: 48.97–76.03) | 61.54%  (95% CI: 47.13–75.17) |
|  | ≥6 | 9 | 6 | 59.52%  (95% CI: 43.87–74.38) | 55.58%  (95% CI: 37.77–72.80) |
|  | ≥10 | 7 | NA | 58.91%  (95% CI: 41.82–75.10) | NA |
| Extrapyramidal signs | ≥3 | 12 | 7 | 22.76%  (95% CI: 6.38–43.43) | 16.27%  (95% CI: 0.00–47.83) |
|  | ≥4 | 9 | 7 | 25.48%  (95% CI: 6.76–49.06) | 16.27%  (95% CI: 0.00–47.83) |
|  | ≥5 | 5 | 6 | 12.28%  (95% CI: 0.20–33.50) | 9.76%  (95% CI: 0.00–38.78) |
|  | ≥6 | 5 | 3 | 12.28%  (95% CI: 0.20–33.50) | 26.70%  (95% CI: 0.00–82.86) |
|  | ≥10 | 4 | NA | 4.84%  (95% CI: 0.02–14.19) | NA |
| Psychiatric symptoms | ≥3 | 15 | 12 | 41.00%  (95% CI: 20.91–62.40) | 45.59%  (95% CI: 27.21–64.47) |
|  | ≥4 | 13 | 11 | 42.89%  (95% CI: 21.48–65.47) | 44.21%  (95% CI: 25.01–64.14) |
|  | ≥5 | 8 | 9 | 30.93%  (95% CI: 11.79–53.41) | 42.96%  (95% CI: 21.50–65.58) |
|  | ≥6 | 6 | 4 | 22.34%  (95% CI: 5.44–44.63) | 62.26%  (95% CI: 31.11–89.40) |
|  | ≥10 | 4 | NA | 20.96%  (95% CI: 0.84–52.78) | NA |
| Cognitive impairment | ≥3 | 29 | 15 | 95.51%  (95% CI: 89.07–99.53) | 68.15%  (95% CI: 49.51–84.71) |
|  | ≥4 | 23 | 14 | 93.87%  (95% CI: 85.70–99.21) | 65.82%  (95% CI: 46.74–83.02) |
|  | ≥5 | 13 | 12 | 90.13%  (95% CI: 76.65–98.93) | 70.32%  (95% CI: 52.11–86.22) |
|  | ≥6 | 10 | 6 | 94.84%  (95% CI: 85.48–99.92) | 60.76%  (95% CI: 34.45–84.57) |
|  | ≥10 | NA | NA | NA | NA |
| Paternal inheritance | ≥3 | 8 | | Odds ratio: 2.98 (95% CI: 1.17–7.60) | |
|  | ≥4 | 8 | | Odds ratio: 2.98 (95% CI: 1.17–7.60) | |
|  | ≥5 | 8 | | Odds ratio: 2.98 (95% CI: 1.17–7.60) | |
|  | ≥6 | 6 | | Odds ratio: 3.08 (95% CI: 0.93–10.23) | |
|  | ≥10 | 5 | | Odds ratio: 3.50 (95% CI: 0.81–15.22) | |
| Slow bursts in EEG | ≥3 | 4 | NA | 21.25%  (95% CI: 0.00–65.67) | NA |
|  | ≥4 | 3 | NA | 4.93%  (95% CI: 0.00–19.26) | NA |
|  | ≥5 | 2 | NA | 7.33%  (95% CI: 0.00–23.30) | NA |
|  | ≥6 | 2 | NA | 7.33%  (95% CI: 0.00–23.30) | NA |
|  | ≥10 | 1 | NA | NA | NA |
| Photoparoxysmal responses in EEG | ≥3 | 7 | NA | 46.17%  (95% CI: 20.64–72.57) | NA |
|  | ≥4 | 5 | NA | 49.52%  (95% CI: 48.11–81.10) | NA |
|  | ≥5 | 3 | NA | 46.81%  (95% CI: 9.13–75.67) | NA |
|  | ≥6 | 3 | NA | 46.81%  (95% CI: 9.13–75.67) | NA |
|  | ≥10 | 2 | NA | 52.03%  (95% CI: 14.60–88.31) | NA |
| Interictal epileptiform discharges in EEG | ≥3 | 9 | NA | 81.84%  (95% CI: 62.30–96.32) | NA |
|  | ≥4 | 8 | NA | 79.49%  (95% CI: 58.40–95.45) | NA |
|  | ≥5 | 5 | NA | 77.26%  (95% CI: 54.57–94.64) | NA |
|  | ≥6 | 2 | NA | 77.06%  (95% CI: 45.49–98.25) | NA |
|  | ≥10 | 2 | NA | 77.06%  (95% CI: 45.49–98.25) | NA |

*: Number of patients reported for each outcome in each included study**.**
